# Tumor-immune partitioning and clustering algorithm for identifying tumor-immune cell spatial interaction signatures within the tumor microenvironment

**DOI:** 10.1371/journal.pcbi.1012707

**Published:** 2025-02-18

**Authors:** Mai Chan Lau, Jennifer Borowsky, Juha P. Väyrynen, Koichiro Haruki, Melissa Zhao, Andressa Dias Costa, Simeng Gu, Annacarolina da Silva, Tomotaka Ugai, Kota Arima, Minh N. Nguyen, Yasutoshi Takashima, Joe Yeong, David Tai, Tsuyoshi Hamada, Jochen K. Lennerz, Charles S. Fuchs, Catherine J. Wu, Jeffrey A. Meyerhardt, Shuji Ogino, Jonathan A. Nowak

**Affiliations:** 1 Bioinformatics Institute (BII), Agency for Science, Technology and Research (A* STAR), Singapore, Republic of Singapore; 2 Singapore Immunology Network (SIgN), Agency for Science, Technology and Research (A* STAR), Singapore, Republic of Singapore; 3 Department of Pathology, Program in MPE Molecular Pathological Epidemiology, Brigham and Women’s Hospital and Harvard Medical School, Boston, Massachusetts, United States of America; 4 Conjoint Gastroenterology Department, QIMR Berghofer Medical Research Institute, Herston, Queensland, Australia; 5 Department of Medical Oncology, Dana-Farber Cancer Institute and Harvard Medical School, Boston, Massachusetts, United States of America; 6 Cancer and Translational Medicine Research Unit, Medical Research Center Oulu, Oulu University Hospital and University of Oulu, Oulu, Finland; 7 Department of Surgery, The Jikei University School of Medicine, Tokyo, Japan; 8 Department of Epidemiology, Harvard T.H. Chan School of Public Health, Boston, Massachusetts, United States of America; 9 Division of Pathology, Singapore General Hospital, Singapore, Republic of Singapore; 10 Integrative Biology for Theranostics, Institute of Molecular Cell Biology, Agency of Science, Technology and Research (A* STAR), Singapore, Republic of Singapore; 11 Division of Medical Oncology, National Cancer Centre Singapore, Singapore, Republic of Singapore; 12 Duke NUS Medical School, Singapore, Republic of Singapore; 13 BostonGene; Waltham, Boston, Massachusetts, United States of America; 14 Genentech, South San Francisco, California, United States of America; 15 Department of Medical Oncology, Dana-Farber Cancer Institute, Boston, Massachusetts, United States of America; 16 Department of Medicine, Brigham and Women’s Hospital, Harvard Medical School, Boston, Massachusetts, United States of America; 17 Cancer Vaccine Center, Dana-Farber Cancer Institute, Boston, Massachusetts, United States of America; 18 Broad Institute of MIT and Harvard, Cambridge, Massachusetts, United States of America; 19 Cancer Immunology and Cancer Epidemiology Programs, Dana-Farber Harvard Cancer Center, Boston, Massachusetts, United States of America; Harbin Institute of Technology, CHINA

## Abstract

**Background:**

Growing evidence supports the importance of characterizing the organizational patterns of various cellular constituents in the tumor microenvironment in precision oncology. Most existing data on immune cell infiltrates in tumors, which are based on immune cell counts or nearest neighbor-type analyses, have failed to fully capture the cellular organization and heterogeneity.

**Methods:**

We introduce a computational algorithm, termed Tumor-Immune Partitioning and Clustering (TIPC), that jointly measures immune cell partitioning between tumor epithelial and stromal areas and immune cell clustering versus dispersion. As proof-of-principle, we applied TIPC to a prospective cohort incident tumor biobank containing 931 colorectal carcinoma cases. TIPC identified tumor subtypes with unique spatial patterns between tumor cells and T lymphocytes linked to certain molecular pathologic and prognostic features. T lymphocyte identification and phenotyping were achieved using multiplexed (multispectral) immunofluorescence. In a separate hepatocellular carcinoma cohort, we replaced the stromal component with specific immune cell types—CXCR3^+^CD68^+^ or CD8+—to profile their spatial relationships with CXCL9^+^CD68^+^ cells.

**Results:**

Six unsupervised TIPC subtypes based on T lymphocyte distribution patterns were identified, comprising two cold and four hot subtypes. Three of the four hot subtypes were associated with significantly longer colorectal cancer (CRC)-specific survival compared to a reference cold subtype. Our analysis showed that variations in T-cell densities among the TIPC subtypes did not strictly correlate with prognostic benefits, underscoring the prognostic significance of immune cell spatial patterns. Additionally, TIPC revealed two spatially distinct and cell density-specific subtypes among microsatellite instability-high colorectal cancers, indicating its potential to upgrade tumor subtyping. TIPC was also applied to additional immune cell types, eosinophils and neutrophils, identified using morphology and supervised machine learning; here two tumor subtypes with similarly low densities, namely ‘cold, tumor-rich’ and ‘cold, stroma-rich’, exhibited differential prognostic associations. Lastly, we validated our methods and results using The Cancer Genome Atlas colon and rectal adenocarcinoma data (n = 570). Moreover, applying TIPC to hepatocellular carcinoma cases (n = 27) highlighted critical cell interactions like CXCL9-CXCR3 and CXCL9-CD8.

**Conclusions:**

Unsupervised discoveries of microgeometric tissue organizational patterns and novel tumor subtypes using the TIPC algorithm can deepen our understanding of the tumor immune microenvironment and likely inform precision cancer immunotherapy.

## Author summary

We have developed a computational tool called the Tumor-Immune Partitioning and Clustering (TIPC) algorithm, designed to reveal the intricate organization of immune cells within the tumor microenvironment. Traditionally, studies have mainly focused on counting these cells or examining their proximity to one another. Those approaches often underestimate the complex roles of immune cells in tumors. With TIPC, we have uncovered distinct patterns in the arrangement of immune cells across different tumor regions. This advancement has enabled us to identify tumor subtypes that were previously undetectable with existing methods. Our new method can determine which tumors are likely to have longer survival rates or respond better to immunotherapy, based on the layout of immune cells rather than merely their numbers. This breakthrough has significant implications for cancer research, highlighting the importance of understanding the spatial patterns of immune cells. Such knowledge is crucial for selecting appropriate patients for specific treatments and for assessing the potential effectiveness of immunotherapy. By tailoring treatment plans to the unique cellular landscapes of each tumor, we can potentially improve outcomes and provide more personalized and effective cancer care.

## Introduction

Increasing evidence indicates that not only immune cell abundance but also the organization of neoplastic and immune cells plays a crucial role in tumor evolution, treatment response, and outcome [1–4]. Recent advances in digital imaging, machine learning, and multiplexed tissue-based biomarker assays can generate spatially resolved data mapping the tumor microenvironment (TME) at single-cell resolution [5–10]. While these approaches provide highly granular data, analysis methods for abstracting tumor-immune cell interactions into clinically useful measures such as prognostic and predictive markers remain largely unrealized.

Initial efforts to characterize immune cells in the TME focused primarily on measuring the density of immune cells [5,8,9] or assessing the density of these cells at specified distances from the nearest tumor cells [6,7]. However, in tumors such as melanoma [11] and colorectal cancer (CRC) [12], it has long been recognized that immune cell densities are often higher at the tumor periphery. Furthermore, in CRC, multiple patterns of lymphocytic response can be qualitatively identified and harbor prognostic significance [13,14]. These findings suggest that the inherent organization of immune cells with respect to neoplastic cells can provide critical information regarding effectiveness of the anti-tumor response.

**Fig 1:**
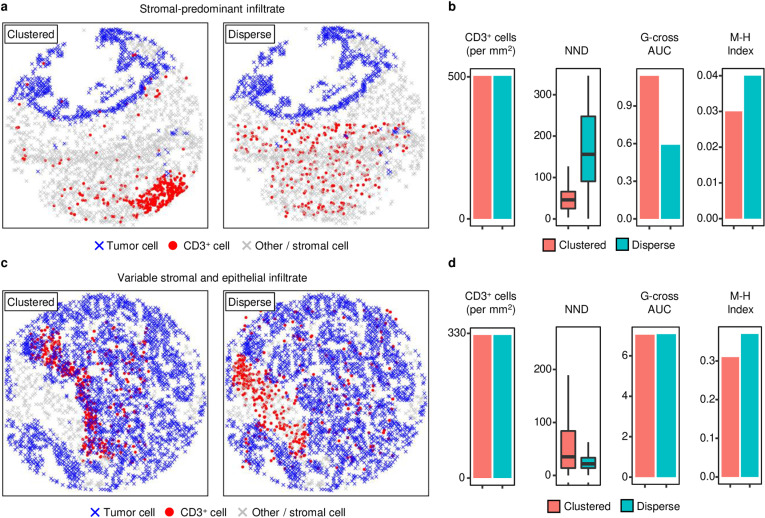
Demonstration of the limitations of existing spatial analysis methods, namely nearest neighbor distance (NND), G-cross function and Morisita-Horn index. Distinct spatial organization of CD3^+^ T cells in two representative tumor morphologies characterized by (a) stromal-predominant infiltrate, and (c) variable stromal and epithelial infiltrate, respectively, were simulated. In each tumor morphology, same amount of T cells either clustered within a small area (left panel, labelled as “Clustered”) or dispersed across the entire area (right panel, labelled as “Disperse”). In both simulated tumor morphologies (a, c), the two spatially distinct T-cell organizations demonstrate indistinguishably close (b, d) Morisita-Horn (M-H) indices and G-cross function AUCs. M-H index was computed based on a 5-by-5 µm rectangular grid; G-cross AUC was measured at r < 20 µm.

Several approaches from ecological studies and spatial point pattern analysis (SPPA; S1 Table) have been applied to analyze immune cell spatial organization within the TME [15–17]. These include direct nearest neighbor distance (NND) measurements as well as more sophisticated metrics such as the Morisita-Horn (M-H) index, an ecological measure adapted to quantify co-localization of immune and tumor cells based on subregion tessellation [18], and the G-cross [17] and L-cross SPPA functions [19]. These SPPA functions estimate a cumulative NND function or a neighborhood count function that reflects proximity between cell types. While these spatial measures have been used to identify tumor subtypes with prognostic associations, these methods have significant drawbacks including confounding by immune cell density, which often harbors independent prognostic significance, and variable tumor morphology, particularly with respect to degree of differentiation, which also has prognostic significance for many tumors. Additionally, by compressing an often-heterogeneous tumor-immune spatial relationship into a single numerical value, these approaches may discard key information. Examples of this information loss are provided (Fig 1), where tumors with identical morphology and immune cell densities harbor visually apparent differences in immune distribution that are not reliably captured by existing SPPA functions. More recently, an unsupervised approach by Schürch et al. [20] managed to delineate 9 distinct cellular neighborhoods in CRC using the CODEX technique and 56 markers. However, this computational approach is tailored for extensively profiled datasets containing a large number of markers and may not be applicable to the majority of immunofluorescence-stained datasets, particularly those generated in clinical settings.

To circumvent these limitations, we developed Tumor-Immune Partitioning and Clustering (TIPC), a novel computational algorithm designed for flexible exploration of spatial patterns in tumor-immune organization, independent of cell density and tumor morphology. As proof-of-principle, we applied TIPC to the prospective cohort incident tumor biobank method (PCIBM) [21,22] containing CRC tumor tissue specimens with spatially resolved, in-situ T-cell data in two large U.S.-wide prospective cohort studies. We extended our approach to neutrophils and eosinophils identified by histomorphology and machine learning [23], and subsequently validated the tumor-immune localization profiles using The Cancer Genome Atlas (TCGA) colon and rectal adenocarcinoma data. TIPC showed generally superior or comparable prognostic performance to existing spatial analysis methods, including cellular density, G-cross and L-cross functions, and the M-H index. Furthermore, we demonstrated that TIPC was instrumental in identifying critical cell interactions like CXCL9-CXCR3 and CXCL9-CD8 in hepatocellular carcinoma.

## Results

**Fig 2:**
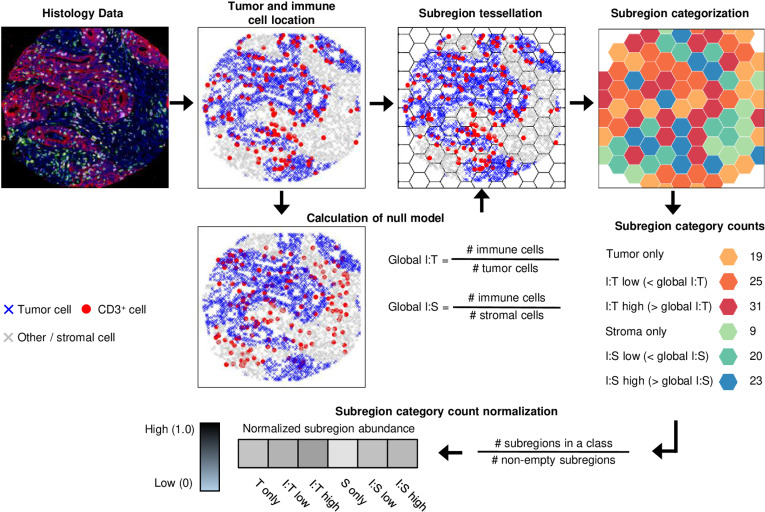
Implementation of TIPC, a computational method utilizing hexagonal tessellation and a classifier that evaluates multiple spatial parameters against a tumor region-specific null model represented by two global ratios based on the total number of immune (user-selected cell type of interest), tumor (global I:T) and stromal cells (global I:S). Using the Cartesian coordinates of these cells, TIPC divides the space into a hexagonal grid of subregions of specified subregion size and calculates two local ratios namely I:T and I:S for each subregion. The subregions are then classified into six different categories based on comparing the local to the global I:T and I:S ratios. In this mIF-stained image example, there were 19 “Tumor only” subregions containing only tumor cells; 25 “I:T low” subregions with a local I:T ratio less than the global I:T ratio; and 31 “I:T high” subregions with a local I:T ratio greater than the global I:T ratio. The three stromal categories were counted in a similar way. The number of subregions in each category are then normalized using the total number of subregions containing cells of any type. The resultant six-element numerical vector encodes the tumor-immune spatial organization of the TME for an ROI. Abbreviations: I:T, immune-to-tumor, I:S, immune-to-stroma.

TIPC requires a dataset specifying the location of individual tumor cells and an immune cell type of interest using cartesian coordinates within a region of interest (ROI). A hexagonal tessellation approach is applied to the ROI, and the number of tumor cells and immune cells within each hexagon subregion is calculated (Fig 2). By comparing the cellular composition of each hexagon to a global null distribution representing a uniform distribution of immune cells, TIPC simultaneously measures the degree of immune cell partitioning between tumor epithelial and stromal areas as well as the degree of immune cell clustering versus dispersion. These data are summarized as a six-element numerical vector for each ROI (S2 Table). This approach enables straightforward comparison between tumors and provides data that is readily amenable to spatial pattern discovery using unsupervised clustering approaches.

### Characterization of CD3^+^ T-cell organization patterns using TIPC

Case-level analysis using TIPC only requires selection of an immune cell of interest and a granularity or subregion size parameter. Selection of the optimal subregion size requires balancing the degree of resolution for immune cell partitioning between tumor epithelial and stromal areas against the probability of generating an excess number of uninformative subregions lacking immune cells (S1 Fig). The optimal subregion size may therefore vary based upon the distribution of the immune cell type being studied. For CD3^+^ T cells in CRC, evaluation of normalized counts across a range of subregion sizes showed that hexagons with a length of 70–80 pixels (35–40 μm) provided the most balanced distribution across the six TIPC subregion types (S1 Fig).

**Fig 3:**
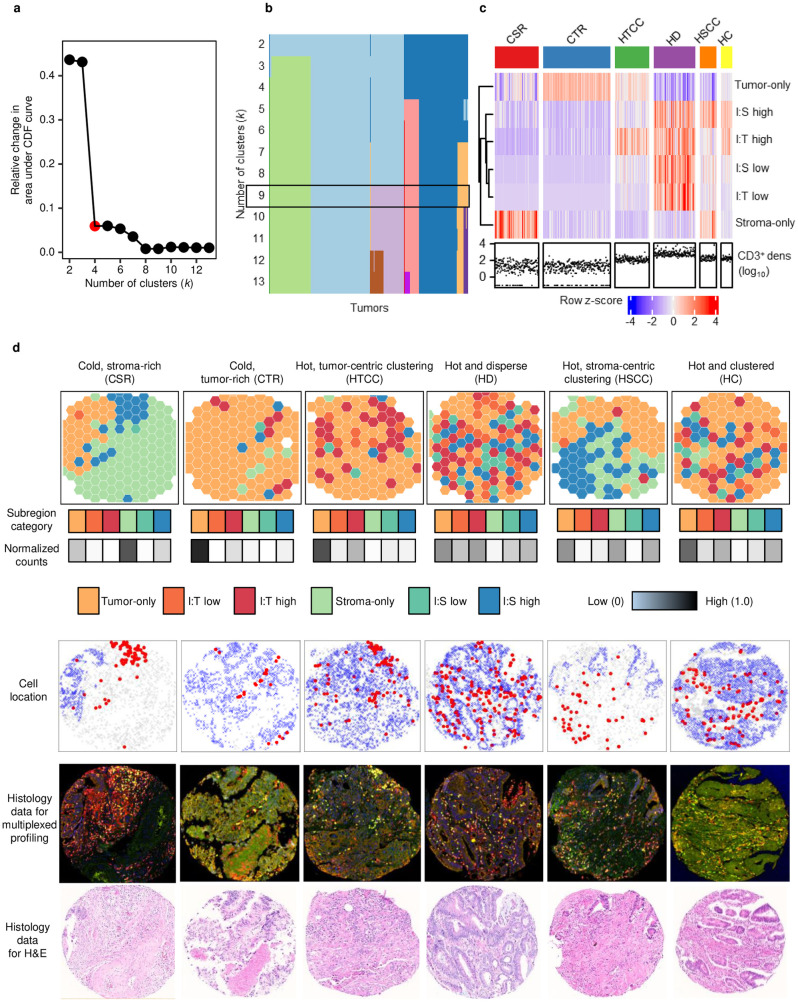
Characterization of CD3^+^ T-cell spatial distribution in the CRC tumor microenvironment using TIPC. Using the optimal subregion size of 35 µm and input cluster number of 9 (which was jointly determined using (a) the consensus cumulative distribution function (CDF) delta plot (k ≥ 4) and (b) tracking plot (k = 9), see S2 Fig for details), the resulting TIPC tumor subtypes and their spatial patterns are represented in (c) a heat-map with corresponding CD3^+^ T-cell density; subtypes comprising <30 tumors were excluded. (d) Representative cases with similar CD3^+^ T-cell densities (all within the 3rd quartile) were selected from each of the six main TIPC subtype clusters to illustrate the distinct spatial organization of CD3^+^ T cells in CRC. From top to bottom, the panels show TIPC subregion categories, cell locations, multiplexed immunofluorescence-based histology, and H&E-stained histology of adjacent slides. TIPC spatial parameter values are depicted on a linear scale showing ordered from left to right: tumor-only, I:T low, I:T high, stroma-only, I:S low, I:S high subregion categories. Abbreviations: I:T = immune-to-tumor, I:S = immune-to-stroma, CSR = cold, stroma-rich, CTR = cold, tumor-rich, HTCC = hot, tumor-centric clustering, HD = hot and disperse, HSCC = hot, stroma-centric clustering, HC = hot and clustered, and O = outliers.

To perform spatial pattern discovery using unsupervised clustering across a cohort, an optimal cluster number must also be determined. We assessed three contiguous subregion sizes of 30, 35, and 40 μm, and observed a reduced granularity at 40 μm, as detailed below. First, the relative change in the area under the cumulative distribution function (CDF) curve showed that stable clustering could be achieved using a minimum of seven, four, or seven clusters, respectively (Figs 3a, S2a, S2d, and S2g). We next constructed tracking plots to determine the maximum number of clusters that would enhance granularity while maintaining an adequate sample size (Figs 3b, S2b, S2e, and S2h). To balance statistical power with granularity, we selected 10-cluster, 9-cluster, and 7-cluster solutions, respectively, which yielded 6, 6, and 5 major clusters each comprising at least 30 tumors (S2c, S2f and S2i Fig). Visual inspection of representative tumors from these clusters revealed discrete patterns of tumor-immune cell organization. Six similar patterns were obtained with subregion sizes of 30 and 35 μm (Figs 3c, S2c and S2f). Tumors with abundant T cells comprised four clusters. One cluster, termed “hot and disperse”, contained tumors with a disperse distribution of immune cells across tumor and stromal regions. Three other clusters containing tumors with abundant immune cells were characterized by significant immune cell clustering as opposed to a disperse distribution and could be distinguished based upon whether immune cells clustered in tumor intraepithelial regions (“hot, tumor-centric clustering”), stromal regions (“hot, stroma-centric clustering”) or both regions (“hot and clustered”). Whereas tumors with uniformly few T cells fell into two clusters distinguished by whether tumor regions or stromal regions were predominant and accordingly designated “cold, tumor-rich” or “cold, stroma-rich”. As “hot and clustered” was undetected at subregion size of 40 μm (S2i Fig), we considered subregion sizes of 30 and 35 μm optimal, we used the latter (where 927 of 931 tumors fell within six clusters) for downstream association analyses (Fig 3c and 3d).

### Prognostic significance for distinct CD3^+^ T-cell patterns identified by TIPC

**Fig 4:**
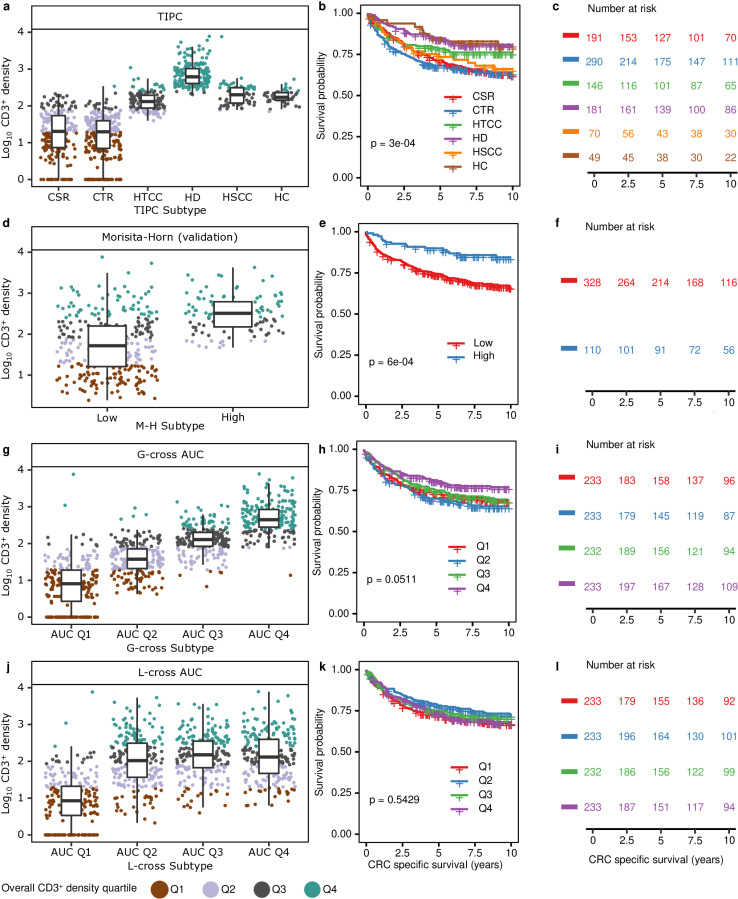
Comparison of TIPC performance with existing analysis methods, using CD3^+^ T-cell data. Tumor subtypes were identified using (a-c) TIPC, (d-f) Morisita-Horn (M-H) tumor cell:CD3+, (g-i) G-cross tumor cell:CD3+, and (j-l) L-cross tumor cell:CD3+. Box plots show that (a) TIPC subtypes were less confounded by the overall CD3+ T-cell density as compared to (d,g,j) other methods. Kaplan-Meier and log-rank test show (b,c,e,f,h,i) subtyped derived by TIPC, M-H, and G-cross harbored significant associations with colorectal cancer-specific survival, but otherwise for (k,l) L-cross method. G-cross and L-cross AUC quartiles were measured using radius of 20 µm based on stromal CD3^+^ cells (S7 and S8 Figs); M-H index was calculated using a 5-by-5 µm rectangular grid and 80th percentile dichotomization cut-off (S5 Fig); TIPC subtypes were obtained at the optimal subregion size of 35 µm and input number of clusters of 9 (S2 Fig). Abbreviations: CSR = Cold, stroma-rich; CTR = Cold, tumor-rich; HTCC = Hot, tumor-centric clustering; HD = Host and disperse; HSCC = Hot, stroma-centric clustering; HC = Hot and clustered.

**Fig 5:**
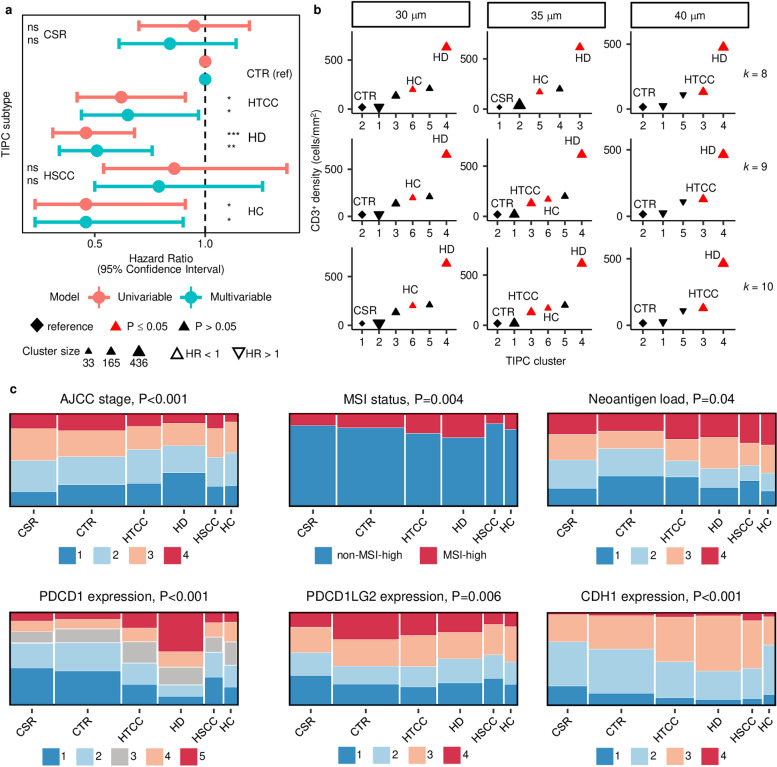
Prognostic and molecular associations TIPC subtypes identified using CD3^+^ T-cells in CRC. (a) Forest plots show the hazard ratios and confidence intervals determined using univariate and multivariate Cox regression models; symbols *** p < 0.001, ** p < 0.01, * p < 0.05, not significant (ns) p > 0.05. (b) Performance evaluation on the effect of subregion sizes and cluster number (k) on spatial subtype identification and prognostic significance, based on univariate Cox PH regression model (see S17 Fig for full data). (c) Stacked bar plots show the enrichment of clinicopathological features within individual TIPC subtypes, where extended Cochran–Armitage test was used to test the association significance. Abbreviations: CSR = cold, stroma-rich, CTR = cold, tumor-rich, HTCC = hot, tumor-centric clustering, HD = hot and disperse, HSCC = hot, stroma-centric clustering, and HC = hot and clustered. TIPC subtypes shown in (a,c) were obtained using the optimal subregion size of 35µm and input number of clusters of 9 (see S2 Fig).

Given that T cells generally need to be near tumor cells to exert their anti-tumor effect, we hypothesized that different TIPC clusters represent anti-tumor immune responses with varying degrees of effectiveness and may therefore harbor prognostic significance. Using the “cold, tumor-rich” cluster as the reference, the “hot and disperse”, “hot and clustered”, and “hot, tumor-centric clustering” clusters demonstrated longer CRC-specific survival in both univariable and multivariable analyses (Figs 4a–c, 5a and S3). Notably, while the “hot and disperse” cluster had a median T-cell density 3.6-fold higher than the “hot and clustered” cluster (Fig 4a), both clusters exhibited similar survival associations with no significant differences between these two subtypes for 5-year CRC-specific survival (p>0.05) (Fig 4b and 4c).

### Comparing TIPC with existing methods using CD3^+^ T-cell data

Besides showing that the direct NND measure did not harbor any prognostic significance (S4 Fig), we also compared the performance of TIPC with multiple methods that have been previously used to measure immune cell spatial distributions. M-H index was used to quantify the degree of co-localization between T cells and tumor (or stromal) cells. Overall, very few combinations of grid sizes and co-localization cut-offs yielded significant and robust associations with CRC-specific survival (S5 Fig). Examination of the overall performance of the M-H index did not reveal clear trends in significance aside from a decrease in false discovery rate (FDR) at higher index values. While the prognostic significance of tumor subtypes identified by the M-H index was confounded by T-cell density (Figs 4d and S6), prognostic utility could be improved by adjustment for T-cell density (S3 Table).

A SPPA metric, namely G-cross function, evaluates the likelihood of a tumor cell having at least one T cell within a specified radius. Using a Cox regression model, we found that larger area under the curve (AUC) for the G-cross function was associated with better CRC-specific survival (Figs 4h–I and S7). While G-cross-defined subtypes were also significantly confounded by T-cell density (Figs 4g and S7), unlike the M-H index, they did not harbor prognostic significance after adjustment for T-cell density in a multivariable Cox regression model (S3 Table). A related measure, the L-cross function, measures the expected number of T cells within a specified radius of a tumor cell. Analysis of L-cross AUCs did not identify prognostic significance (Figs 4k, 4l and S8) using all available L-cross estimators (isotropic, translation, and border). L-cross AUCs exhibited a lower correlation with T-cell density (Figs 4j and S8). In contrast to the M-H index and G- and L-cross functions, TIPC subtypes harbored prognostic significance that was minimally confounded by T-cell density (S4 Table). Moreover, multivariable Cox regression analyses suggested that TIPC subtypes are more robust than tumor subtypes identified by the M-H index and G- and L-cross functions (S4 Table) in that they are less confounded by other clinicopathologic features and remain stable when hexagon sizes and cluster number vary (Fig 5a and 5b).

### Clinicopathologic correlates of TIPC subtypes for CD3^+^ T cell

Beyond prognostic utility, correlating tumor-immune cell spatial distributions with pathologic and molecular features may help to refine tumor subtypes and our understanding of tumor biology, potentially improving treatment decisions. We found that TIPC tumor subtypes were associated with multiple histologic and molecular features including qualitatively assessed lymphocytic reaction patterns, American Joint Committee on Cancer (AJCC) stage, and microsatellite instability (MSI)-high status (Figs 5c and S9). While tumors in both the “hot and disperse” and “hot, tumor-centric clustering” clusters showed similar associations with MSI-high status, these tumors differed in T-cell spatial distribution with a 4.7-fold difference in median T-cell density, suggesting the ability of TIPC to uncover previously underrecognized positional patterns amongst highly immunogenic MSI-high tumors. TIPC subtypes also showed a strong association with the level of tumor-infiltrating PDCD1 (PD-1)^+^ cells and tumor PDCD1LG2 (PD-L2) expression, suggesting immune checkpoint-related mechanisms may be involved in sculpting T-cell spatial distributions. Finally, differential tumor expression of CDH1 (E-cadherin), an adhesion junction protein that is critical for maintaining epithelial cell-cell contacts, was observed between tumor subtypes with nearly the same T-cell density but different spatial patterns, raising the possibility that physical interactions between tumor cells, which manifest as tumor morphology, may shape tumor-immune spatial interactions.

### Cytotoxic memory T-cell spatial characterization using TIPC

**Fig 6:**
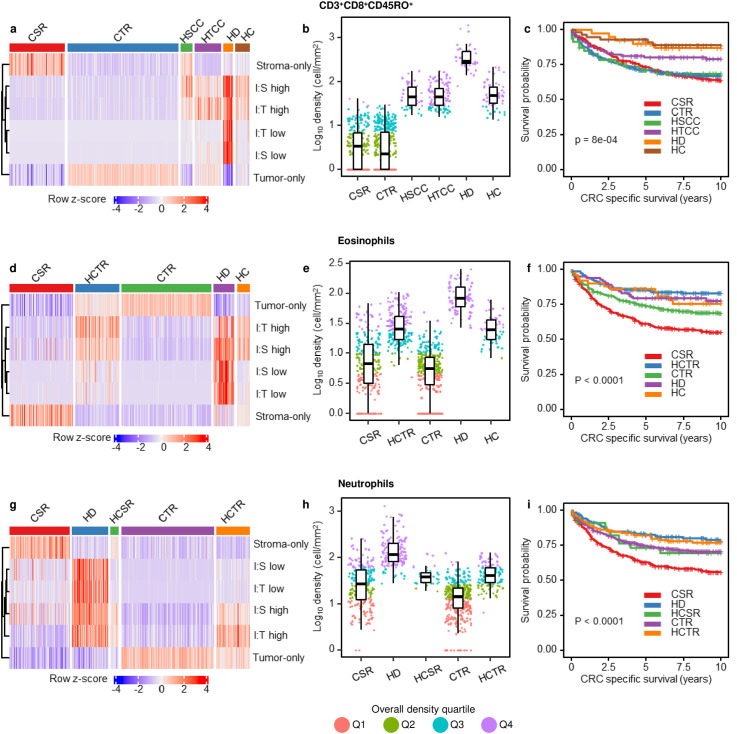
TIPC application on three different immune cell types, namely (a-c) cytotoxic memory T cells, (d-f) eosinophils, and (g-i) neutrophils in CRC (NHS/ HPFS). (a,d,g) Heat maps display the distinct immune cell organization of the resulting TIPC subtypes which demonstrate (b,e,h) variable but overlapping immune cell densities. (c,f,i) Kaplan-Meier and log-rank test show that these TIPC spatial subtypes were significantly associated with CRC-specific survival (see S3 Fig for the corresponding risk tables). Abbreviations, CSR = cold, stroma-rich, CTR = cold, tumor-rich, HD = hot and disperse, HTCC = hot, tumor-centric clustering, HSCC = hot, stroma-centric clustering, HC = hot and clustered, HCTR = hot and clustered, tumor-rich, HCSR = hot and clustered, stroma-rich.

T cells in the CRC TME comprise numerous, functionally distinct subsets. By incorporating additional markers profiled in our multiplex immunofluorescence assay, we used TIPC to explore the geographic placement of cytotoxic memory T cells (CD3^+^CD8^+^CD45RO^+^), one of the most biologically important T-cell subsets. This analysis grouped the 930 tumors into six subtypes (n=47–451 per subtype) with varying degree of immune cell infiltration, predominance of tumor or stromal regions and degree of immune cell clustering or dispersion (Fig 6a). These clusters exhibited variable yet overlapping cytotoxic memory T-cell densities (Fig 6b). Unlike the TIPC subtypes of CD3^+^ T cells, density scores can identify different subtypes within each TIPC cytotoxic memory T-cell subtype. Survival analysis showed that cytotoxic memory T-cell spatial distribution was associated with 10-year CRC-specific survival (Fig 6c). Using the “cold, tumor-rich” subtype as a reference, univariable Cox regression analysis revealed better CRC-specific survival for the “hot, tumor-centric clustering”, “hot and disperse”, and “hot and clustered” subtypes (S10–S12 Figs). However, these TIPC subtypes did not provide additional prognostic value to the cell density (S11 and S12 Figs). This suggests that both cell density and geographic placement of cytotoxic memory T cells may play equally important roles in anti-tumor response.

### Validation of TIPC using morphologically identified eosinophils and neutrophils

Although multiplex immunofluorescence enables highly accurate identification of many immune cell types, cost and assay complexity limit the widespread adoption of this approach. Our previous studies have shown that morphologically distinct immune cells, such as eosinophils and neutrophils, can be identified using routine hematoxylin & eosin (H&E)-stained CRC tissue sections and machine learning at an accuracy comparable to that of a gastrointestinal pathologist [23]. The significance of different localization patterns for myeloid cells in the TME is even less well understood than that for T cells. We applied TIPC to explore whether eosinophils and neutrophils exhibit distinct spatial distributions in the CRC microenvironment. Using TIPC to evaluate eosinophils or neutrophils both divided tumors into five major subtypes containing 34–362 tumors per subtype (Figs 6d, 6g, and S13–S16). Both analyses showed that TIPC could identify distinct distributions of neutrophils and eosinophils in tumors with overlapping immune cell densities (Fig 6e and 6h). Tumors with low neutrophil or eosinophil densities that fell into two TIPC subtypes, “cold, stroma-rich” and “cold, tumor-rich”, exhibited differential associations with CRC-specific survival (Figs 6f, 6i, and S10). Using the “cold, stroma-rich” subtype as the reference, these analyses revealed that tumor subtypes with largely overlapping cell density distributions, including “hot and clustered, tumor-rich” and “cold, tumor-rich” (Fig 6e and 6h), exhibited significantly better CRC-specific survival even after adjusting for neutrophil or eosinophil density (S11 Fig and S5 Table). In addition, the “hot and disperse” tumors exhibiting relatively high neutrophil or eosinophil densities were associated with better CRC-specific survival, even after adjusting for immune cell density (S5 Table) or clinicopathologic features (S11 Fig) in multivariable Cox regression model. For neutrophils, this subtype remained significant after joint adjustment for both immune cell density and clinicopathologic features (S12 Fig), highlighting the prognostic value of neutrophil spatial distributions.

### Evaluation of TIPC result stability

Modification of subregion size and cluster number can impact the spatial subtypes identified by TIPC. To investigate clustering solution robustness and stability, we combinatorially tested a broad range of subregion sizes (hexagon side length 30–50 μm, equivalent to 2301–6392 μm^2^) and input cluster numbers [4–10] in the CD3^+^ T-cell and H&E neutrophil datasets (Figs 5b, S17, and S18). Our interpretation was based on major clusters which comprised more than 30 tumors. In both analyses, when compared to the TIPC subtype with the lowest median immune cell density, the “hot and disperse” subtype maintained prognostic significance across all 35 tested combinations. Additionally, the “cold, tumor-rich” subtype exhibited equally broad and robust prognostic significance for neutrophil analysis. Less broad, yet still robust associations were seen for the “hot, tumor-centric clustering” subtype identified using CD3^+^ T cells and the “hot and clustered, tumor-rich” subtype identified using neutrophils.

In general, a more granular characterization of the immune cell spatial landscape was achieved using a smaller subregion size and larger cluster number. In this study, we excluded clusters comprising less than 3% (i.e., 30) of the total number of tumors for downstream association analysis to prevent unstable subtypes. Across the tested immune cell types, subregion sizes in the range of 35–45 μm with at least 6 input clusters demonstrated robust TIPC solutions which contained consistent spatial subtypes with prognostic significance.

In addition to demonstrating the stability of TIPC analysis, we provide heuristic-based approaches to further simplify analysis and minimize user intervention. These computational methods include variance minimization to identify the most informative subregion size, shoulder point detection to determine stable cluster numbers, and tracking the consistency of normalized mutual information at increasing *k* to select granular yet stable cluster numbers (S19 Fig). Using the heuristic algorithms, we obtained spatial subtypes with characteristics similar to those achieved through careful selection of subregion size and cluster numbers. Notably, in the CD3^+^ T-cell analysis, the subregion size and cluster numbers determined by these algorithms matched those based on manual selection, and the eosinophil and neutrophil analyses produced identical spatial subtypes, despite using different subregion sizes (S19d–I Fig). However, in the cytotoxic memory T-cell analysis, the heuristic approach resulted in an additional subtype characterized by both ‘cold, tumor-rich’ and ‘cold, stroma-rich’ regions (S19a–c Fig). These two subtypes of cytotoxic memory T-cells did not differ in cell density (Fig 6b) or prognosis (S11a and S12a Figs).

### Extrapolation of TIPC spatial subtypes in The Cancer Genome Atlas (TCGA)

**Fig 7:**
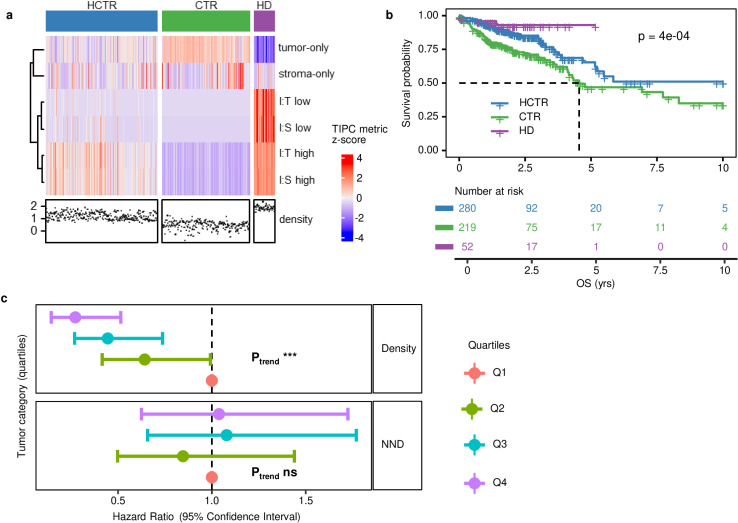
Validation of TIPC spatial subtypes which were first determined in NHS/ HPFS cohort and later recapitulated in TCGA cohort, using eosinophils identified morphologically in H&E images. (a) Among the five major TIPC subtypes determined in NHS/ HPFS cohort, three comprised of >30 tumors (i.e., CTR, HD, HCTR). (b) Kaplan-Meier estimates associated with these TIPC subtypes of eosinophils harbored significant association with overall survival (OS); (c) forest plot summarizes Cox regression analysis of tumor subtypes determined using cell density or nearest neighbor distance (NND) (see S19 Fig for the associations with disease-specific survival and progression-free intervals). Symbols *** p < 0.001, ** p < 0.01, * p < 0.05, not significant (ns) p > 0.05. Abbreviations, CTR = cold, tumor-rich, HD = hot and disperse, HCTR = hot and clustered, tumor-rich.

We hypothesized that the heterogeneous, yet unique anti-tumor immune response captured by TIPC subtypes would preserve in the same tumor type (in this case, CRC), and thus patient stratification paradigm constructed using TIPC and the associated prognostic utility obtained in our cohorts (i.e., Nurses’ Health Study (NHS) and Health Professionals Follow-up Study (HPFS) CRC datasets [24,25]) could be extended to unseen CRC tumors in other cohorts. Given the suboptimal nature of some of the H&E images in TCGA, the validation analysis focused only on eosinophils which can be morphologically identified from H&E images with high confidence. Using eosinophils identified from the H&E images of 571 CRC tumors from TCGA, we assigned each of these tumors to the most similar TIPC subtype found in our cohorts using k-nearest neighbor (kNN) method, resulting in three major subtypes containing 52–280 tumors per subtype (Fig 7a). We noticed that both the “cold, stroma-rich” and “hot and clustered” subtypes were under-represented in TCGA cohort, comprising of only 11 and 8 tumors, respectively (Fig 7a).

In our cohorts, based on the Kaplan-Meier estimates, the “cold, stroma-rich” subtype harbored significantly worse CRC-specific survival (Fig 6f and 6i) in eosinophils, while other subtypes showed largely similar prognosis except for the “Cold, tumor-rich” (Fig 6f). Such prognostic associations can be validated in TCGA cohort (Figs 7b and S20) that, with the “cold, stroma-rich” subtype absent, “cold, tumor-rich” subtype still harbored significantly worse prognosis in eosinophils (Figs 7b and S20a–b). Conversely, density- and NND-based survival analyses (Figs 7c and S20c) were unable to resolve such granular information.

Above we have showed that, among existing methods under studied, only M-H index (also a tessellation-based method) harbored prognostic value beyond CD3^+^ cell densities (S3 Table). To validate the prognostic utility of M-H index, we first determined the optimal grid sizes and co-localization cut-offs using the eosinophils identified in our cohorts. The data showed that none of the tested combinations on eosinophils harbored a significant association (S21 Fig). When applying to TCGA cohort, these cut-off values resulted in extremely skewed distributions of tumor subtypes (M-H high and low groups) (S22 Fig).

### Evaluation of the significance of CXCL9-CXCR3 axis in hepatocellular carcinoma using TIPC

**Fig 8:**
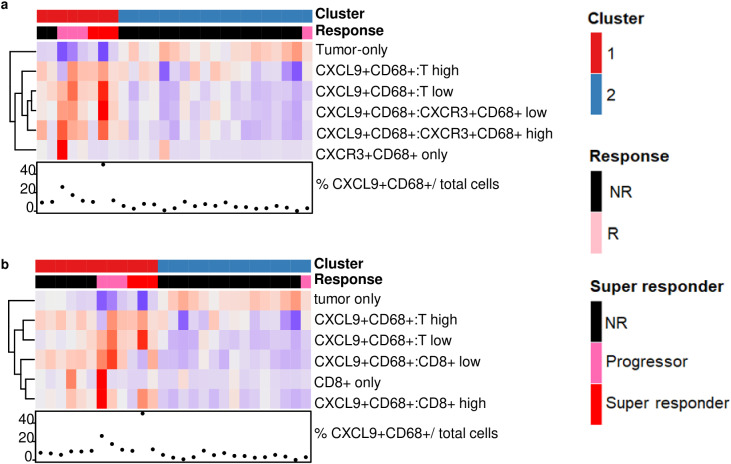
Using TIPC to discern spatial patterns of immune activity in an HCC cohort and their correlation with patient treatment response. (a) Responders (Rs) exhibited a unique spatial pattern of CXCL9^+^/CXCR3^+^ CD68^+^ macrophages: a uniform distribution of CXCL9^+^CD68^+^ cells within tumor regions, along with the co-existence of CXCL9^+^CD68^+^ and CXCR3^+^CD68^+^ cells, identified as TIPC cluster 1, where CXCR3^+^CD68^+^ cells replaced stromal cells in the TIPC analysis. (b) Patients enriched with tumor areas deficient in CXCL9^+^CD68^+^ consistently correlate with adverse outcomes, identified as TIPC cluster 2. Additionally, Rs exhibited concurrent enrichment of CXCL9^+^CD68^+^ in both tumors and CD8^+^ regions, where CD8^+^ T cells replaced stromal cells in the TIPC analysis. Both sets of TIPC subtypes were obtained at the optimal subregion size of 30 µm and input number of clusters of 2, determined based on the most significant p values. Tumor cells were determined as those no expressing CD8, CD68, and CD45 expression.

We extended the application of TIPC to assess how the spatial distributions of immune activity markers correlate with the response of hepatocellular carcinoma (HCC) to a combination of Y90-radioembolization and nivolumab treatment [26]. We focused on the organizational patterns of two phenotypes, CXCL9^+^CD68^+^ and CXCR3^+^CD68^+^; the latter replaced stromal cells in the TIPC analysis. A previous study assessing independently each phenotype within the same subjects demonstrated their positive correlation with patient responses [27]. Our TIPC analysis revealed that responders were characterized by a unique spatial pattern, including a uniform distribution of CXCL9^+^CD68^+^ cells within tumor regions and the simultaneous presence of CXCL9^+^CD68^+^ and CXCR3^+^CD68^+^ cells, identified as TIPC cluster 1 (Fig 8a). Given the critical role of CXCL9 as a T-cell attractant, effective anti-tumor response of CXCL9+CD68+ cells may necessitate their proximity to T cells. Hence, we examined the localization relationship between CXCL9^+^CD68^+^ and CD8^+^ T cells within the HCC TME, where CD8^+^ T cells replaced stromal cells in the TIPC analysis. Our findings indicate that tumor lacking CXCL9^+^CD68^+^ were consistently correlated with adverse outcomes, categorizing these non-responders as TIPC cluster 2 (Fig 8b). All CXCL9^+^CD68^+^–associated TIPC metrics were significantly linked to response status, with p-values < 0.05 using the Kruskal-Wallis test. Conversely, responders exhibited a concurrent enrichment of CXCL9^+^CD68^+^ across both tumor and CD8^+^ regions (as shown in cluster 1 of Fig 8b).

## Discussion

Recent advances in digital imaging and multiplexed marker analysis by immunofluorescence have enabled deep phenotyping of immune cells in the TME. However, the optimal methods to analyze these data remain uncertain. Immune cell density is an easily defined and computed characteristic of immune cells in the TME and has proven prognostic significance in many cancers [5,8,9]. However, increasing evidence suggests that immune cells are not randomly scattered throughout the TME but are instead arranged in patterns that likely reflect their biological function and interactions with numerous cell types, including tumor cells, stromal cells, and other immune cells [20,28]. Identification and quantification of these patterns may therefore provide additional information about the significance and function of immune cells in a tumor beyond density measurements.

To address this need, we developed a novel computational algorithm TIPC. Application of TIPC to multiplex immunofluorescence data derived from a large cohort of CRC demonstrated that discrete patterns of immune cell infiltration exist within the center regions of CRC. These patterns can be distinguished based on the relative enrichment of immune cells in stromal versus epithelial regions and the degree of clustering versus dispersion of immune cells in the TME. Of the six TIPC subtypes identified by CD3^+^ cell analysis, only the “hot and disperse” subtype could have been identified solely using CD3^+^ cell density. Evaluation of the prognostic significance of TIPC subtypes defined by CD3^+^ cells revealed that three TIPC subtypes exhibited better CRC-specific survival when compared to the “cold, tumor-rich” subtype. Within these three subtypes associated with better outcomes, CD3^+^ cell densities differed more than 3.5-fold, indicating that immune spatial configuration and not just density harbors prognostic significance. While the prognostic significance of TIPC subtypes was partially attenuated when CD3^+^ density itself was included in a multivariable Cox regression analysis, two subtypes remained significant. Comparison of TIPC to other approaches showed that TIPC achieves equal or superior prognostic power. Furthermore, by representing the immune cell localization patterns of each tumor as a six-element vector rather than compressing this information into a single aggregate value, as done by other algorithms, TIPC can capture and convey more information about any given immune cell distribution. Finally, since TIPC does not rely upon direct distance measurements between tumor and immune cells, it is likely to be less confounded by differences in tumor morphology. In contrast, SPPA functions such as G-cross and L-cross treat every tumor cell as an isolated entity without recognizing that tumor cells themselves are non-randomly distributed and often exist in clustered glandular units [29,30].

Beyond harboring prognostic significance, TIPC subtypes were associated with numerous histologic and molecular features. Strong, yet heterogeneous associations with the four qualitative lymphocytic reaction patterns as judged by H&E-based whole-slide analysis support the notion that lymphocyte distribution at the tumor-wide level is linked to smaller-scale lymphocyte distribution as assessed by TIPC. While several TIPC subtypes were significantly associated with MSI status, no individual subtype was associated with neoantigen load. Given that high level microsatellite instability and high neoantigen loads are both associated with higher T-cell density, the relatively modest overall associations we observe with TIPC subtypes suggest that immune cell spatial distributions are shaped by factors beyond mutations in tumor cells. In support of this concept, markers of multiple biologic processes known to be central to CRC biology, including cell adhesion (CDH1), WNT signaling (CTNNB1), prostaglandin signaling (PTGER2), and autophagy (SQSTM1), were more strongly associated with TIPC subtypes. Beyond tumor cell-intrinsic markers, we found that expression of PDCD1 (PD-1) by non-tumor cells was highly associated with multiple TIPC subtypes, hinting at regulation of immune cell spatial distributions by CD274 (PD-L1)/ PDCD1 (PD-1) immune checkpoint signaling between different immune cell populations.

Our study further establishes that TIPC is effective in revealing significant distribution relationships in different tumor types. Despite the limited size of our HCC cohort, TIPC successfully identified key spatial interactions, thereby validating the anticipated importance of the CXCL9-CXCR3 axis in HCC patient responses to the combined treatment of Y90-radioembolization and nivolumab. This confirmation stems from the observed cell density correlations of CXCL9^+^CD68^+^ and CXCR3^+^CD68^+^, analyzed individually [27]. Moreover, it highlighted the importance of the presence of CXCL9^+^CD68^+^ throughout tumor regions and their proximity to CD8^+^ T cells. These insights not only elucidate the mechanisms underlying the anti-tumor immune response but also help to clarify previous findings regarding the seemingly minimal involvement of T cells in this HCC treatment approach, which may have been previously underappreciated due to analyses focusing solely on cell density without considering cellular interactions [27].

Many different immune cell populations are present in the TME and can be detected using morphology, immunohistochemistry or multiplex immunofluorescence. We therefore designed TIPC to function independently of immune cell identification method and confirmed prognostically informative application of TIPC to datasets comprising T lymphocytes detected by a single marker (CD3), cytotoxic memory T cells detected by a triple marker combination (CD3^+^CD8^+^CD45RO^+^) and neutrophils and eosinophils detected by morphology alone. Importantly, the prognostic significance of TIPC subtypes identified in our cohorts can be recapitulated in an independent TCGA CRC cohort with prognostic value. Additionally, the data requirements to run TIPC are modest and encompass only the locations of the tumor cells and an immune cell type of interest in a format that is readily generated by many digital image analysis software systems. TIPC can also be used to jointly analyze a cohort of cases to identify subtypes with shared patterns of immune infiltration or it can be run at the single-case level to generate interpretable spatial immune characteristics. This combination of requirements, along with low computational overhead, position TIPC as an algorithm that could be readily implemented as part of a routine H&E-based digital workflow in a clinical surgical pathology environment. Finally, TIPC incorporates methods that enable investigation of the stability and robustness of solutions relating to subregion tessellation size and cluster number for cohort analyses, thereby facilitating the identification of optimal parameters. Our data showed that subregion sizes in the range of 35–45 μm with at least 6 initial clusters would be optimal for CRC study.

Our study has several limitations. First, due to the unavailability of treatment information, we are unable to account for potential confounding in outcome analyses. However, it is unlikely that treatment decisions were influenced by immune cell positional configuration, as tumor-immune spatial organization information was most likely unavailable to oncologists at the time of treatment decision-making and do not play any current role in therapeutic decision making. Second, the unsupervised hierarchical clustering in TIPC, like other clustering methods, requires a sufficiently large sample size to robustly identify spatial subtypes with prognostic and biological relevance. Despite this, TIPC successfully identified spatial subtypes linked to treatment response in a 27-sample HCC cohort. Thirdly, although the CXCL9-CXCR3 axis is known to play a role in recruiting immune cells, specifically cytotoxic T lymphocytes and macrophages [31], the current multiplexed panel design for the HCC cohort includes CD68 but lacks additional confirmatory macrophage markers. Therefore, we cannot formally exclude the possibility that some non-macrophage CD68+ cells contribute to the observed spatial relationships. Fourthly, selecting the two parameters—subregion tessellation size and cluster number—in TIPC analysis requires human interpretation and decision-making, guided by downstream association analyses such as survival and treatment response associations. To support this, the TIPC R package offers comprehensive visualization tools, including CDF, tracking, trend plots, and heatmaps. Additionally, heuristic approaches are available in the package to further simplify analysis and minimize user intervention.

Moreover, while we acknowledge that TIPC is more computationally intensive than conventional algorithms, we note that it provides more granular immune cell organization metrics and also enables users to readily examine a broad parameter space to uncover nuanced spatial patterns. Specifically, for the NHS/HPFS CRC cohort (N=931) with CD3^+^ T cells, on a local computer [Intel Core i7-10610U CPU (1.80 GHz base, 2.30 GHz max) with 16 GB of RAM], the M-H analysis took 0.12 hours across four tested grid sizes, while G-cross and L-cross each took 0.1 hours. In contrast, applying TIPC to the same CD3^+^ T cell dataset required approximately 5.6 hours on the local computer and 6 hours on the TIPC web server [Intel Xeon E5-2620 v4 processor (2.10 GHz) with 32 GB of RAM]. For the smaller HCC cohort (N=27), the analysis took about 0.75 hours locally and 0.5 hours on the web server over subregion sizes of 60 to 120 µm. Further improvements to TIPC’s efficiency, such as implementing multi-thread processing, would be beneficial. Additionally, TIPC is currently limited to analysis of three immune cell types at a time. TIPC is designed for flexible, robust microgeometric analysis, tailored to low/targeted-plex multispectral immunofluorescence assays found in clinical settings. It complements more intricate methods demanding extensive staining of lineage, cell state, and structural markers, as demonstrated by Schürch et al. [20]. Finally, our study was limited to tumor regions selected from the centers of CRCs, precluding evaluation of the prognostic significance and clinicopathologic correlates of immune cell spatial configuration at the invasive margin [12]. Further validation of TIPC using different cancer types and tissue sampling strategies is needed.

In conclusion, TIPC provides a novel approach for measuring immune cell localization configuration in the tumor immune microenvironment in a manner that is compatible with any tissue image data source that provides single-cell-level positional information. TIPC is specifically designed to measure immune cell partitioning and clustering, two features that are prognostically significant and biologically relevant, yet cannot be inferred from immune cell density alone. Additionally, this approach facilitates the exploration of associations between the organization features and treatment response, offering a deeper understanding of therapy efficacy. We therefore anticipate that application of TIPC to clinically available H&E, conventional immunohistochemistry, or multiplex immunofluorescence digital images will enable comprehensive characterization of the tumor-immune interactions that govern the anti-tumor immune response, ultimately leading to improvements in patient care driven by an improved understanding of tumor immunobiology and more refined patient stratification.

In summary, our TIPC algorithm represents a significant advancement in the microgeometric analysis of the tumor immune microenvironment. By leaping much beyond traditional proximity measurements to assess immune cell spatial configuration, TIPC enables a more nuanced understanding of tumor-immune cellular interplay. The application of TIPC to CRC has unveiled distinct positional patterns of immune cell infiltration, revealing that immune cell organizational arrangement is not only a marker of tumor phenotype but also a prognostic indicator. This approach, which correlates organization features with clinical outcomes, paves the way for more precise and personalized therapeutic strategies. The adaptability of TIPC to various imaging modalities, from H&E to advanced multiplex immunofluorescence, further enhances its clinical relevance, offering a practical tool for routine use in pathology laboratories. By providing deeper insights into the molecular mechanisms governing immune responses in cancer, TIPC holds the promise of refining therapeutic interventions and improving patient management, thereby contributing to the evolution of precision oncology towards more tailored and effective solutions.

## Methods

### Ethics Statement

The NHS/HPFS study protocol was approved by the institutional review boards of the Brigham and Women’s Hospital and Harvard T.H. Chan School of Public Health, under IRB reference number 2019P003588, and those of participating registries as required. Informed written consent was obtained from all of the subjects.The HCC study involves human participants and was approved by SingHealth Centralised Institutional Review Board (CIRB) under CIRB Reference Number 2018/3046. Participants gave informed written consent to participate in the study before taking part.

### Study design

We collect data from two prospective cohort studies in the U.S., the Nurses’ Health Study (NHS, 121,701 women aged 30–55 years followed since 1976) and the Health Professionals Follow-up Study (HPFS, 51,529 men aged 40–75 years followed since 1986) [32]. Every two years, study participants have been followed with questionnaires to collect information on lifestyle factors and medical history including CRC [24,25]. The National Death Index was used to ascertain deaths of study participants and identify unreported lethal CRC cases. Participating physicians reviewed medical records to confirm diagnoses of CRC, and to record data on tumor characteristics including anatomic location and disease stage based on the American Joint Committee on Cancer TNM (Tumor, Node, Metastasis) classification. Formalin-fixed paraffin-embedded tissue blocks were collected from hospitals where participants diagnosed with CRC had undergone tumor resection and used to invent the prospective cohort incident tumor biobank method (PCIBM) [21,22]. We included 931 patients with available CRC tissue microarray data diagnosed up to 2008 within the PCIBM. Our tissue microarrays included up to four cores from CRC and up to two cores from normal tissue blocks, as described previously [33]. We included both colon and rectal carcinomas, on the basis of the colorectal continuum model [34]. This study was approved by the institutional review boards at Harvard T.H. Chan School of Public Health and Brigham and Women’s Hospital (Boston, MA), and participating registries as required. Our main hypothesis is that spatial organization of immune cells in TME, as captured by TIPC, represent anti-tumor immune responses with varying degrees of effectiveness and may therefore harbor prognostic significance.

To validate TIPC-identified spatial subtypes and their prognostic utility, we included an independent cohort which is TCGA colorectal adenocarcinoma study [35]. We obtained the clinical elements and survival outcome data (including overall survival; disease-specific survival; progression-free intervals,) from the integrated TCGA Pan-Cancer Clinical Data Resource [36]. Among 616 cases with digitized H&E-stained histologic slides available in TCGA data portal, we excluded cases with unrepresentative images (image scanned below 20×magnification; image not showing primary CRC; image out of focus or obscured by slide markings), or cases with no follow-up data, resulting in 570 patients in the final analyses.

To explore the generalizability of TIPC on a different tumor type, we also included a small HCC cohort from a single-arm, single-center, two-stage phase 2 trial (CA 209-678). The trial was aimed at evaluating the efficacy and safety of Y90-radioembolization followed by nivolumab. A total of 36 patients with advanced HCC and Child-Pugh A cirrhosis were recruited at National Cancer Centre Singapore/ Singapore General Hospital, Singapore. Out of these, 27 patients who had pre-treatment biopsy tissues available were included in our study; among them, 20 were classified as non-responders and 7 as responders (including 4 progressors and 3 super responders), according to RECIST criteria [26]. Participants provided written informed consent [26].

### CRC T-cell multiplex immunofluorescence staining

Deparaffinized 4 µm sections from tissue microarray blocks were incubated with the primary antibody, then treated with anti-mouse/rabbit horseradish peroxidase conjugated (HRP) secondary antibody (Opal Polymer; PerkinElmer, Hopkinton, MA), and finally incubated with the fluorophore amplification reagent (PerkinElmer) for tyramide signal amplification. The slides were sequentially stained using the following antibodies/fluorescent dyes, in order: anti-CD3 antibody (clone F7.2.38; Dako; Agilent Technologies, Carpenteria, CA)/Opal-520, anti-FOXP3 (clone 206D, Biolegend, San Diego, CA)/Opal-540, anti-CD45RO (one isoform of PTPRC gene products) (clone UCHL1, Dako)/Opal-650, anti-CD8 (clone C8/144B, Dako)/Opal-570, anti-CD4 (clone 4B12, Dako)/Opal-690, anti-KRT (keratin, pan-cytokeratin)(clone AE1/AE3, Dako) in combination with anti-KRT (clone C11, Cell signaling, Danvers, MA)/Opal-620. Each slide was then treated with a nuclear counterstain 14’,6-diamidino-2-phenylindole (DAPI) (FP1490, PerkinElmer) [37].

Digital images were acquired using a Vectra 3.0 quantitative pathology imaging system (PerkinElmer) equipped with a 20× objective. Large areas with necrosis, artefact, or excessive tissue folding were excluded from analysis. Demultiplexed images of each tumor first underwent tissue segmentation to identify regions of tumor epithelium and peritumoral stroma based on cytokeratin expression using CRC-specific supervised machine learning algorithm executed within inForm 2.4.1 (PerkinElmer). Following tissue segmentation, cell enumeration was performed using the DAPI signal to define nuclei. Each cell was further segmented into nuclear, cytoplasmic, and membranous compartments. A separate supervised machine learning algorithm was used to identify T cells based upon a combination of cytomorphology and subcellular T-cell marker expression patterns. These single-cell data were then used to calculate T-cell subpopulation densities within separate regions. Aggregate tumor-level densities were then determined by calculating the average density for each T-cell subset across all cores from each tumor.

### HCC immune activity multiplex immunofluorescence staining

The multiplex immunofluorescence was conducted by following a similar Opal method as detailed above and previously described [27]. Briefly, deparaffinized/rehydrated formalin-fixed, paraffin-embedded tissue sections were treated with heat for epitope retrieval, followed by blocking of peroxidase activity. They were then incubated with primary antibodies targeting CD8 (clone 4B11, Leica), CD38 (clone SPC32(38C03), Leica), CD45 (PTPRC; clone 2B11+PD7/26), Agilent-Dako), CD68 (clone PG-M1, Agilent-Dako), CXCR3 (clone #49801, R&E Systems), and CXCL9 (clone 11H1L14, Invitrogen), followed by the application of polymeric horseradish peroxidase-conjugated secondary antibodies (Leica Biosystems, Newcastle-upon-Tyne, UK) and Opal tyramide signal amplification (TSA) reagents (Akoya Biosciences). After TSA deposition, the slides underwent heat-induced antibody stripping, with the labeling cycle repeated for each of the six markers before a final counterstain with spectral DAPI (Akoya Biosciences). Imaging was performed using the Vectra 3 pathology imaging system (Akoya Biosciences), and a pathologist (JY) examined the slides to identify multiple ROIs that exhibited high-quality staining and contained viable tumor cells. These ROIs were captured at 20 × magnification for subsequent analysis and scoring by the pathologist using inForm software (V.2.4.2; Akoya Biosciences) alongside HALO (Indica Lab, Albuquerque, New Mexico, USA).

### H&E-based eosinophil and neutrophil phenotyping

We used QuPath (v0.1.2), an open source software platform for whole-slide image analysis, to identify tumor cells, eosinophils and neutrophils in images of H&E-stained CRC tissue [38]. The H&E-stained sections were scanned using the brightfield mode of a Vectra 3.0 quantitative pathology imaging system (PerkinElmer) equipped with a 20× objective. The following image analysis steps were performed in a sequential fashion across all images: (1) estimate stain vectors to extract the H&E stain vectors and background values from the images; (2) simple tissue detection to discriminate the tissue region from the white background; (3) cell detection to detect cells based on the size, shape, and optical density of nuclei in the hematoxylin layer and to calculate features of the cells including nuclear area and circularity; (4) add s*moothed features* to calculate Gaussian-weighted means of the cell measurements in the neighboring cells; and (5) *create detection classifier* to train a random forests classifier using a 150 image subset to identify the cell types of interest.

### TIPC analytical approach

TIPC is a computational method that utilizes hexagonal tessellation and a classifier that evaluates multiple spatial parameters against a tumor region-specific null model representing a state of neutral tumor-immune cell interactions. Fig 2 depicts the key components of a TIPC analysis using an example multiplex immunofluorescence image. The TIPC R package is freely available on the web https://github.com/SIgN-CI/TIPC; the TIPC algorithm’s web application can be accessed at https://mspc.bii.a-star.edu.sg/minhn/tipc.html.

In a ROI, the locations of individual tumor cells, stromal cells and an immune cell type of interest need to be represented using Cartesian coordinates. As demonstrated in HCC analysis, a second immune cell type may replace stromal cells in analyses of immune cell-to-immune cell spatial associations. Using these input data, TIPC divides the ROI into a hexagonal grid of subregions (*spatstat* R package (v1.62-2) [39]) of the specified subregion size, and calculates two global ratios, i.e., total number of immune-to-total number of tumor cells and total number of immune-to-total number of stromal cells, together representing a state of neutral tumor-immune cell interactions which is the null model. The subregions are subsequently classified into six different categories, namely tumor-only, immune-to-tumor low (I:T low), immune-to-tumor high (I:T high), stroma-only, immune-to-stroma low (I:S low), and immune-to-stroma high (I:S high), based on comparing the immune, tumor and stromal cell content of each subregion to the global I:T and I:S ratios (S2 Table). If a subregion contains only tumor cells, it is categorized as tumor-only, whereas if any immune cells are found in that subregion is classified as I:T high or I:T low depending upon whether the I:T ratio of the subregion is greater or less than the global I:T ratio. The three stromal categories are defined in a similar way. The number of subregions in each category is then normalized using the total number of subregions containing cells of any type. The resultant six-element numerical vector (hereafter called TIPC spatial parameters) encodes the tumor-immune spatial organization of the TME for an ROI.

As a rule of thumb, an optimal subregion size balances the degree of resolution for immune cell partitioning between tumor epithelial and stromal areas against the probability of generating an excess of uninformative subregions that do not contain immune cells. For instance, an undersized subregion overlooks important cell interactions with an under-representation of immune-containing subregion categories including I:T low and high, and I:S low and high. In contrast, an oversized subregion generates spurious and noisy cell interactions with an over-representation of I:T low and I:S low subregion categories. To examine the effect of subregion size on positional measures, the TIPC R package incorporates three auxiliary functions. The *multiple_hexLen_tessellation* and *multiple_hexLen_count_TIPC_cat* functions perform hexagonal tessellation of the Cartesian space and calculate the six-element numerical vector at a range of subregion sizes (default: 30 to 45 μmat 5-μm intervals), respectively. The *trend_plot_hexLen* function generates a trend plot of the six TIPC positional measures as a function of subregion size. To further test the robustness and stability of TIPC solutions, the *multiple_hexLen_tessellation* and *multiple_hexLen_count_TIPC_cat* functions also measure five sets of the six-element TIPC spatial parameters by slightly shifting the hexagonal grid from the center (default) to the left, right, upper, and lower directions at a distance equal to half of the hexagon length. A trend plot of the size TIPC positional measures as a function of shifting direction can be generated by *trend_plot_shiftDirection*. Accessing multiple subregion sizes and involving shifted hexagonal grids helps address the effect of incompletely filled hexagonal subregions at the tissue border. While careful consideration should be given to selecting the optimal subregion size—informed by prior knowledge about the cohort and tumor type under study—the *optimal_hexLen* function in the TIPC package facilitates broader adoption. This function determines the optimal subregion size by minimizing the variance of the six TIPC spatial parameters to ensure informativeness, and by minimizing variance across the five directional shifts within each TIPC parameter to ensure robustness.

The six-element numerical vectors of multiple tumors can be readily compared, for instance using a Pearson correlation distance, enabling arrangement pattern discovery across a cohort. TIPC employs an unsupervised hierarchical clustering algorithm (*consensus_clustering* function from *ConsensusClusterPlus* R package) to group tumors with similar immune spatial organization as encoded by TIPC measures into unique subtypes. *consensus_clustering* involves several clustering parameters, (1) the distance function (default: Pearson’s correlation); (2) number of clusters k (default: 2 to 6); (3) number of repeated clustering for achieving a consensus grouping (default: 50); and (4) proportion of randomly selected cases to be included in each repeat (default: 80%). Specifically, a consensus clustering solution is obtained by repeating the clustering process for a specified number of times (default: 50) based on which a cumulative consensus matrix is determined. The cumulative consensus matrix is an n-by-n matrix (n: number of tumors) wherein the element ij represents the number of times tumor i is assigned to the same cluster as tumor j. The final clustering assignment is determined through applying a hierarchical agglomerative clustering with complete linkage to the cumulative consensus matrix. The clustering results are presented as a heatmap to facilitate inspection of clustering quality (for instance, identification of noisy patterns) as well as interpretation of tumor-immune spatial patterns (See S6 Table).

To identify stable and robust distribution patterns from a cohort of tumors, an optimal cluster number needs to be determined. TIPC employs an empiric approach that jointly tests cluster numbers and subregion size to minimize selection bias and provide information about the robustness of any given TIPC clustering solution. Based on the consensus CDF delta curve generated by *consensus_clustering*, the minimal number of clusters corresponding to a robust clustering solution occurs at the *k* value beyond which there is no additional significant gain in clustering consensus (i.e., the relative change in the area under the CDF curve is close to 0). A companion tracking plot also generated by *consensus_clustering* allows for further examination and identification of larger cluster numbers that yield stable cluster assignments. While the selection of cluster number also depends on data characteristics, such as heterogeneity, the *optimal_k* function in the TIPC package supports broader adoption by minimizing user intervention. This function identifies the optimal cluster number by first determining the smallest *k* value to ensure stability (i.e., the shoulder point of the CDF delta curve) and then selecting the largest *k* in the tracking plot (i.e., the point where normalized mutual information consistently remains high) to ensure granularity.

Small clusters (hereafter called outlier clusters) can be produced because of a suboptimal choice of subregion size and number of clusters, or in the presence of rare distribution patterns generated from technical artefact or true yet rare biologic processes. An outlier cluster may be underpowered for downstream analysis. Survival analysis based on *postTIPC_SurvivalAnalysis* excludes clusters containing less than 30 tumors in this study, although it is likely that this number needs to be modified for other studies with different cohort sizes. Overall, an optimal choice of subregion size and number of clusters depends on the cancer types and immune cell of interest under study.

### Extrapolation of TIPC identified spatial patterns in The Cancer Genome Atlas

Using the subregion size of 35 μm as determined in the TIPC analysis of NHS/HPFS CRC cohorts [24,25], we first computed the six-element TIPC spatial parameters using eosinophils identified morphologically in H&E images of 571 tumors obtained from TCGA CRC cohort. We then assigned each of these tumors to the most similar TIPC spatial pattern as determined in NHS/HPFS cohorts, based on kNN algorithm by considering 10 nearest neighbors (*class* R package). Of note, due to the variable quality of H&E images in the TCGA dataset—likely stemming from differences in tissue processing, staining, and legacy scanning methods—we focused only on eosinophils in this study. Eosinophils can be identified with high confidence based on their distinctive eosinophilic granules, which are less impacted by image quality variability compared to other cell types, such as neutrophils and plasma cells.

### Statistical analysis

All analyses were conducted using the R software environment (version 3.6.1). Statistical significance was judged using the two-sided α level of 0.005 and p values between 0.005 and 0.05 were interpreted as suggestive evidence. Box plots of immune cell densities were defined by the 25th percentile (lower box boundary) and 75th percentile (upper box boundary) with lower and upper whiskers marking the minimum (lower) and maximum densities (upper); jittered dots represent individual case values. In forest plots of CRC-specific survival for tumor subtypes, whiskers depict the magnitude of the confidence interval: lower 95% (left) and upper 95% (right).

Prognostic analysis was performed based on 10-year CRC-specific survival outcome using either Kaplan-Meier estimates with a log-rank test (*survminer* R package), or a univariable or multivariable Cox proportional hazards regression model (*survival* R package). The NND or immune density quartiles and tumor spatial subtypes were analyzed as categorical covariates in these models, where the 1^st^ quartile of NND or immune density and the spatial subtype with the lowest average immune cell density were used as references, respectively, with an exception that “cold, stroma-rich” subtype was used as the reference in eosinophil and neutrophil analyses as this subtype showed significantly worse CRC-specific prognosis than “cold, tumor-rich” subtype despite of its higher cell density. Note that in these analyses of CRC-specific mortality, deaths resulting from other causes were censored.

In the multivariable Cox regression analyses, covariates assessed as potential confounding factors included sex (female vs. male), age at diagnosis (continuous), year of diagnosis (continuous), family history of CRC in any first-degree relative (present vs. absent), tumor location (proximal colon vs. distal colon vs. rectum), tumor differentiation (well to moderate vs. poor), disease stage (I/II vs. III/IV), MSI status (MSI-high vs. non-MSI-high), CpG island methylator phenotype (CIMP) status (high vs. low/negative), long-interspersed nucleotide element-1 (LINE-1) methylation level (continuous), *KRAS* mutation (mutant vs. wild-type), *BRAF* mutation (mutant vs. wild-type), and *PIK3*CA mutation (mutant vs. wild-type). Cases with missing data were included in the majority category of a given categorical covariate to limit the degrees of freedom: family history of CRC in a first-degree relative (0.4%), tumor location (0.4%), tumor differentiation (0.1%), disease stage (7.2%), MSI (2.9%), CIMP (7.1%), *KRAS* (2.9%), *BRAF* (2.0%), and *PIK3*CA (8.7%). For the cases with missing LINE-1 methylation data (2.8%), we substituted the mean value and assigned a separate indicator variable for missing cases. To confirm appropriate covariate selection, we also performed feature selection for confounding factors by fitting a generalized linear model (using Cox regression model as the link function) with Lasso regularization (*glmnet* R package), whereby the optimal regularization parameter lambda was selected from a 5-fold cross validation test. Our data showed that similar p-values and hazard ratio central estimates were obtained as that of a multivariable Cox regression model without selection.

We employed the Cochran-Armitage trend test to examine associations between TIPC spatial subtypes (categorical variable) and clinicopathologic and molecular features (ordinal variables), including 5-year CRC-specific survival, microsatellite instability (MSI) status, CpG island methylator phenotype (CIMP) status, lymphocytic reaction patterns [13], neoantigen load, [40] and immunohistochemistry-based protein expression of CD274 (PD-L1), CDH1, CTNNB1, PDCD1LG2 (PD-L2), PTGER2, SQSTM1, and YAP1. Considering the limited size of HCC cohort, we employed the non-parametric Kruskal Wallis test to examine the relationship between TIPC spatial subtypes (a categorical variable) and patient response outcomes. The patient responses were categorized as ordinal variables, arranged sequentially from non-responders, to progressors, and with super responders representing the highest category of response.

## Supporting information

S1 Fig Evaluation of effect of subregion size on TIPC spatial parameter value distribution, using CD3+ T-cells. Two representative regions of interest demonstrating different subregion sizes in (**a**) a stromal region and (**b**) a tumor region predominating colorectal cancer tissue sections. (**c**) Distribution of TIPC spatial parameter values (in normalized counts) across a range of subregion sizes, i.e., 20–55 μm. Subregion sizes smaller than 30 μm demonstrated an underrepresented I:T low measure. Abbreviations: I:T, immune-to-tumor, I:S, immune-to-stroma.
(PDF)

S2 Fig Determination of optimal subregion size and input cluster number (*k*) for TIPC analysis using CD3+ T cells. At individual subregion sizes of (**a**-**c**) 30, (**d**-**f**) 35, and (**g**-**i**) 40 μm, (**a**,**d**,**g**) cumulative distribution function (CDF) delta plots were first used to determine the minimum *k* for stable clustering (colored in red); (**b**,**e**,**h**) tracking plots revealed the relationship between granularity (high *k* yields high granularity) and cluster size (optimal *k*, marked by black boxes, were selected manually for ensuring a balance between granularity and statistical power). After excluding clusters comprising less than 30 tumors, (**c**,**f**,**i**) the major clusters with their spatial patterns represented by the six TIPC parameters were shown in the heat maps. Subregion sizes 30 and 35 μm yielded six largely similar patterns whereas the HC cluster was missing from subregion size of 40 μm. Abbreviations, CSR = cold, stroma-rich, CTR = cold, tumor-rich, HD = hot and disperse, HTCC = hot, tumor-centric clustering, HSCC = hot, stroma-centric clustering, HC = hot and clustered.
(PDF)

S3 Fig Cox proportional hazards regression analysis based on TIPC spatial subtypes identified using CD3+ T-cells at subregion = 35 μm and input *k* = 9. Forest plots depicting hazard ratios and 95% confidence intervals of univariable and multivariable models which were adjusted for (**a**) clinicopathologic features, or (**b**) both clinicopathologic features and cell density. Abbreviations, CSR = cold, stroma-rich, CTR = cold, tumor-rich, HD = hot and disperse, HTCC = hot, tumor-centric clustering, HSCC = hot, stroma-centric clustering, HC = hot and clustered. Symbols *** p < 0.001, ** p < 0.01, * p < 0.05, not significant (ns) p > 0.05.
(PDF)

S4 Fig Survival analysis based on cell density and nearest neighbor distance (NND), using CD3+, CD3+CD8+CD45RO+, eosinophils, and neutrophils, in Nurses’ Health Study/Health Professionals Follow-up Study cohorts. Tumors were either grouped into quartiles based on (**a**) their overall cell densities (using a univariable Cox regression model) or (**b**) nearest neighbor distance (NND) to the nearest tumor cells (using both univariable and multivariable Cox regression models by adjusting for either only cell density or cell density with clinicopathological features). Symbols *** p < 0.001, ** p < 0.01, * p < 0.05, not significant (ns) p > 0.05.
(PDF)

S5 Fig Morisita-Horn (M-H) analysis using CD3+ T cells. M-H index was first computed using 4.5-by-4.5, 5-by-5, 5.5-by-5.5, and 6-by-6 μm rectangular grids, measuring the degree of co-localization between CD3+ T cells with (left panel) tumor or (right panel) stromal cells. The tumors were then assigned to M-H low or high groups using the percentile cut-offs (represented by horizontal axis). Univariate Cox PH regression models were used to test for prognostic significance associated with tumors showing high versus low co-localization. Vertical axis indicates logarithmic transformed false discovery rate (FDR) values adjusted for the 13 cut-offs. The red dotted lines mark FDR = 0.05. Two combinations harbored significant associations (FDR ≤ 0.05) with colorectal cancer-specific survival for both discovery and validation subsets and are highlighted in red boxes with hazard ratios (HRs) and confidence intervals (CIs) labeled on top. ns for not significant, i.e., p > 0.05.
(PDF)

S6 Fig Prognostic performance evaluation of M-H subtypes derived from CD3+ T cells. At the two optimal grid sizes (5-by-5 and 6-by-6 μm, see S5 Fig for full details), both using a cut-off = 0.8 for dichotomizing tumors into low and high subtypes, both M-H solutions showed (**a**) confounding effects of overall CD3+ T-cell densities and (**b**) significant prognostic associations based on Kaplan-Meier estimates and log-rank test where P-values < 0.05.
(PDF)

S7 Fig Performance evaluation of G-cross subtypes identified using CD3+ T cells. G-cross area under the curve (AUC), based (left panel) overall tissue regions and (right panel) stromal regions, was measured at *r* ≤ 20 μm and tumors were grouped into quartile categories of AUC. (**a**) Both analyses showed a significant confounding effect for overall CD3+ T cell density. (**b**) Only subtypes identified using CD3+ T cells in the overall tissue region showed prognostic significance value based on Kaplan-Meier estimates and the log-rank test.
(PDF)

S8 Fig Performance evaluation of L-cross subtypes identified using CD3+ T cell. L-cross area under the curve (AUC), based (left panel) overall tissue regions and (right panel) stromal regions, was measured at *r* ≤ 20 μm and tumors were grouped into quartile categories. (**a**) Both of these subtypes showed modest confounding due to overall CD3+ T cell densities and (**b**) no significant prognostic utility as assessed by Kaplan-Meier estimates and the log-rank test.
(PDF)

S9 Fig Heat-map summarizes the association between molecular and pathologic features and TIPC spatial subtypes derived using CD3+ T cells. Extended Cochran–Armitage method was used to test the association significance between molecular and pathologic features (ordered variables) and TIPC spatial subtypes (unordered variable). Horizontal axis indicates cluster names: cold, stroma-rich (CSR), cold, tumor-rich (CTR), hot, tumor-centric clustering (HTCC), hot and disperse (HD), hot, stroma-centric clustering (HTCC), and hot and clustered (HC).
(PDF)

S10 Fig Survival analysis of TIPC subtypes derived from three different immune cell types, cytotoxic memory T cells (CD3+CD8+CD45RO+), eosinophils, and neutrophils in Nurses’ Health Study/Health Professionals Follow-up Study CRC datasets [24,25]. Kaplan-Meier and log-rank test show that the TIPC spatial subtypes of, (**a**) cytotoxic memory T cells (CD3+CD8+CD45RO+), (**b**) eosinophils, and (**c**) neutrophils, were significantly associated with colorectal cancer-specific survival. Abbreviations, CSR = cold, stroma-rich, CTR = cold, tumor-rich, HD = hot and disperse, HTCC = hot, tumor-centric clustering, HSCC = hot, stroma-centric clustering, HC = hot and clustered, HCTR = hot and clustered, tumor-rich, and HCSR = hot and clustered, stroma-rich.
(PDF)

S11 Fig Cox proportional hazards regression analysis based on TIPC spatial subtypes derived from cytotoxic memory T cells (CD3+CD8+CD45RO+), eosinophils, and neutrophils in NHS/ HPFS CRC datasets[24,25]; multivariable Cox Nurses’ Health Study/Health Professionals Follow-up Study adjusted for clinicopathologic features. Forest plots associated with the subtypes of, (**a**) cytotoxic memory T cells (CD3+CD8+CD45RO+), (**b**) eosinophils, and (**c**) neutrophils, depicting hazard ratios and 95% confidence intervals for both univariable and multivariable (adjusted for clinicopathologic features). Abbreviations, CSR = cold, stroma-rich, CTR = cold, tumor-rich, HD = hot and disperse, HTCC = hot, tumor-centric clustering, HSCC = hot, stroma-centric clustering, HC = hot and clustered, HCTR = hot and clustered, tumor-rich, and HCSR = hot and clustered, stroma-rich. Symbols *** p < 0.001, ** p < 0.01, * p < 0.05, not significant (ns) p > 0.05.
(PDF)

S12 Fig Cox proportional hazards regression analysis based on TIPC spatial subtypes derived from cytotoxic memory T cells (CD3+CD8+CD45RO+), eosinophils, and neutrophils in NHS/ HPFS; multivariable Cox proportional hazards adjusted for clinicopathologic features and cell densities. Forest plots associated with the subtypes of, (**a**) cytotoxic memory T cells (CD3+CD8+CD45RO+), (**b**) eosinophils, and (**c**) neutrophils, depicting hazard ratios and 95% confidence intervals for both univariable and multivariable (adjusted for both clinicopathologic features and cell density). Abbreviations, CSR = cold, stroma-rich, CTR = cold, tumor-rich, HD = hot and disperse, HTCC = hot, tumor-centric clustering, HSCC = hot, stroma-centric clustering, HC = hot and clustered, HCTR = hot and clustered, tumor-rich, and HCSR = hot and clustered, stroma-rich. Symbols *** p < 0.001, ** p < 0.01, * p < 0.05, not significant (ns) p > 0.05.
(PDF)

S13 Fig Evaluation of effect of subregion size on TIPC spatial parameter value distribution, using eosinophils. Distribution of TIPC spatial parameter values (in normalized counts) across a range of subregion sizes, i.e., 20-55 μm. I:T low and I:S low were generally under-represented, and a subregion size of 35 μm reached a plateau for Tumor-only an underrepresented I:T low measure. Abbreviations: I:T, immune-to-tumor, I:S, immune-to-stroma.
(PDF)

S14 Fig Determination of optimal subregion size and input cluster number (*k)* for TIPC analysis using eosinophils. At individual subregion sizes of (**a**-**c**) 30, (**d**-**f**) 35, and (**g**-**i**) 40 μm, (**a**,**d**,**g**) cumulative distribution function (CDF) delta plots were first used to determine the minimum *k* for stable clustering (colored in red); (**b**,**e**,**h**) tracking plots revealed the relationship between granularity (high *k* yields high granularity) and cluster size (optimal *k*, marked by black boxes, were selected manually for ensuring a balance between granularity and statistical power). After excluding clusters comprising less than 30 tumors, (**c**,**f**,**i**) the major clusters with their spatial patterns represented by the six TIPC parameters were shown in the heat maps. The three TIPC solutions yielded similar spatial subtypes except that instead of HSCC subtype detected at sizes 25 and 30 μm, HC subtype was found at 35 μm, as (**c**,**f**) the former showed a relatively less coherent spatial profile, TIPC solution determined at 35 μm was used for downstream association analysis. Abbreviations: CSR = Cold, stroma-rich; CTR = Cold, tumor-rich; HD = Host and disperse; HSCC = Hot, stroma-centric clustering; HCTR = Host and clustered, tumor-rich.
(PDF)

S15 Fig Evaluation of effect of subregion size on TIPC spatial parameter value distribution, using neutrophils. Distribution of TIPC spatial parameter values (in normalized counts) across a range of subregion sizes, i.e., 20-55 μm. Subregion size ≥ 40 μm ensures minimal detection of I:S low and I:T low. Abbreviations: I:T, immune-to-tumor, I:S, immune-to-stroma.
(PDF)

S16 Fig Determination of optimal subregion size and input cluster number (*k)* for TIPC analysis using neutrophils. At individual subregion sizes of (**a**-**c**) 35, (**d**-**f**) 40, and (**g**-**i**) 50 μm, (**a**,**d**,**g**) cumulative distribution function (CDF) delta plots were first used to determine the minimum *k* for stable clustering (colored in red); (**b**,**e**,**h**) tracking plots revealed the relationship between granularity (high *k* yields high granularity) and cluster size (optimal *k*, marked by black boxes, were selected manually for ensuring a balance between granularity and statistical power). After excluding clusters comprising less than 30 tumors, (**c**,**f**,**i**) the major clusters with their spatial patterns represented by the six TIPC parameters were shown in the heat maps. Similar spatial subtypes were obtained using subregion size across 35-50 μm, except that instead of HCSR subtype detected at both sizes 35 and 50 μm (more robust), HSCC subtype was found at 40 μm, hence, TIPC solution determined at 35 μm (alternatively, 50 μ m could also be used) was used for downstream association analysis. Abbreviations: CSR = Cold, stroma-rich; CTR = Cold, tumor-rich; HD = Host and disperse; HSCC = Hot, stroma-centric clustering; HCTR = Host and clustered, tumor-rich.
(PDF)

S17 Fig Performance evaluation on the effect of subregion sizes and input cluster number (*k*) on spatial subtype identification and prognostic significance, using CD3+ T cells. TIPC analysis was performed using subregion sizes in the range of 30-50 μm, at each of these subregion sizes, input cluster numbers in the range of 4-10 were tested whereby univariate Cox regression model was used to test the association significance of the resulting TIPC subtypes with colorectal cancer-specific survival; subtypes comprising <30 tumors were excluded. Vertical axis indicates CD3+ T cell density (cells/mm2) for TIPC subtype. Subtypes were ordered based on their mean CD3+ T cell density, from the lowest (reference cluster) on the left to highest on the right; symbol size reflects the relative cluster size. Abbreviations: CSR = Cold, stroma-rich; CTR = Cold, tumor-rich; HTCC = Hot, tumor-centric clustering; HD = Host and disperse; HSCC = Hot, stroma-centric clustering; HC = Hot and clustered; HR = hazard ratio.
(PDF)

S18 Fig Performance evaluation on the effect of subregion sizes and input cluster number (*k*) on spatial subtype identification and prognostic significance, using neutrophils. TIPC analysis was performed using subregion sizes in the range of 35-55 μm, at each of these subregion sizes, input cluster numbers in the range of 4-10 were tested whereby univariate Cox regression model was used to test the association significance of the resulting TIPC subtypes with CRC-specific survival (reference cluster: CSR); subtypes comprising <30 tumors were excluded. Vertical axis indicates neutrophils density (cells/mm2) for TIPC subtype. Subtypes were ordered based on their mean neutrophil density, from the lowest on the left to highest on the right; symbol size reflects the relative cluster size. Abbreviations: CTR = Cold, tumor-rich; HTCC = Hot, tumor-centric clustering; HD = Host and disperse; HSCC = Hot, stroma-centric clustering; HCTR = Host and clustered, tumor-rich; HR = hazard ratio.
(PDF)

S19 Fig Determination of optimal subregion size and cluster number (k) and the clustering results using TIPC package functions *optimal_hexLen* and *optimal_k*. Using (a-c) cytotoxic memory T cells, (d-f) eosinophils, and (g-i) neutrophils in CRC (NHS/HPFS), *optimal_hexLen* identified 70, 80, and 80 as the optimal subregion sizes, respectively. Then, *optimal_k* identified the shoulder points as the smallest cluster numbers—(a) 4, (d) 4, and (g) 4, respectively—to ensure stability, followed by selecting the largest stable k values—(b) 9, (e) 10, and (h) 10, respectively—to ensure granularity. After removing outlier clusters containing fewer than 30 samples, the resulting clusters were (c) 7, (f) 5, and (i) 5 spatial clusters, closely matching those generated by manual selection. Abbreviations: CSR = Cold, stroma-rich; CTR = Cold, tumor-rich; HD = Host and disperse; HC = hot and clustered; HTCC = hot, tumor-centric; HSCC = Hot, stroma-centric clustering; HCTR = Host and clustered, tumor-rich; HCSR = hot and clustered, stroma-rich.
(PDF)

S20 Fig Validation of the prognostic significance of TIPC subtypes which were first determined in Nurses’ Health Study/Health Professionals Follow-up Study (NHS/HPFS) CRC cohort[24,25] and later recapitulated in TCGA cohort, using eosinophils identified morphologically in H&E images. Among the five major TIPC subtypes determined in NHS/ HPFS cohort, three comprised of >30 tumors (i.e., CTR, HD, HCTR; see Fig 8). (a-b) Kaplan-Meier estimates associated with these TIPC subtypes of eosinophil subtypes harbored significant association with (a) disease-specific survival (DSS) and (b) progression-free intervals (PFI); (**c**) forest plot summarizes Cox regression analysis of tumor subtypes determined using cell density or nearest neighbor distance (NND) (see Fig 8 for the associations with overall survival). Symbols *** p < 0.001, ** p < 0.01, * p < 0.05, not significant (ns) p > 0.05. Abbreviations: CTR = Cold, tumor-rich; HD = Host and disperse; HCTR = Host and clustered, tumor-rich.
(PDF)

S21 Fig Morisita-Horn (M-H) analysis using eosinophils identified in Nurses’ Health Study/Health Professionals Follow-up Study CRC cohorts [24,25]. M-H index was first computed using rectangular grid sizes of 4.5-by-4.5, 5-by-5, 5.5-by-5.5, and 6-by-6 μm, measuring the co-localization between eosinophils with stromal (left panel) or tumor cells (right panel). The tumors were then assigned to M-H low or high groups using the percentile cut-offs (represented by horizontal axis). Univariate Cox proportional hazards regression models were used to test for prognostic significance associated with tumors showing high (M-H high group) versus low (M-H low group) co-localization. Vertical axis indicates logarithmic transformed false discovery rate (FDR) values adjusted for the 13 cut-offs. The red dotted lines mark FDR = 0.05. Combinations harbored significant associations (FDR ≤ 0.05) with colorectal cancer-specific survival for both discovery and validation subsets and were highlighted in red boxes.
(PDF)

S22 Fig Comparison of the number of tumors in Morisita-Horn (M-H) low and high groups in Nurses’ Health Study/Health Professionals Follow-up Study (NHS/ HPFS) [24,25] and The Cancer Genome Atlas (TCGA) cohorts. M-H index was computed using rectangular grid sizes of 4.5-by-4.5, 5-by-5, 5.5-by-5.5, and 6-by-6 μm, measuring the co-localization between eosinophils with stromal (left panel) or tumor cells (right panel), in (**a**) NHS/HPFS and (**b**) TCGA cohorts, individually. Based on the percentiles (represented by horizontal axis) determined in (**a**) NHS/ HPFS cohorts, (**b**) TCGA cohort were divided into M-H low and high groups, where TCGA cohort showed an extremely skewed distribution with very few tumors assigned to the M-H low group across all the combinations of grid sizes and cut-offs.
(PDF)

S1 Table Definition of ‘spatial’ terminology.
(PDF)

S2 Table Definition of the six TIPC spatial parameters.
(PDF)

S3 Table Evaluation of the confounding effect of CD3+ T-cell density on tumor subtypes derived by existing spatial analysis methods. Multivariable Cox proportional hazards model included both the tumor subtypes identified by (a-b) the Morisita-Horn (M-H) index, (c) G-cross, and (d) L-cross functions and overall CD3+ T cell density (quartiles). M-H subtypes were generated using (a) 5-by-5 and (b) 6-by-6 μm grid sizes based on the validation dataset (see S5 Fig for details). HR, hazard ratio; CI, confidence interval.
(PDF)

S4 Table Comparison of prognostic significance between tumor subtypes derived by TIPC and other existing methods. Multivariable Cox proportional hazards model included both the tumor subtypes identified by TIPC and (a) CD3+ T cell density quartiles, (b) Morisita-Horntumor:CD3+T cell index quartiles (using a 5-by-5 μm grid and 80th percentile dichotomization cut-off), (c) G-crosstumor:CD3+T cell (in stroma) AUC quartiles (r < 20 μm), and (d) L-crosstumor:CD3+T cell (in stroma) AUC quartiles (r < 20 μm). Abbreviations: CSR = Cold, stroma-rich; CTR = Cold, tumor-rich; HTCC = Hot, tumor-centric clustering; HD = Host and disperse; HSCC = Hot, stroma-centric clustering; HC = Hot and clustered; HCTR = Host and clustered, tumor-rich; HCSR = Hot and clustered, stroma-rich.; HR = hazard ratio; CI = confidence interval.
(PDF)

S5 Table Evaluation of the confounding effect of immune cell density on TIPC subtypes. Multivariable Cox proportional hazards model included TIPC subtypes of (a) cytotoxic memory T cells, (b) eosinophils, and (c) neutrophils, and the corresponding overall cell density (quartiles). Abbreviations: CSR = Cold, stroma-rich; CTR = Cold, tumor-rich; HTCC = Hot, tumor-centric clustering; HD = Host and disperse; HSCC = Hot, stroma-centric clustering; HC = Hot and clustered; HCTR = Host and clustered, tumor-rich; HCSR = Hot and clustered, stroma-rich; HR = hazard ratio; CI = confidence interval.
(PDF)

S6 Table Qualitative descriptions for distinct tumor-immune spatial patterns identified using TIPC.
(PDF)
