# Supplementary material for: Tumor-immune partitioning and clustering algorithm for identifying tumor-immune cell spatial interaction signatures within the tumor microenvironment
Source: PLoS Comput Biol. 2025 Feb 18;21(2):e1012707. doi: 10.1371/journal.pcbi.1012707 (PMC11849983; doi:10.1371/journal.pcbi.1012707)
Supplement: S1 Fig — Evaluation of effect of subregion size on TIPC spatial parameter value distribution, using CD3+ T-cells. Two representative regions of interest demonstrating different subregion sizes in (a) a stromal region and (b) a tumor region predominating colorectal cancer tissue sections. (c) Distribution of TIPC spatial parameter values (in normalized counts) across a range of subregion sizes, i.e., 20–55 μm. Subregion sizes smaller than 30 μm demonstrated an underrepresented I:T low measure. Abbreviations: I:T, immune-to-tumor, I:S, immune-to-stroma. (PDF) [file pcbi.1012707.s001.pdf]

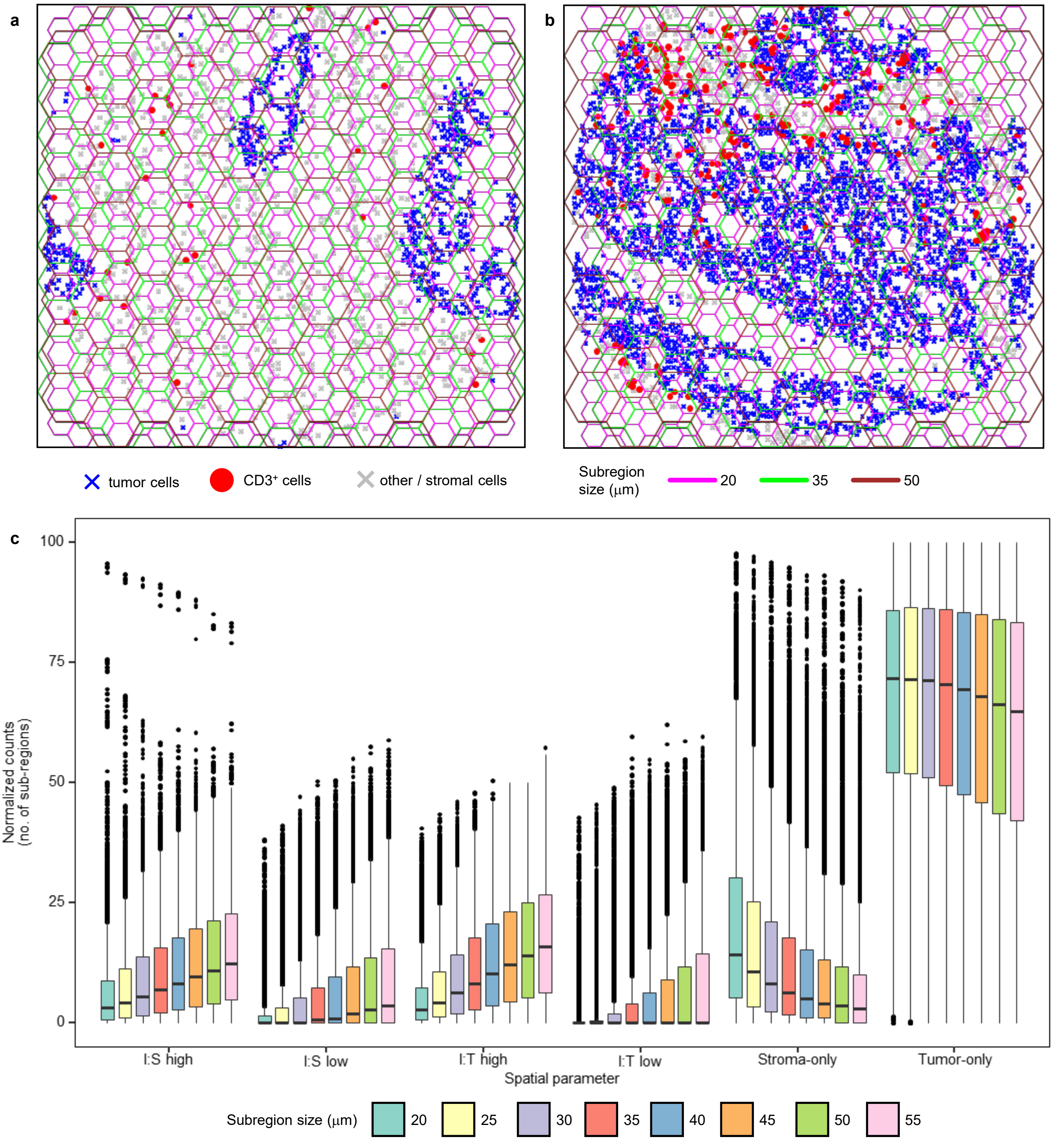

S1 Figure. Evaluation of effect of subregion size on TIPC spatial parameter value distribution, using $CD3^+$ T-cells. Two representative regions of interest demonstrating different subregion sizes in (**a**) a stromal region and (**b**) a tumor region predominating colorectal cancer tissue sections. (**c**) Distribution of TIPC spatial parameter values (in normalized counts) across a range of subregion sizes i.e., 20-55 μm. Subregion sizes smaller than 30 μm demonstrated an underrepresented I:T low measure. Abbreviations: I:T, immune-to-tumor, I:S, immune-to-stroma.
