# Supplementary material for: Tumor-immune partitioning and clustering algorithm for identifying tumor-immune cell spatial interaction signatures within the tumor microenvironment
Source: PLoS Comput Biol. 2025 Feb 18;21(2):e1012707. doi: 10.1371/journal.pcbi.1012707 (PMC11849983; doi:10.1371/journal.pcbi.1012707)
Supplement: S2 Fig — Determination of optimal subregion size and input cluster number (k) for TIPC analysis using CD3+ T cells. At individual subregion sizes of (a-c) 30, (d-f) 35, and (g-i) 40 μm, (a,d,g) cumulative distribution function (CDF) delta plots were first used to determine the minimum k for stable clustering (colored in red); (b,e,h) tracking plots revealed the relationship between granularity (high k yields high granularity) and cluster size (optimal k, marked by black boxes, were selected manually for ensuring a balance between granularity and statistical power). After excluding clusters comprising less than 30 tumors, (c,f,i) the major clusters with their spatial patterns represented by the six TIPC parameters were shown in the heat maps. Subregion sizes 30 and 35 μm yielded six largely similar patterns whereas the HC cluster was missing from subregion size of 40 μm. Abbreviations, CSR = cold, stroma-rich, CTR = cold, tumor-rich, HD = hot and disperse, HTCC = hot, tumor-centric clustering, HSCC = hot, stroma-centric clustering, HC = hot and clustered. (PDF) [file pcbi.1012707.s002.pdf]

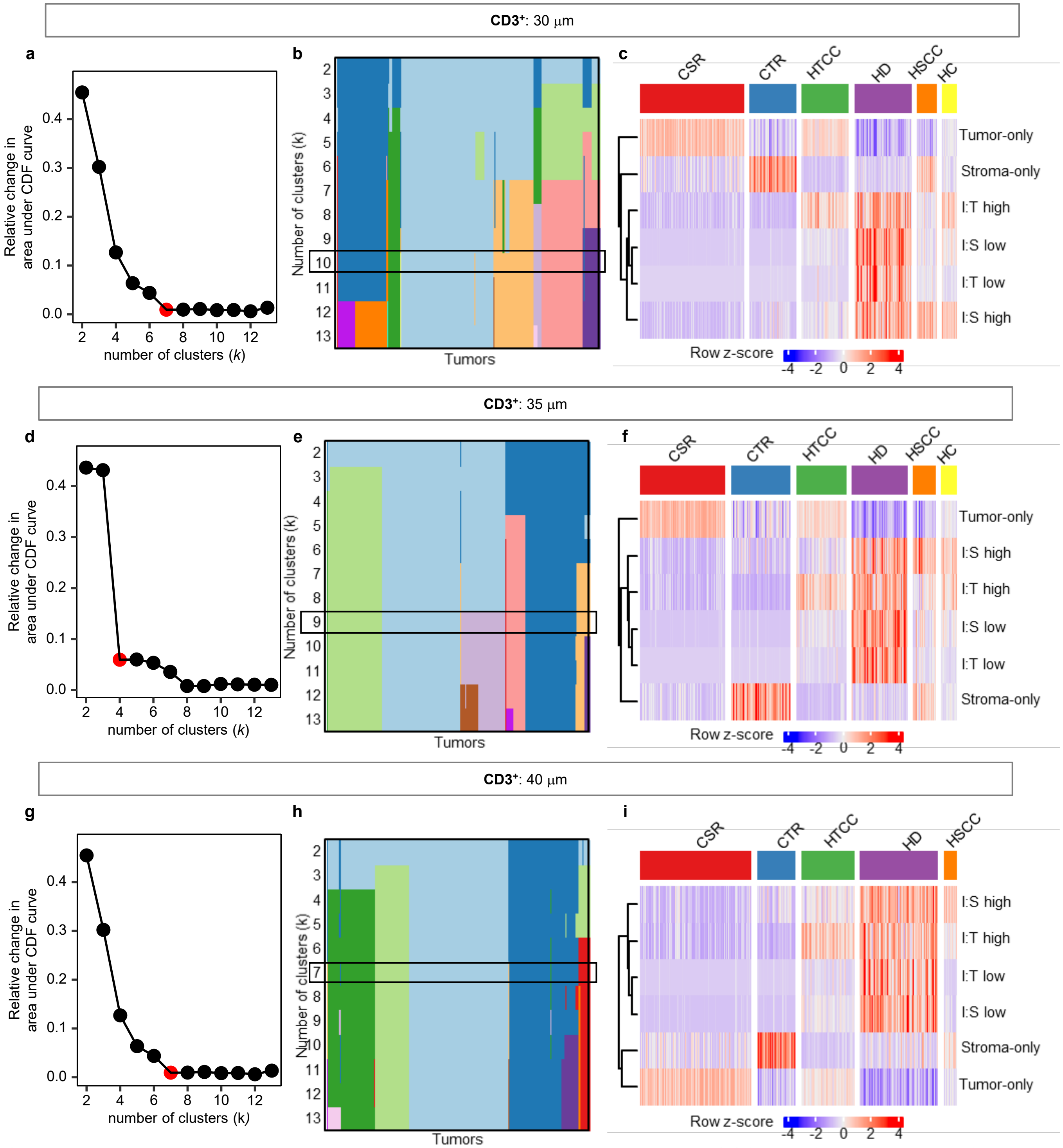

S2 Figure. Determination of optimal subregion size and input cluster number (*k*) for TIPC analysis using $CD3^+$ T cells. At individual subregion sizes of (**a**-**c**) 30, (**d**-**f**) 35, and (**g**-**i**) 40 μm, (**a**,**d**,**g**) cumulative distribution function (CDF) delta plots were first used to determine the minimum *k* for stable clustering (colored in red); (**b**,**e**,**h**) tracking plots revealed the relationship between granularity (high *k* yields high granularity) and cluster size (optimal *k*, marked by black boxes, were selected manually for ensuring a balance between granularity and statistical power). After excluding clusters comprising less than 30 tumors, (**c**,**f**,**i**) the major clusters with their spatial patterns represented by the six TIPC parameters were shown in the heat maps. Subregion sizes 30 and 35 μm yielded six largely similar patterns whereas the HC cluster was missing from subregion size of 40 μm. Abbreviations, CSR = cold, stroma-rich, CTR = cold, tumor-rich, HD = hot and disperse, HTCC = hot, tumor-centric clustering, HSCC = hot, stroma-centric clustering, HC = hot and clustered.
