# Supplementary material for: Tumor-immune partitioning and clustering algorithm for identifying tumor-immune cell spatial interaction signatures within the tumor microenvironment
Source: PLoS Comput Biol. 2025 Feb 18;21(2):e1012707. doi: 10.1371/journal.pcbi.1012707 (PMC11849983; doi:10.1371/journal.pcbi.1012707)
Supplement: S3 Fig — Cox proportional hazards regression analysis based on TIPC spatial subtypes identified using CD3+ T-cells at subregion = 35 μm and input k = 9. Forest plots depicting hazard ratios and 95% confidence intervals of univariable and multivariable models which were adjusted for (a) clinicopathologic features, or (b) both clinicopathologic features and cell density. Abbreviations, CSR = cold, stroma-rich, CTR = cold, tumor-rich, HD = hot and disperse, HTCC = hot, tumor-centric clustering, HSCC = hot, stroma-centric clustering, HC = hot and clustered. Symbols *** p < 0.001, ** p < 0.01, * p < 0.05, not significant (ns) p > 0.05. (PDF) [file pcbi.1012707.s003.pdf]

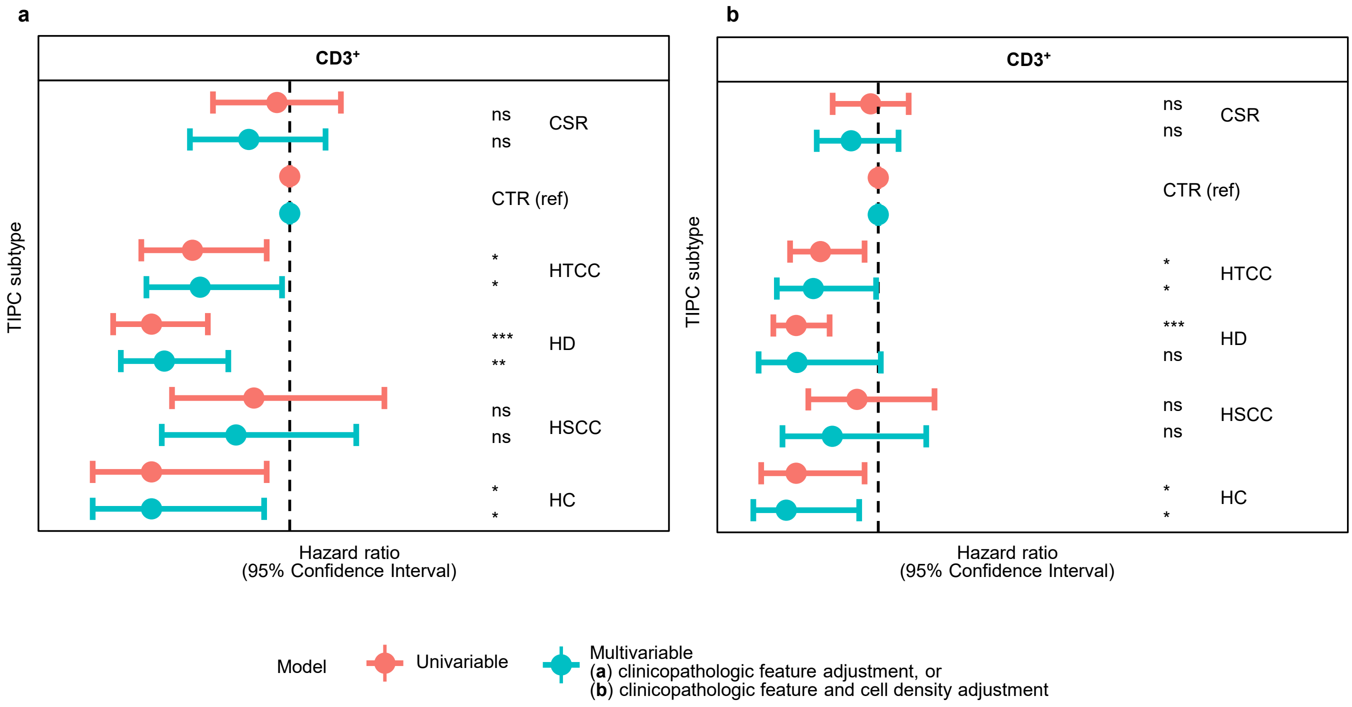

S3 Figure. Cox proportional hazards regression analysis based on TIPC spatial subtypes identified using $CD3^+$ T-cells at subregion = 35 μm and input *k* = 9. Forest plots depicting hazard ratios and 95% confidence intervals of univariable and multivariable models which were adjusted for (**a**) clinicopathologic features, or (**b**) both clinicopathologic features and cell density. Abbreviations, CSR = cold, stroma-rich, CTR = cold, tumor-rich, HD = hot and disperse, HTCC = hot, tumor-centric clustering, HSCC = hot, stroma-centric clustering, HC = hot and clustered. Symbols *** $p < 0.001$, ** $p < 0.01$, * $p < 0.05$, not significant (ns) $p > 0.05$.
