# Supplementary material for: Tumor-immune partitioning and clustering algorithm for identifying tumor-immune cell spatial interaction signatures within the tumor microenvironment
Source: PLoS Comput Biol. 2025 Feb 18;21(2):e1012707. doi: 10.1371/journal.pcbi.1012707 (PMC11849983; doi:10.1371/journal.pcbi.1012707)
Supplement: S4 Fig — Survival analysis based on cell density and nearest neighbor distance (NND), using CD3+, CD3+CD8+CD45RO+, eosinophils, and neutrophils, in Nurses’ Health Study/Health Professionals Follow-up Study cohorts. Tumors were either grouped into quartiles based on (a) their overall cell densities (using a univariable Cox regression model) or (b) nearest neighbor distance (NND) to the nearest tumor cells (using both univariable and multivariable Cox regression models by adjusting for either only cell density or cell density with clinicopathological features). Symbols *** p < 0.001, ** p < 0.01, * p < 0.05, not significant (ns) p > 0.05. (PDF) [file pcbi.1012707.s004.pdf]

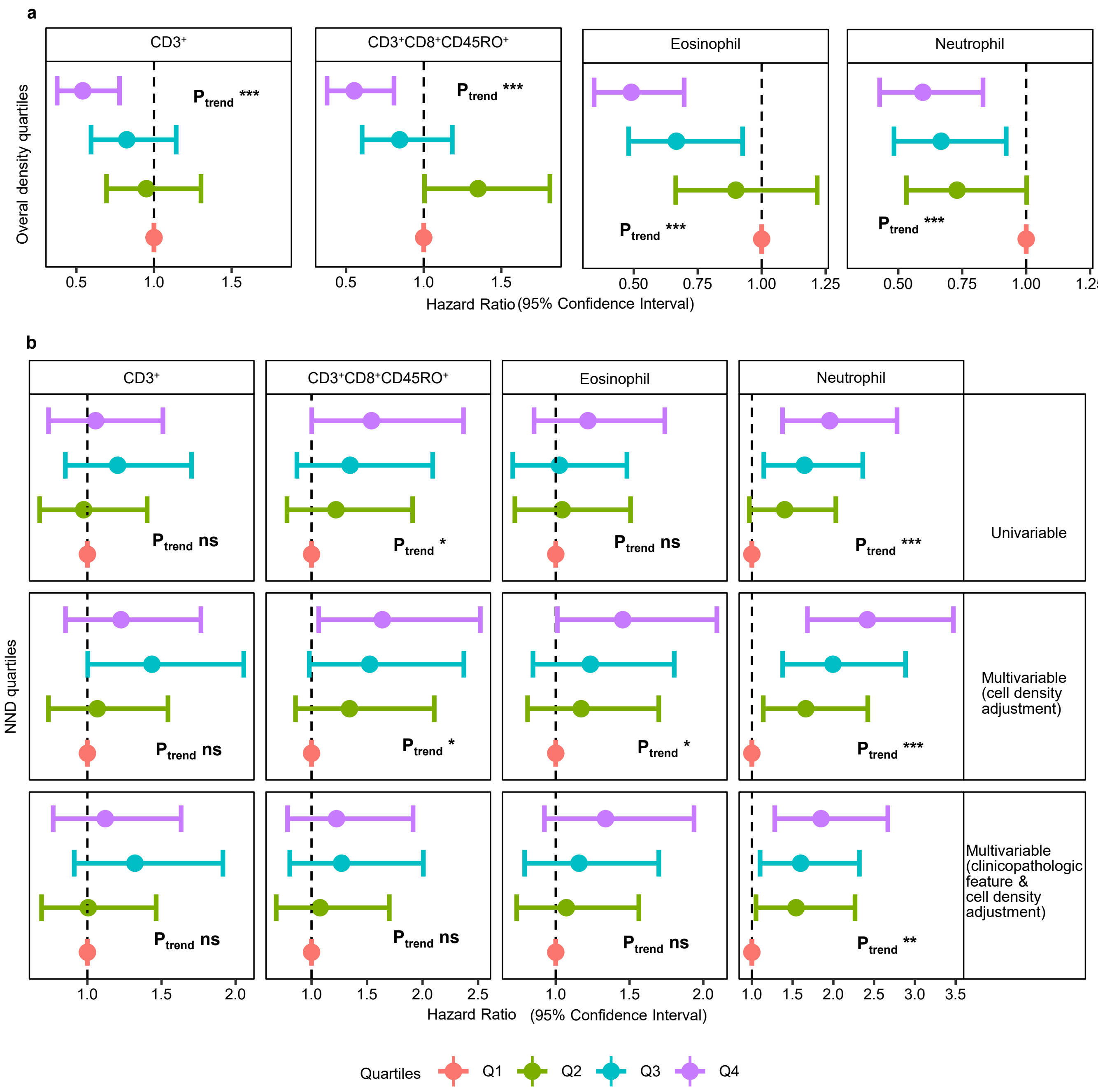

S4 Figure. Survival analysis based on cell density and nearest neighbor distance (NND), using $CD3^+$, $CD3^+CD8^+CD45RO^+$, eosinophils, and neutrophils, in Nurses' Health Study/Health Professionals Follow-up Study cohorts. Tumors were either grouped into quartiles based on (**a**) their overall cell densities (using a univariable Cox regression model) or (**b**) nearest neighbor distance (NND) to the nearest tumor cells (using both univariable and multivariable Cox regression models by adjusting for either only cell density or cell density with clinicopathological features). Symbols *** $p < 0.001$, ** $p < 0.01$, * $p < 0.05$, not significant (ns) $p > 0.05$.
