# Supplementary material for: Tumor-immune partitioning and clustering algorithm for identifying tumor-immune cell spatial interaction signatures within the tumor microenvironment
Source: PLoS Comput Biol. 2025 Feb 18;21(2):e1012707. doi: 10.1371/journal.pcbi.1012707 (PMC11849983; doi:10.1371/journal.pcbi.1012707)
Supplement: S5 Fig — Morisita-Horn (M-H) analysis using CD3+ T cells. M-H index was first computed using 4.5-by-4.5, 5-by-5, 5.5-by-5.5, and 6-by-6 μm rectangular grids, measuring the degree of co-localization between CD3+ T cells with (left panel) tumor or (right panel) stromal cells. The tumors were then assigned to M-H low or high groups using the percentile cut-offs (represented by horizontal axis). Univariate Cox PH regression models were used to test for prognostic significance associated with tumors showing high versus low co-localization. Vertical axis indicates logarithmic transformed false discovery rate (FDR) values adjusted for the 13 cut-offs. The red dotted lines mark FDR = 0.05. Two combinations harbored significant associations (FDR ≤ 0.05) with colorectal cancer-specific survival for both discovery and validation subsets and are highlighted in red boxes with hazard ratios (HRs) and confidence intervals (CIs) labeled on top. ns for not significant, i.e., p > 0.05. (PDF) [file pcbi.1012707.s005.pdf]

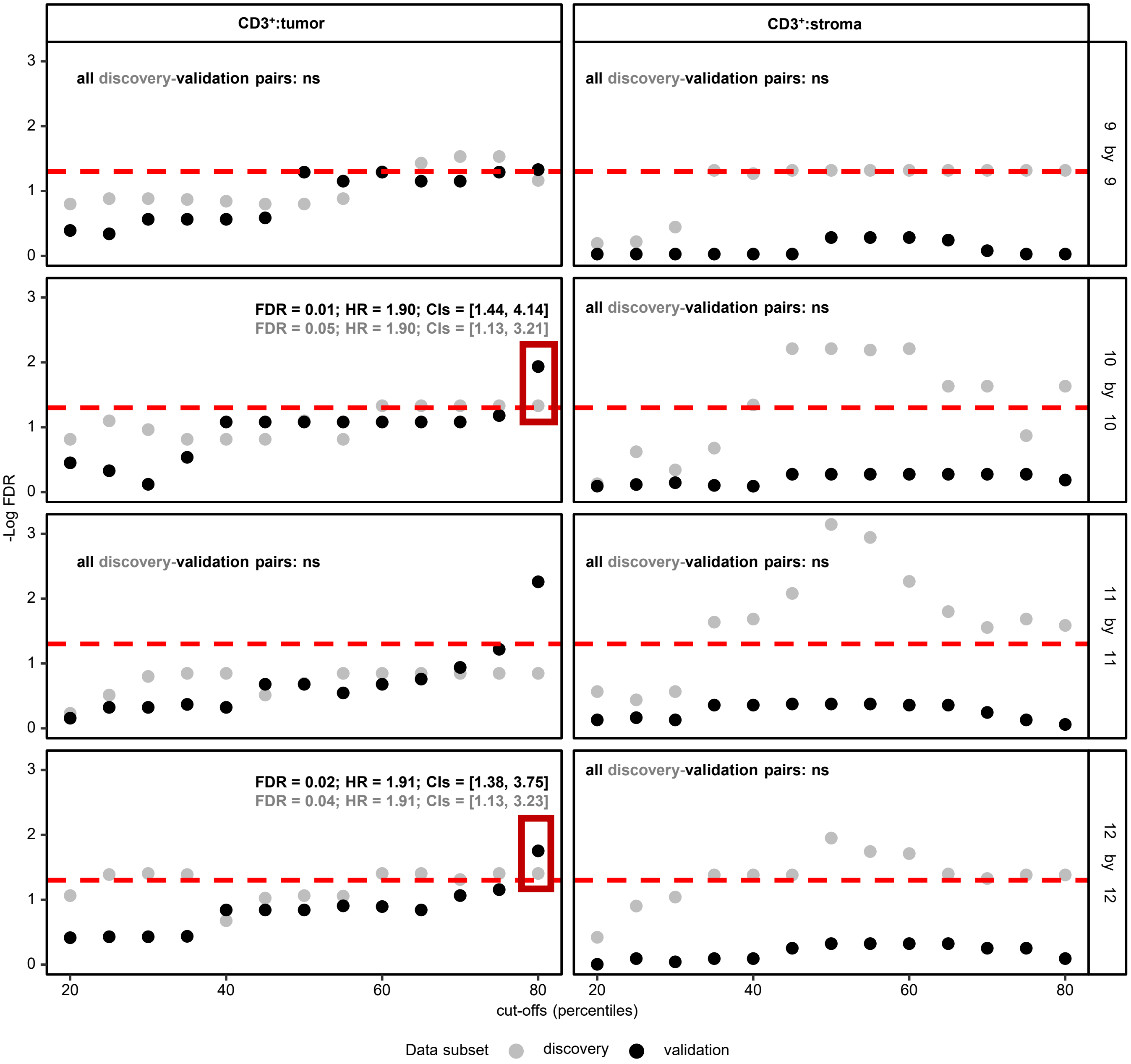

S5 Figure. Morisita-Horn (M-H) analysis using CD3$^+$ T cells. M-H index was first computed using 4.5-by-4.5, 5-by-5, 5.5-by-5.5, and 6-by-6 μm rectangular grids, measuring the degree of co-localization between CD3$^+$ T cells with (left panel) tumor or (right panel) stromal cells. The tumors were then assigned to M-H low or high groups using the percentile cut-offs (represented by horizontal axis). Univariate Cox PH regression models were used to test for prognostic significance associated with tumors showing high versus low co-localization. Vertical axis indicates logarithmic transformed false discovery rate (FDR) values adjusted for the 13 cut-offs. The red dotted lines mark FDR = 0.05. Two combinations harbored significant associations (FDR ≤ 0.05) with colorectal cancer-specific survival for both discovery and validation subsets and are highlighted in red boxes with hazard ratios (HRs) and confidence intervals (CIs) labeled on top. ns for not significant i.e., $p > 0.05$.
