# Supplementary material for: Tumor-immune partitioning and clustering algorithm for identifying tumor-immune cell spatial interaction signatures within the tumor microenvironment
Source: PLoS Comput Biol. 2025 Feb 18;21(2):e1012707. doi: 10.1371/journal.pcbi.1012707 (PMC11849983; doi:10.1371/journal.pcbi.1012707)
Supplement: S6 Fig — Prognostic performance evaluation of M-H subtypes derived from CD3+ T cells. At the two optimal grid sizes (5-by-5 and 6-by-6 μm, see S5 Fig for full details), both using a cut-off = 0.8 for dichotomizing tumors into low and high subtypes, both M-H solutions showed (a) confounding effects of overall CD3+ T-cell densities and (b) significant prognostic associations based on Kaplan-Meier estimates and log-rank test where P-values < 0.05. (PDF) [file pcbi.1012707.s006.pdf]

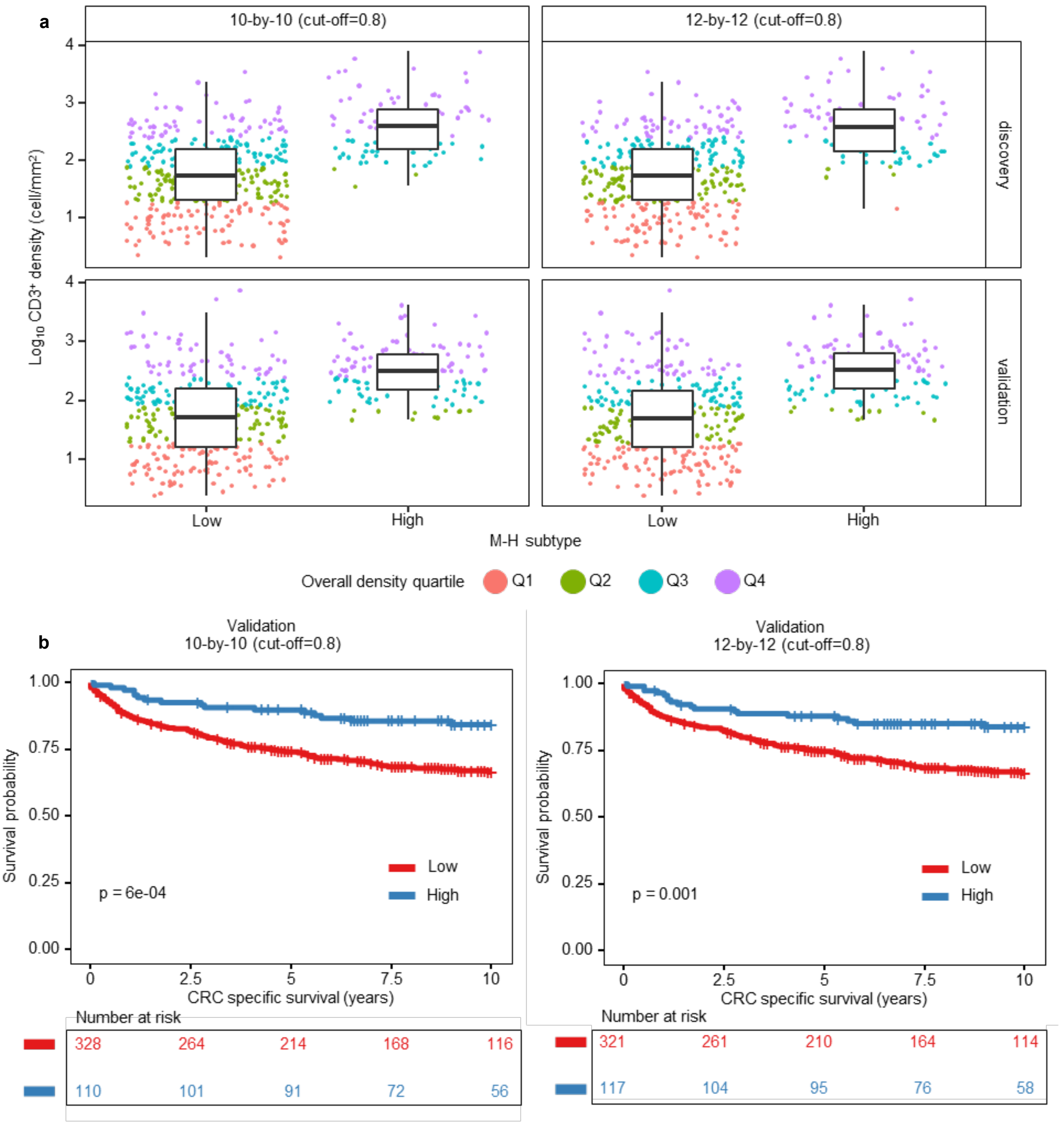

S6 Figure. Prognostic performance evaluation of M-H subtypes derived from $CD3^+$ T cells. At the two optimal grid sizes (5-by-5 and 6-by-6 μm, see **Fig. S5** for full details), both using a cut-off = 0.8 for dichotomizing tumors into low and high subtypes, both M-H solutions showed (**a**) confounding effects of overall $CD3^+$ T-cell densities and (**b**) significant prognostic associations based on Kaplan-Meier estimates and log-rank test where P-values < 0.05.
