# Supplementary material for: Tumor-immune partitioning and clustering algorithm for identifying tumor-immune cell spatial interaction signatures within the tumor microenvironment
Source: PLoS Comput Biol. 2025 Feb 18;21(2):e1012707. doi: 10.1371/journal.pcbi.1012707 (PMC11849983; doi:10.1371/journal.pcbi.1012707)
Supplement: S7 Fig — Performance evaluation of G-cross subtypes identified using CD3+ T cells. G-cross area under the curve (AUC), based (left panel) overall tissue regions and (right panel) stromal regions, was measured at r ≤ 20 μm and tumors were grouped into quartile categories of AUC. (a) Both analyses showed a significant confounding effect for overall CD3+ T cell density. (b) Only subtypes identified using CD3+ T cells in the overall tissue region showed prognostic significance value based on Kaplan-Meier estimates and the log-rank test. (PDF) [file pcbi.1012707.s007.pdf]

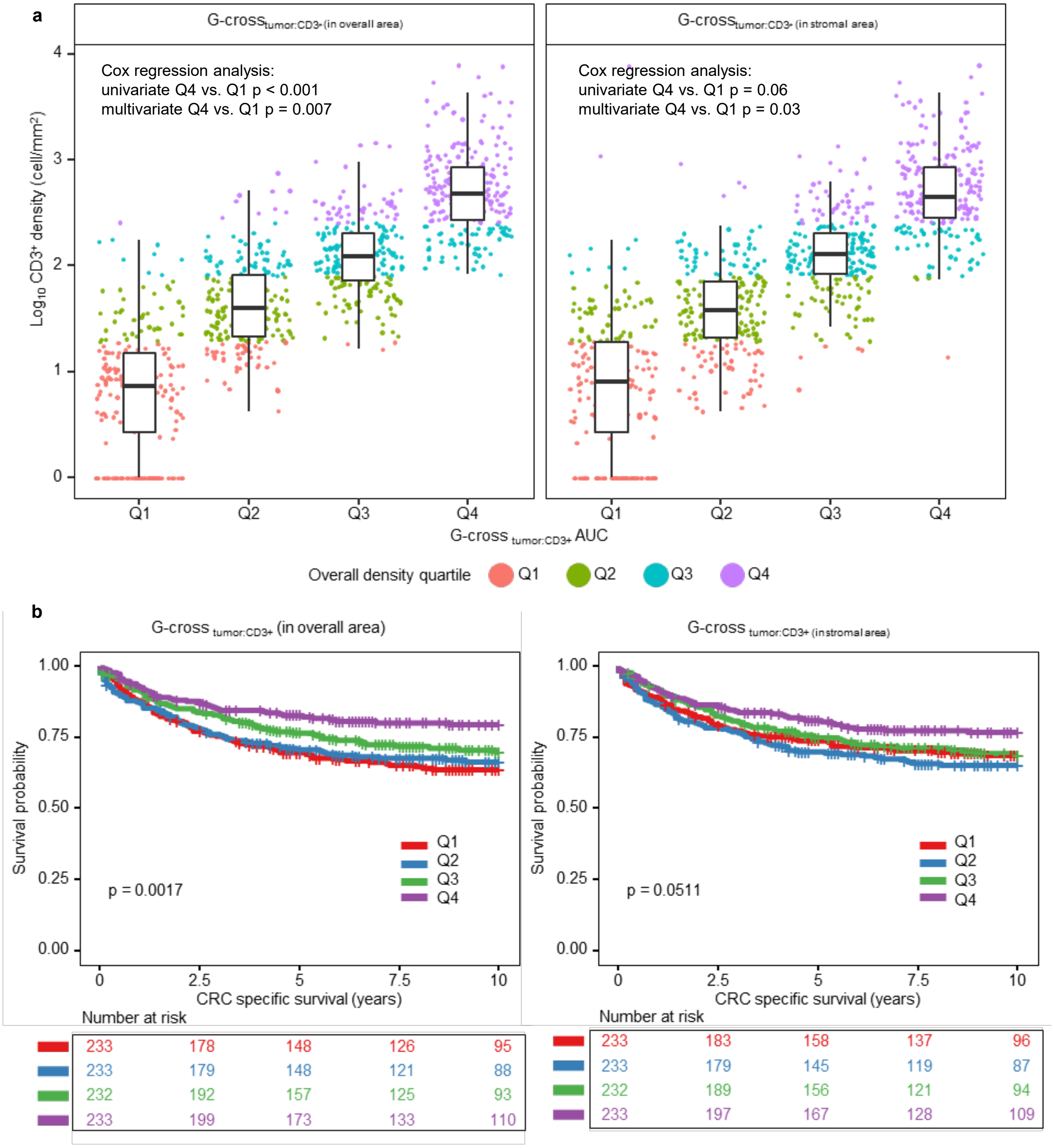

S7 Figure. Performance evaluation of G-cross subtypes identified using CD3⁺ T cells. G-cross area under the curve (AUC), based (left panel) overall tissue regions and (right panel) stromal regions, was measured at $r \leq 20$ μm and tumors were grouped into quartile categories of AUC. (**a**) Both analyses showed a significant confounding effect for overall CD3⁺ T cell density. (**b**) Only subtypes identified using CD3⁺ T cells in the overall tissue region showed prognostic significance value based on Kaplan-Meier estimates and the log-rank test.
