# Supplementary material for: Tumor-immune partitioning and clustering algorithm for identifying tumor-immune cell spatial interaction signatures within the tumor microenvironment
Source: PLoS Comput Biol. 2025 Feb 18;21(2):e1012707. doi: 10.1371/journal.pcbi.1012707 (PMC11849983; doi:10.1371/journal.pcbi.1012707)
Supplement: S8 Fig — Performance evaluation of L-cross subtypes identified using CD3+ T cell. L-cross area under the curve (AUC), based (left panel) overall tissue regions and (right panel) stromal regions, was measured at r ≤ 20 μm and tumors were grouped into quartile categories. (a) Both of these subtypes showed modest confounding due to overall CD3+ T cell densities and (b) no significant prognostic utility as assessed by Kaplan-Meier estimates and the log-rank test. (PDF) [file pcbi.1012707.s008.pdf]

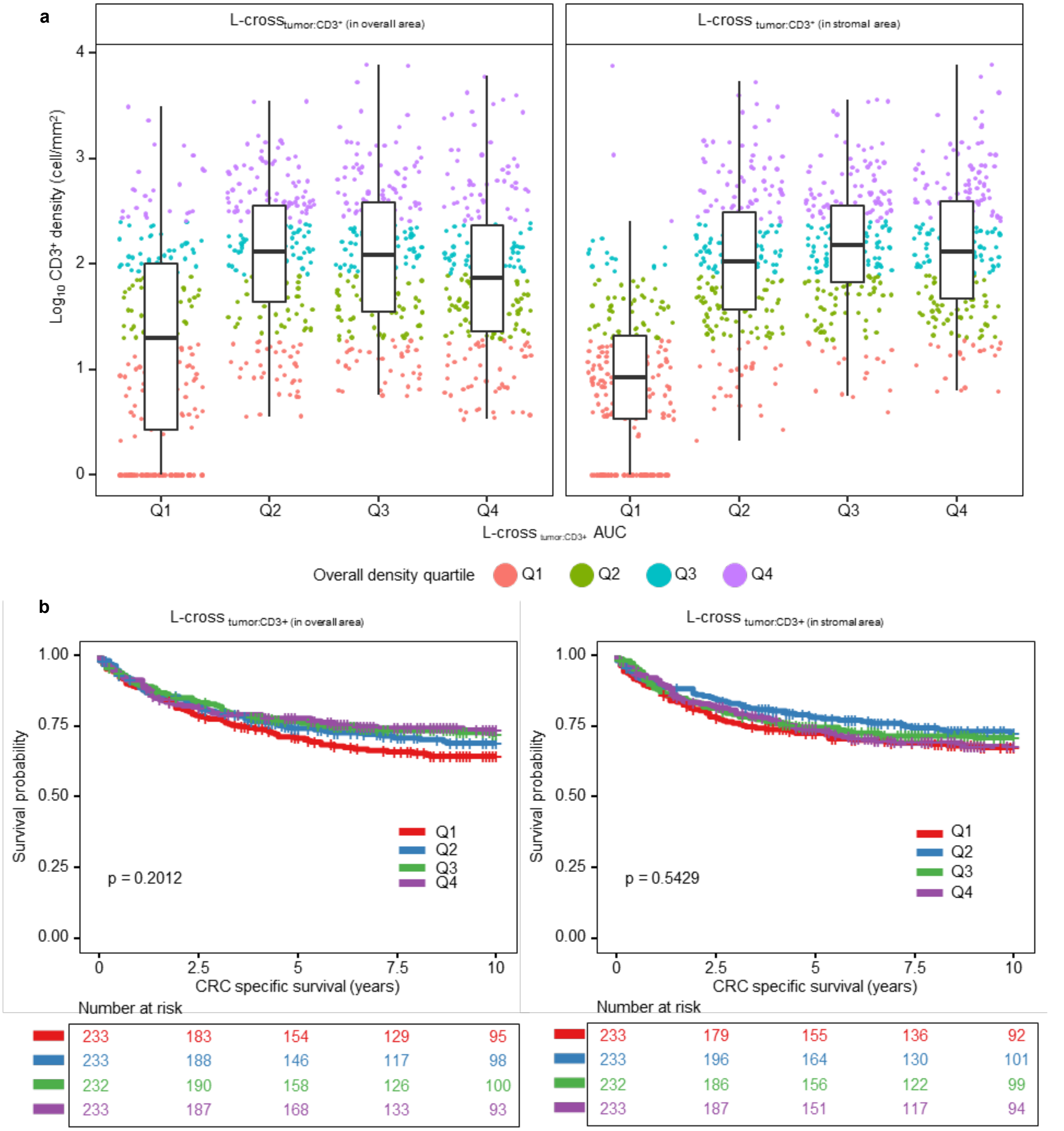

S8 Figure. Performance evaluation of L-cross subtypes identified using CD3+ T cell. L-cross area under the curve (AUC), based (left panel) overall tissue regions and (right panel) stromal regions, was measured at $r \leq 20$ μm and tumors were grouped into quartile categories. (**a**) Both of these subtypes showed modest confounding due to overall CD3+ T cell densities and (**b**) no significant prognostic utility as assessed by Kaplan-Meier estimates and the log-rank test.
