# Supplementary material for: Tumor-immune partitioning and clustering algorithm for identifying tumor-immune cell spatial interaction signatures within the tumor microenvironment
Source: PLoS Comput Biol. 2025 Feb 18;21(2):e1012707. doi: 10.1371/journal.pcbi.1012707 (PMC11849983; doi:10.1371/journal.pcbi.1012707)
Supplement: S9 Fig — Heat-map summarizes the association between molecular and pathologic features and TIPC spatial subtypes derived using CD3+ T cells. Extended Cochran–Armitage method was used to test the association significance between molecular and pathologic features (ordered variables) and TIPC spatial subtypes (unordered variable). Horizontal axis indicates cluster names: cold, stroma-rich (CSR), cold, tumor-rich (CTR), hot, tumor-centric clustering (HTCC), hot and disperse (HD), hot, stroma-centric clustering (HTCC), and hot and clustered (HC). (PDF) [file pcbi.1012707.s009.pdf]

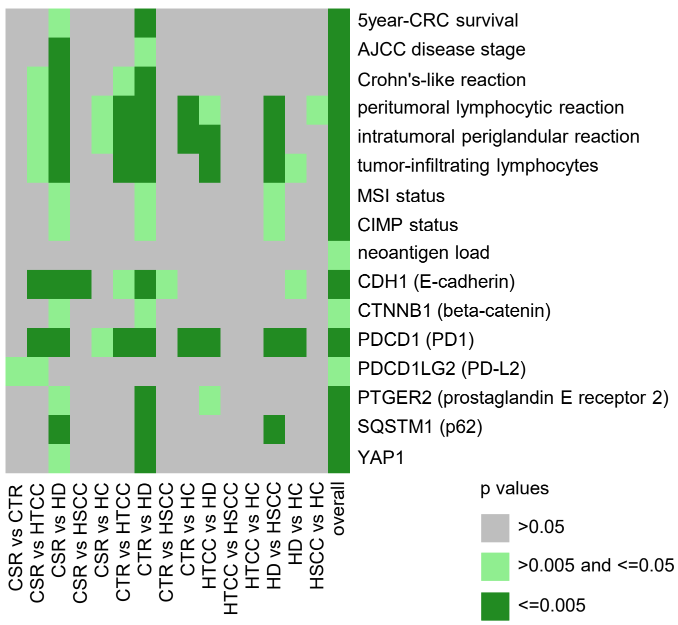

S9 Figure. Heat-map summarizes the association between molecular and pathologic features and TIPC spatial subtypes derived using CD3^{+} T cells. Extended Cochran–Armitage method was used to test the association significance between molecular and pathologic features (ordered variables) and TIPC spatial subtypes (unordered variable). Horizontal axis indicates cluster names: cold, stroma-rich (CSR), cold, tumor-rich (CTR), hot, tumor-centric clustering (HTCC), hot and disperse (HD), hot, stroma-centric clustering (HTCC), and hot and clustered (HC).
