# Supplementary material for: Tumor-immune partitioning and clustering algorithm for identifying tumor-immune cell spatial interaction signatures within the tumor microenvironment
Source: PLoS Comput Biol. 2025 Feb 18;21(2):e1012707. doi: 10.1371/journal.pcbi.1012707 (PMC11849983; doi:10.1371/journal.pcbi.1012707)
Supplement: S10 Fig — Survival analysis of TIPC subtypes derived from three different immune cell types, cytotoxic memory T cells (CD3+CD8+CD45RO+), eosinophils, and neutrophils in Nurses’ Health Study/Health Professionals Follow-up Study CRC datasets [24,25]. Kaplan-Meier and log-rank test show that the TIPC spatial subtypes of, (a) cytotoxic memory T cells (CD3+CD8+CD45RO+), (b) eosinophils, and (c) neutrophils, were significantly associated with colorectal cancer-specific survival. Abbreviations, CSR = cold, stroma-rich, CTR = cold, tumor-rich, HD = hot and disperse, HTCC = hot, tumor-centric clustering, HSCC = hot, stroma-centric clustering, HC = hot and clustered, HCTR = hot and clustered, tumor-rich, and HCSR = hot and clustered, stroma-rich. (PDF) [file pcbi.1012707.s010.pdf]

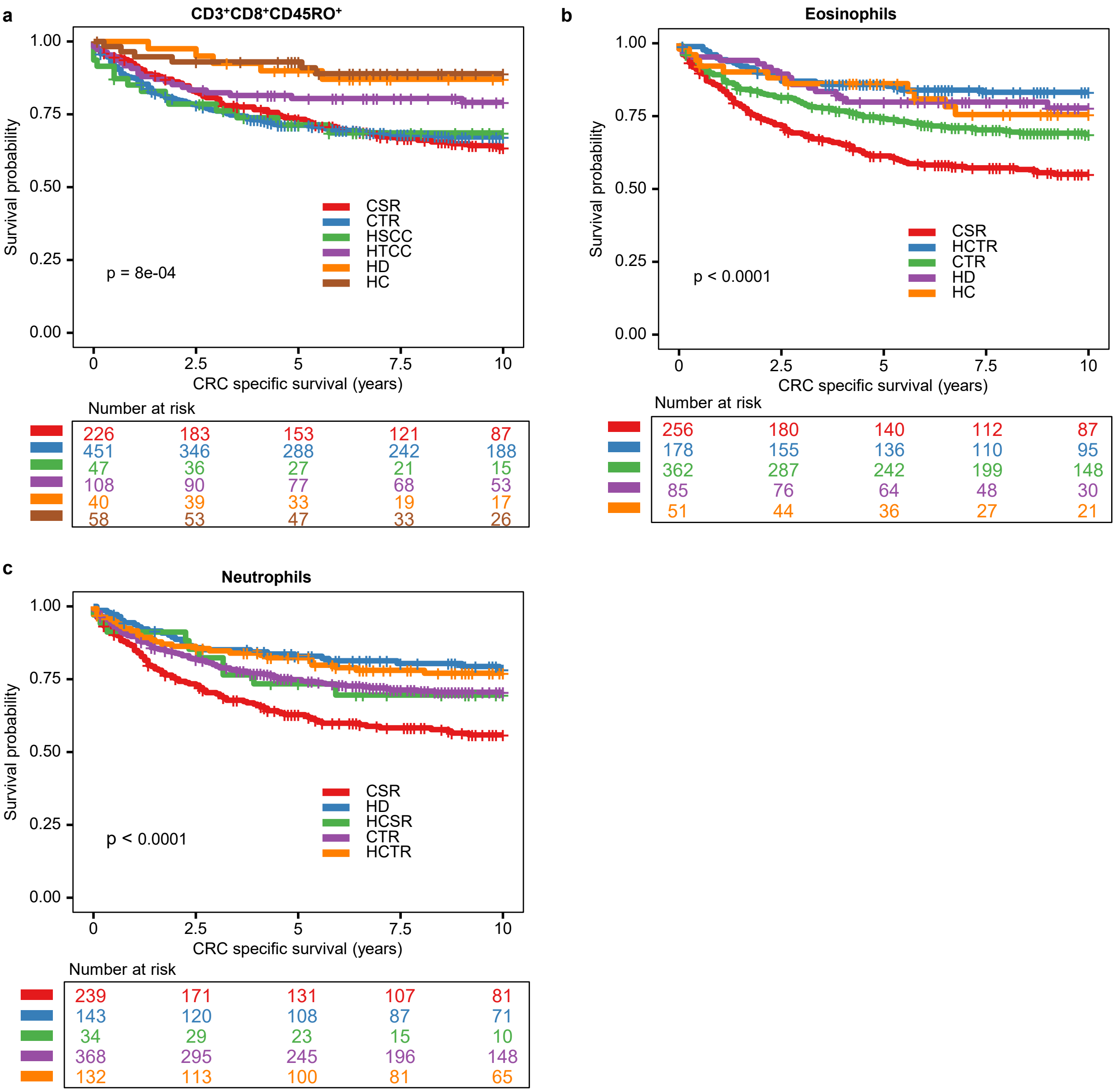

Figure S10. Survival analysis of TIPC subtypes derived from three different immune cell types, cytotoxic memory T cells (CD3$^+$CD8$^+$CD45RO$^+$), eosinophils, and neutrophils in Nurses' Health Study/Health Professionals Follow-up Study CRC datasets(24, 25). Kaplan-Meier and log-rank test show that the TIPC spatial subtypes of, (**a**) cytotoxic memory T cells (CD3$^+$CD8$^+$CD45RO$^+$), (**b**) eosinophils, and (**c**) neutrophils, were significantly associated with colorectal cancer-specific survival. Abbreviations, CSR = cold, stroma-rich, CTR = cold, tumor-rich, HD = hot and disperse, HTCC = hot, tumor-centric clustering, HSCC = hot, stroma-centric clustering, HC = hot and clustered, HCTR = hot and clustered, tumor-rich, and HCSR = hot and clustered, stroma-rich.
