# Supplementary material for: Tumor-immune partitioning and clustering algorithm for identifying tumor-immune cell spatial interaction signatures within the tumor microenvironment
Source: PLoS Comput Biol. 2025 Feb 18;21(2):e1012707. doi: 10.1371/journal.pcbi.1012707 (PMC11849983; doi:10.1371/journal.pcbi.1012707)
Supplement: S12 Fig — Cox proportional hazards regression analysis based on TIPC spatial subtypes derived from cytotoxic memory T cells (CD3+CD8+CD45RO+), eosinophils, and neutrophils in NHS/ HPFS; multivariable Cox proportional hazards adjusted for clinicopathologic features and cell densities. Forest plots associated with the subtypes of, (a) cytotoxic memory T cells (CD3+CD8+CD45RO+), (b) eosinophils, and (c) neutrophils, depicting hazard ratios and 95% confidence intervals for both univariable and multivariable (adjusted for both clinicopathologic features and cell density). Abbreviations, CSR = cold, stroma-rich, CTR = cold, tumor-rich, HD = hot and disperse, HTCC = hot, tumor-centric clustering, HSCC = hot, stroma-centric clustering, HC = hot and clustered, HCTR = hot and clustered, tumor-rich, and HCSR = hot and clustered, stroma-rich. Symbols *** p < 0.001, ** p < 0.01, * p < 0.05, not significant (ns) p > 0.05. (PDF) [file pcbi.1012707.s012.pdf]

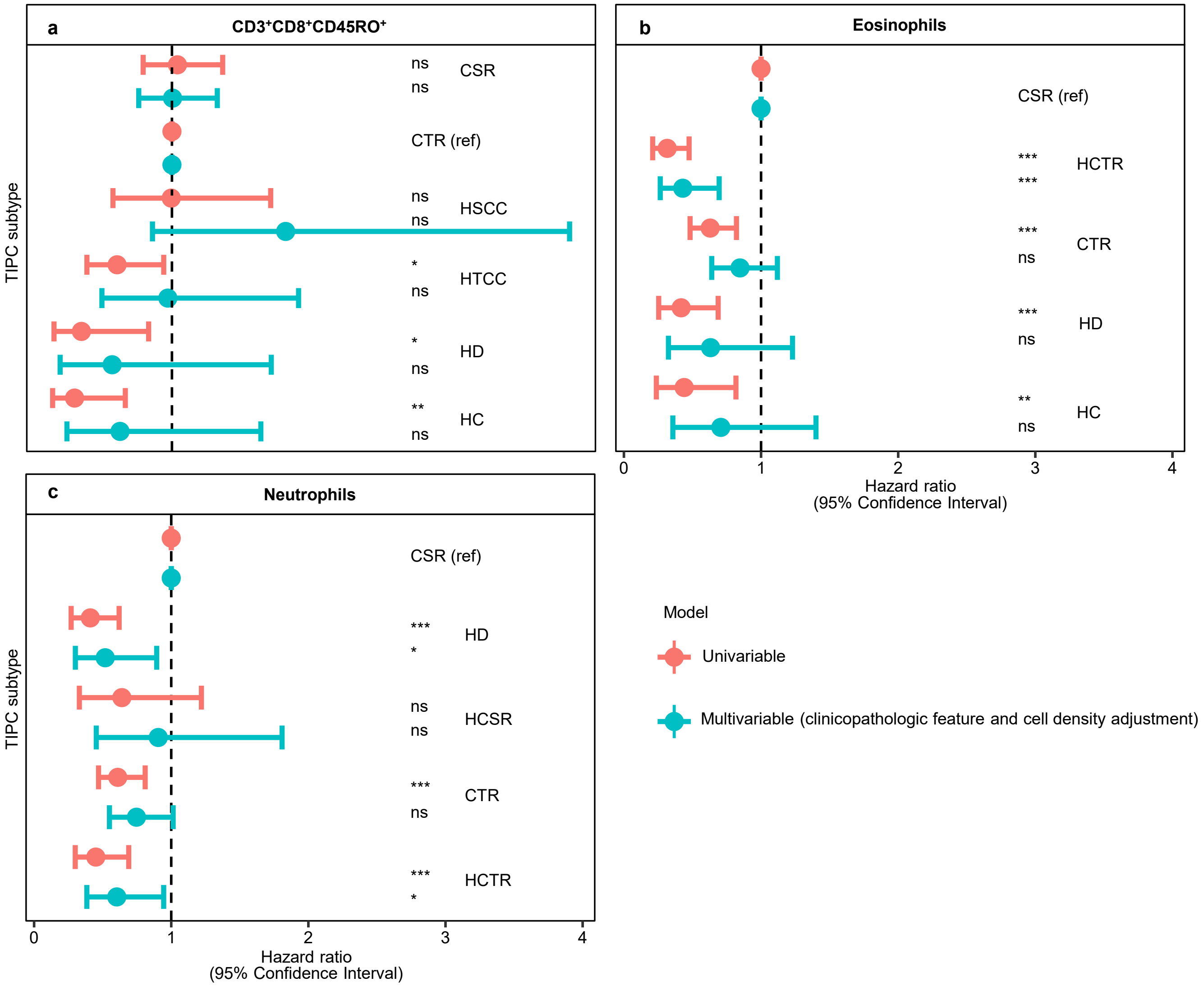

S12 Figure. Cox proportional hazards regression analysis based on TIPC spatial subtypes derived from cytotoxic memory T cells ($CD3^{+}CD8^{+}CD45RO^{+}$), eosinophils, and neutrophils in NHS/ HPFS; multivariable Cox proportional hazards adjusted for clinicopathologic features and cell densities. Forest plots associated with the subtypes of, (**a**) cytotoxic memory T cells ($CD3^{+}CD8^{+}CD45RO^{+}$), (**b**) eosinophils, and (**c**) neutrophils, depicting hazard ratios and 95% confidence intervals for both univariable and multivariable (adjusted for both clinicopathologic features and cell density). Abbreviations, CSR = cold, stroma-rich, CTR = cold, tumor-rich, HD = hot and disperse, HTCC = hot, tumor-centric clustering, HSCC = hot, stroma-centric clustering, HC = hot and clustered, HCTR = hot and clustered, tumor-rich, and HCSR = hot and clustered, stroma-rich. Symbols *** $p < 0.001$, ** $p < 0.01$, * $p < 0.05$, not significant (ns) $p > 0.05$.
