# Supplementary material for: Tumor-immune partitioning and clustering algorithm for identifying tumor-immune cell spatial interaction signatures within the tumor microenvironment
Source: PLoS Comput Biol. 2025 Feb 18;21(2):e1012707. doi: 10.1371/journal.pcbi.1012707 (PMC11849983; doi:10.1371/journal.pcbi.1012707)
Supplement: S14 Fig — Determination of optimal subregion size and input cluster number (k) for TIPC analysis using eosinophils. At individual subregion sizes of (a-c) 30, (d-f) 35, and (g-i) 40 μm, (a,d,g) cumulative distribution function (CDF) delta plots were first used to determine the minimum k for stable clustering (colored in red); (b,e,h) tracking plots revealed the relationship between granularity (high k yields high granularity) and cluster size (optimal k, marked by black boxes, were selected manually for ensuring a balance between granularity and statistical power). After excluding clusters comprising less than 30 tumors, (c,f,i) the major clusters with their spatial patterns represented by the six TIPC parameters were shown in the heat maps. The three TIPC solutions yielded similar spatial subtypes except that instead of HSCC subtype detected at sizes 25 and 30 μm, HC subtype was found at 35 μm, as (c,f) the former showed a relatively less coherent spatial profile, TIPC solution determined at 35 μm was used for downstream association analysis. Abbreviations: CSR = Cold, stroma-rich; CTR = Cold, tumor-rich; HD = Host and disperse; HSCC = Hot, stroma-centric clustering; HCTR = Host and clustered, tumor-rich. (PDF) [file pcbi.1012707.s014.pdf]

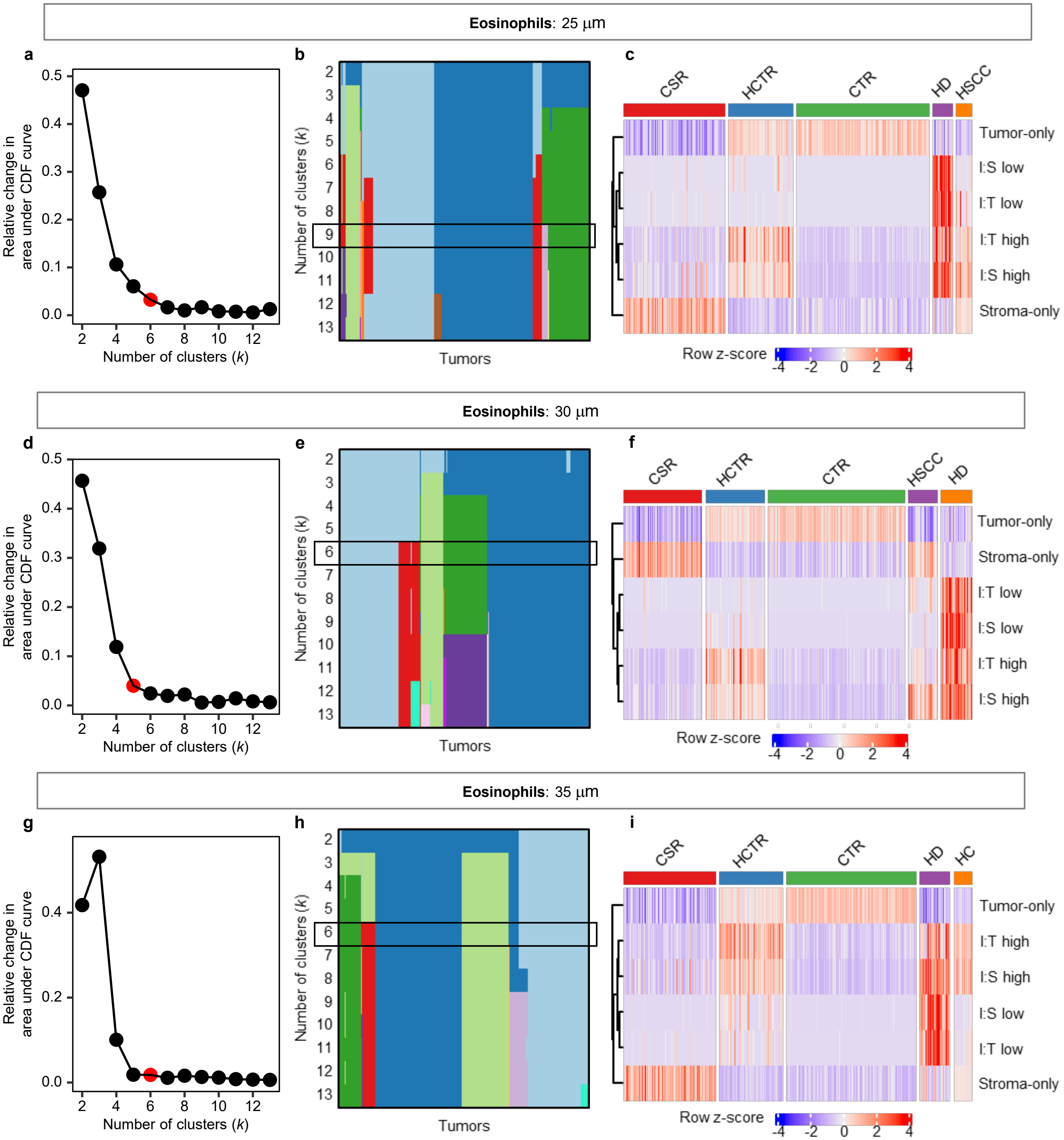

S14 Figure. Determination of optimal subregion size and input cluster number (*k*) for TIPC analysis using eosinophils. At individual subregion sizes of (**a**-**c**) 30, (**d**-**f**) 35, and (**g**-**i**) 40 µm, (**a**,**d**,**g**) cumulative distribution function (CDF) delta plots were first used to determine the minimum *k* for stable clustering (colored in red); (**b**,**e**,**h**) tracking plots revealed the relationship between granularity (high *k* yields high granularity) and cluster size (optimal *k*, marked by black boxes, were selected manually for ensuring a balance between granularity and statistical power). After excluding clusters comprising less than 30 tumors, (**c**,**f**,**i**) the major clusters with their spatial patterns represented by the six TIPC parameters were shown in the heat maps. The three TIPC solutions yielded similar spatial subtypes except that instead of HSCC subtype detected at sizes 25 and 30 µm, HC subtype was found at 35 µm, as (**c**,**f**) the former showed a relatively less coherent spatial profile, TIPC solution determined at 35 µm was used for downstream association analysis. Abbreviations: CSR = Cold, stroma-rich; CTR = Cold, tumor-rich; HD = Host and disperse; HSCC = Hot, stroma-centric clustering; HCTR = Host and clustered, tumor-rich.
