# Supplementary material for: Tumor-immune partitioning and clustering algorithm for identifying tumor-immune cell spatial interaction signatures within the tumor microenvironment
Source: PLoS Comput Biol. 2025 Feb 18;21(2):e1012707. doi: 10.1371/journal.pcbi.1012707 (PMC11849983; doi:10.1371/journal.pcbi.1012707)
Supplement: S16 Fig — Determination of optimal subregion size and input cluster number (k) for TIPC analysis using neutrophils. At individual subregion sizes of (a-c) 35, (d-f) 40, and (g-i) 50 μm, (a,d,g) cumulative distribution function (CDF) delta plots were first used to determine the minimum k for stable clustering (colored in red); (b,e,h) tracking plots revealed the relationship between granularity (high k yields high granularity) and cluster size (optimal k, marked by black boxes, were selected manually for ensuring a balance between granularity and statistical power). After excluding clusters comprising less than 30 tumors, (c,f,i) the major clusters with their spatial patterns represented by the six TIPC parameters were shown in the heat maps. Similar spatial subtypes were obtained using subregion size across 35-50 μm, except that instead of HCSR subtype detected at both sizes 35 and 50 μm (more robust), HSCC subtype was found at 40 μm, hence, TIPC solution determined at 35 μm (alternatively, 50 μ m could also be used) was used for downstream association analysis. Abbreviations: CSR = Cold, stroma-rich; CTR = Cold, tumor-rich; HD = Host and disperse; HSCC = Hot, stroma-centric clustering; HCTR = Host and clustered, tumor-rich. (PDF) [file pcbi.1012707.s016.pdf]

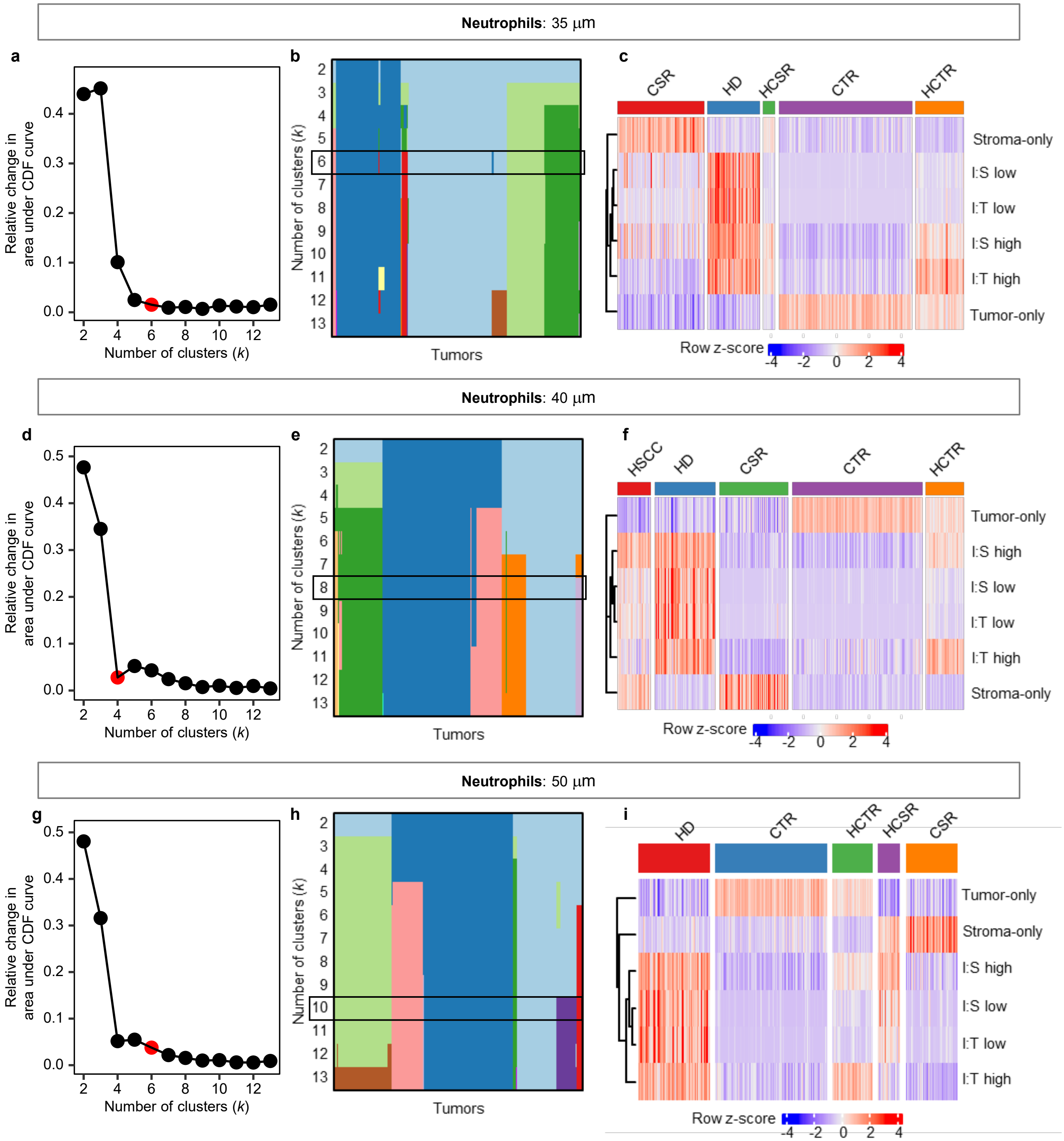

S16 Figure. Determination of optimal subregion size and input cluster number (*k*) for TIPC analysis using neutrophils. At individual subregion sizes of (**a**-**c**) 35, (**d**-**f**) 40, and (**g**-**i**) 50 μm, (**a**,**d**,**g**) cumulative distribution function (CDF) delta plots were first used to determine the minimum *k* for stable clustering (colored in red); (**b**,**e**,**h**) tracking plots revealed the relationship between granularity (high *k* yields high granularity) and cluster size (optimal *k*, marked by black boxes, were selected manually for ensuring a balance between granularity and statistical power). After excluding clusters comprising less than 30 tumors, (**c**,**f**,**i**) the major clusters with their spatial patterns represented by the six TIPC parameters were shown in the heat maps. Similar spatial subtypes were obtained using subregion size across 35-50 μm, except that instead of HCSR subtype detected at both sizes 35 and 50 μm (more robust), HSCC subtype was found at 40 μm, hence, TIPC solution determined at 35 μm (alternatively, 50 μ m could also be used) was used for downstream association analysis. Abbreviations: CSR = Cold, stroma-rich; CTR = Cold, tumor-rich; HD = Host and disperse; HSCC = Hot, stroma-centric clustering; HCTR = Host and clustered, tumor-rich.
