# Supplementary material for: Tumor-immune partitioning and clustering algorithm for identifying tumor-immune cell spatial interaction signatures within the tumor microenvironment
Source: PLoS Comput Biol. 2025 Feb 18;21(2):e1012707. doi: 10.1371/journal.pcbi.1012707 (PMC11849983; doi:10.1371/journal.pcbi.1012707)
Supplement: S17 Fig — Performance evaluation on the effect of subregion sizes and input cluster number (k) on spatial subtype identification and prognostic significance, using CD3+ T cells. TIPC analysis was performed using subregion sizes in the range of 30-50 μm, at each of these subregion sizes, input cluster numbers in the range of 4-10 were tested whereby univariate Cox regression model was used to test the association significance of the resulting TIPC subtypes with colorectal cancer-specific survival; subtypes comprising <30 tumors were excluded. Vertical axis indicates CD3+ T cell density (cells/mm2) for TIPC subtype. Subtypes were ordered based on their mean CD3+ T cell density, from the lowest (reference cluster) on the left to highest on the right; symbol size reflects the relative cluster size. Abbreviations: CSR = Cold, stroma-rich; CTR = Cold, tumor-rich; HTCC = Hot, tumor-centric clustering; HD = Host and disperse; HSCC = Hot, stroma-centric clustering; HC = Hot and clustered; HR = hazard ratio. (PDF) [file pcbi.1012707.s017.pdf]

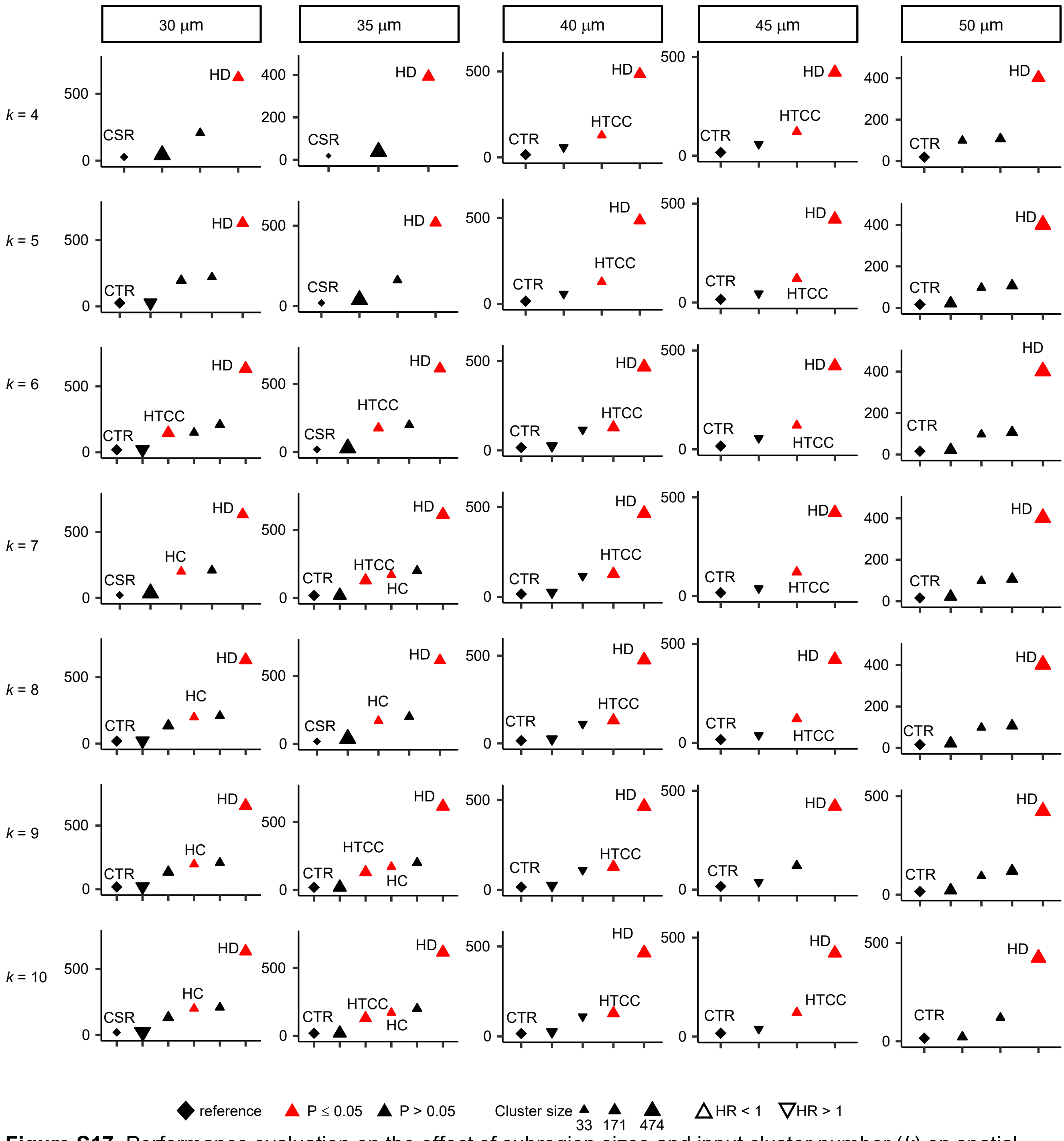

Figure S17. Performance evaluation on the effect of subregion sizes and input cluster number (*k*) on spatial subtype identification and prognostic significance, using $CD3^+$ T cells. TIPC analysis was performed using subregion sizes in the range of 30-50 µm, at each of these subregion sizes, input cluster numbers in the range of 4-10 were tested whereby univariate Cox regression model was used to test the association significance of the resulting TIPC subtypes with colorectal cancer-specific survival; subtypes comprising <30 tumors were excluded. Vertical axis indicates $CD3^+$ T cell density (cells/mm^2) for TIPC subtype. Subtypes were ordered based on their mean $CD3^+$ T cell density, from the lowest (reference cluster) on the left to highest on the right; symbol size reflects the relative cluster size. Abbreviations: CSR = Cold, stroma-rich; CTR = Cold, tumor-rich; HTCC = Hot, tumor-centric clustering; HD = Host and disperse; HSCC = Hot, stroma-centric clustering; HC = Hot and clustered; HR = hazard ratio.
