# Supplementary material for: Tumor-immune partitioning and clustering algorithm for identifying tumor-immune cell spatial interaction signatures within the tumor microenvironment
Source: PLoS Comput Biol. 2025 Feb 18;21(2):e1012707. doi: 10.1371/journal.pcbi.1012707 (PMC11849983; doi:10.1371/journal.pcbi.1012707)
Supplement: S18 Fig — Performance evaluation on the effect of subregion sizes and input cluster number (k) on spatial subtype identification and prognostic significance, using neutrophils. TIPC analysis was performed using subregion sizes in the range of 35-55 μm, at each of these subregion sizes, input cluster numbers in the range of 4-10 were tested whereby univariate Cox regression model was used to test the association significance of the resulting TIPC subtypes with CRC-specific survival (reference cluster: CSR); subtypes comprising <30 tumors were excluded. Vertical axis indicates neutrophils density (cells/mm2) for TIPC subtype. Subtypes were ordered based on their mean neutrophil density, from the lowest on the left to highest on the right; symbol size reflects the relative cluster size. Abbreviations: CTR = Cold, tumor-rich; HTCC = Hot, tumor-centric clustering; HD = Host and disperse; HSCC = Hot, stroma-centric clustering; HCTR = Host and clustered, tumor-rich; HR = hazard ratio. (PDF) [file pcbi.1012707.s018.pdf]

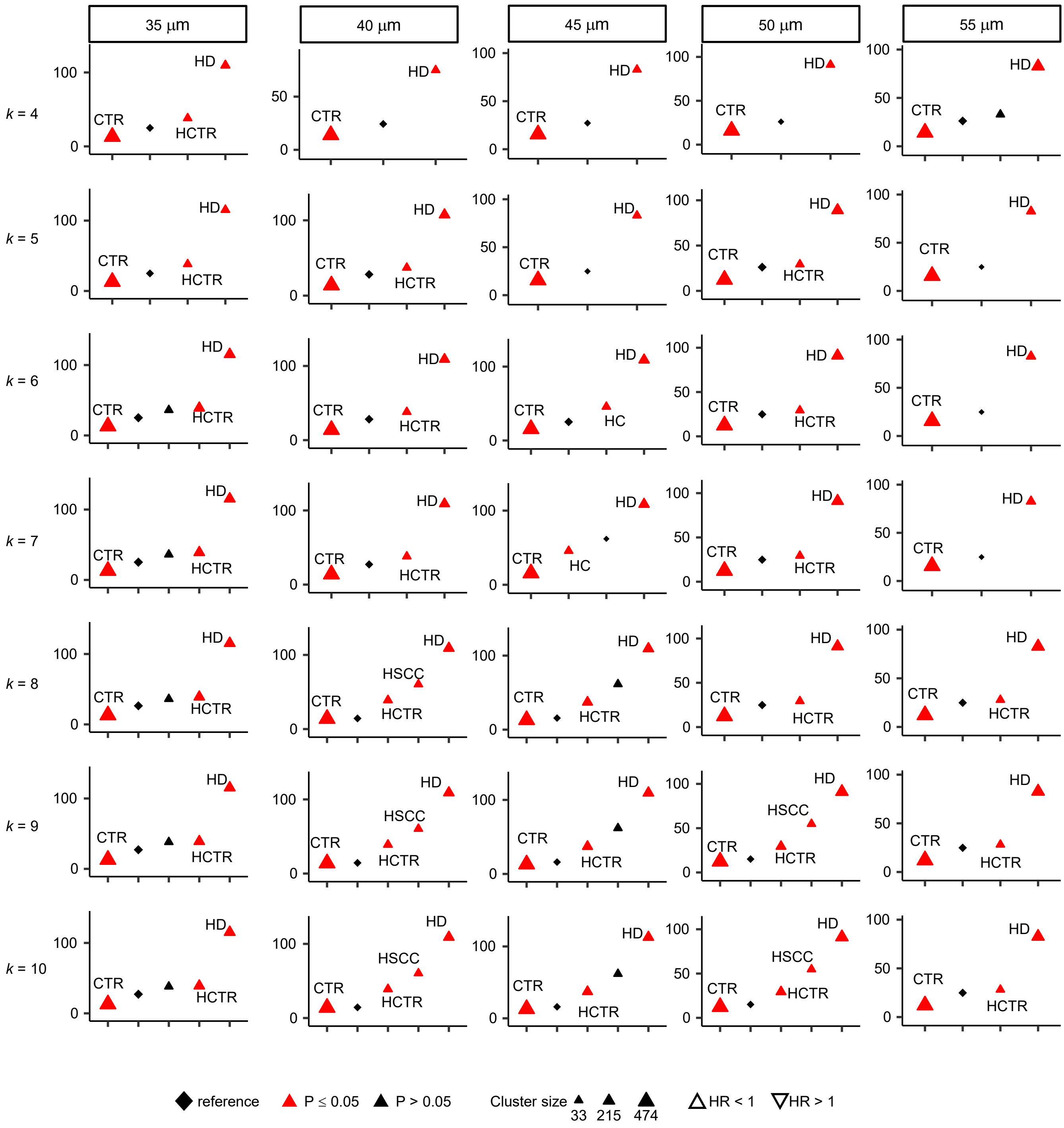

Figure S18. Performance evaluation on the effect of subregion sizes and input cluster number (*k*) on spatial subtype identification and prognostic significance, using neutrophils. TIPC analysis was performed using subregion sizes in the range of 35-55 μm, at each of these subregion sizes, input cluster numbers in the range of 4-10 were tested whereby univariate Cox regression model was used to test the association significance of the resulting TIPC subtypes with CRC-specific survival (reference cluster: CSR); subtypes comprising <30 tumors were excluded. Vertical axis indicates neutrophils density (cells/mm^2) for TIPC subtype. Subtypes were ordered based on their mean neutrophil density, from the lowest on the left to highest on the right; symbol size reflects the relative cluster size. Abbreviations: CTR = Cold, tumor-rich; HTCC = Hot, tumor-centric clustering; HD = Host and disperse; HSCC = Hot, stroma-centric clustering; HCTR = Host and clustered, tumor-rich; HR = hazard ratio.
