# Supplementary material for: Tumor-immune partitioning and clustering algorithm for identifying tumor-immune cell spatial interaction signatures within the tumor microenvironment
Source: PLoS Comput Biol. 2025 Feb 18;21(2):e1012707. doi: 10.1371/journal.pcbi.1012707 (PMC11849983; doi:10.1371/journal.pcbi.1012707)
Supplement: S19 Fig — Determination of optimal subregion size and cluster number (k) and the clustering results using TIPC package functions optimal_hexLen and optimal_k. Using (a-c) cytotoxic memory T cells, (d-f) eosinophils, and (g-i) neutrophils in CRC (NHS/HPFS), optimal_hexLen identified 70, 80, and 80 as the optimal subregion sizes, respectively. Then, optimal_k identified the shoulder points as the smallest cluster numbers—(a) 4, (d) 4, and (g) 4, respectively—to ensure stability, followed by selecting the largest stable k values—(b) 9, (e) 10, and (h) 10, respectively—to ensure granularity. After removing outlier clusters containing fewer than 30 samples, the resulting clusters were (c) 7, (f) 5, and (i) 5 spatial clusters, closely matching those generated by manual selection. Abbreviations: CSR = Cold, stroma-rich; CTR = Cold, tumor-rich; HD = Host and disperse; HC = hot and clustered; HTCC = hot, tumor-centric; HSCC = Hot, stroma-centric clustering; HCTR = Host and clustered, tumor-rich; HCSR = hot and clustered, stroma-rich. (PDF) [file pcbi.1012707.s019.pdf]

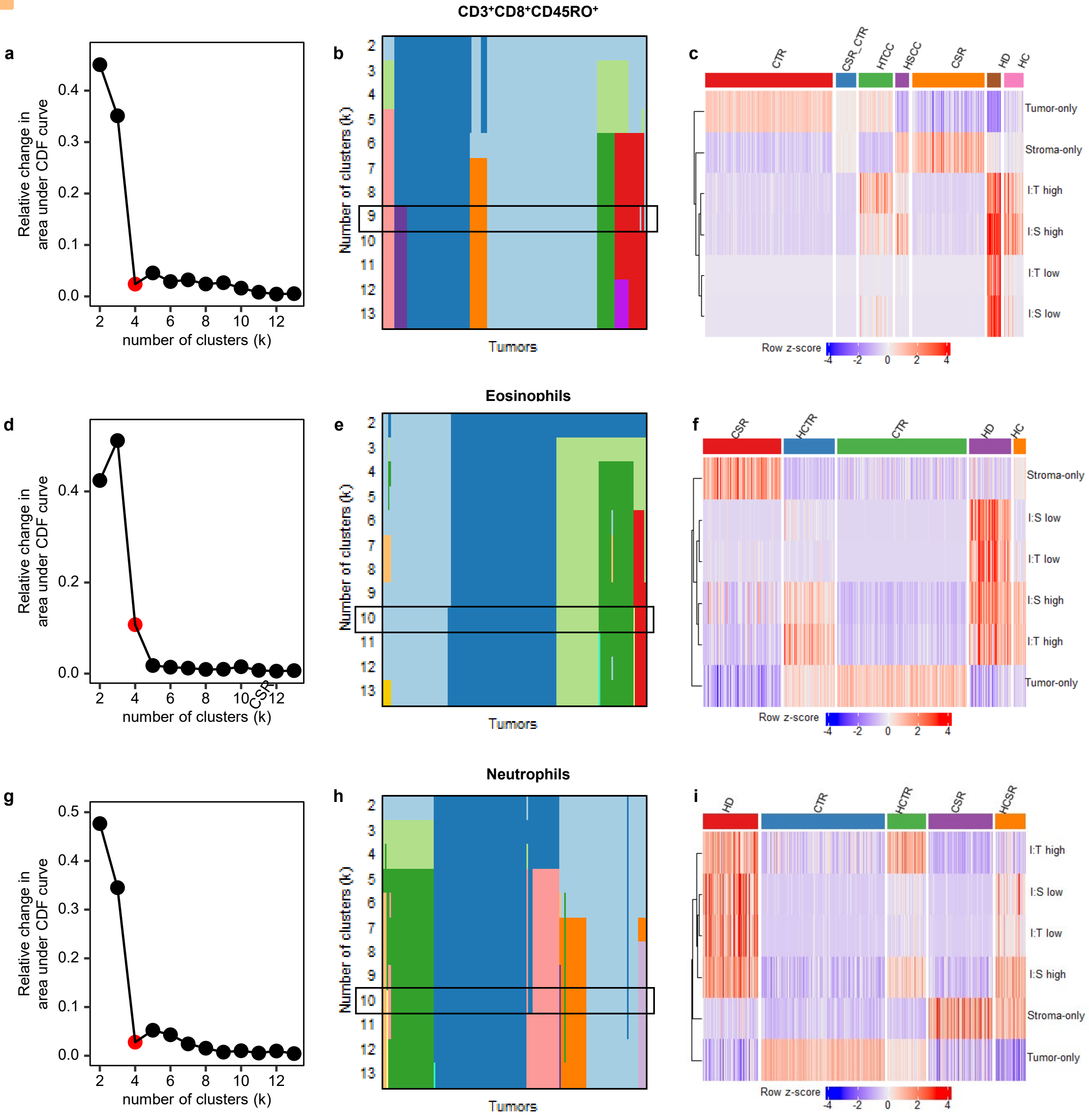

S19 Figure. Determination of optimal subregion size and cluster number (k) and the clustering results using TIPC package functions *optimal_hexLen* and *optimal_k*. Using (a-c) cytotoxic memory T cells, (d-f) eosinophils, and (g-i) neutrophils in CRC (NHS/HPFS), *optimal_hexLen* identified 70, 80, and 80 as the optimal subregion sizes, respectively. Then, *optimal_k* identified the shoulder points as the smallest cluster numbers—(a) 4, (d) 4, and (g) 4, respectively—to ensure stability, followed by selecting the largest stable k values—(b) 9, (e) 10, and (h) 10, respectively—to ensure granularity. After removing outlier clusters containing fewer than 30 samples, the resulting clusters were (c) 7, (f) 5, and (i) 5 spatial clusters, closely matching those generated by manual selection. Abbreviations: CSR = Cold, stroma-rich; CTR = Cold, tumor-rich; HD = Host and disperse; HC = hot and clustered; HTCC = hot, tumor-centric; HSCC = Hot, stroma-centric clustering; HCTR = Host and clustered, tumor-rich; HCSR = hot and clustered, stroma-rich.
