# Supplementary material for: Tumor-immune partitioning and clustering algorithm for identifying tumor-immune cell spatial interaction signatures within the tumor microenvironment
Source: PLoS Comput Biol. 2025 Feb 18;21(2):e1012707. doi: 10.1371/journal.pcbi.1012707 (PMC11849983; doi:10.1371/journal.pcbi.1012707)
Supplement: S20 Fig — Validation of the prognostic significance of TIPC subtypes which were first determined in Nurses’ Health Study/Health Professionals Follow-up Study (NHS/HPFS) CRC cohort[24,25] and later recapitulated in TCGA cohort, using eosinophils identified morphologically in H&E images. Among the five major TIPC subtypes determined in NHS/ HPFS cohort, three comprised of >30 tumors (i.e., CTR, HD, HCTR; see Fig 8). (a-b) Kaplan-Meier estimates associated with these TIPC subtypes of eosinophil subtypes harbored significant association with (a) disease-specific survival (DSS) and (b) progression-free intervals (PFI); (c) forest plot summarizes Cox regression analysis of tumor subtypes determined using cell density or nearest neighbor distance (NND) (see Fig 8 for the associations with overall survival). Symbols *** p < 0.001, ** p < 0.01, * p < 0.05, not significant (ns) p > 0.05. Abbreviations: CTR = Cold, tumor-rich; HD = Host and disperse; HCTR = Host and clustered, tumor-rich. (PDF) [file pcbi.1012707.s020.pdf]

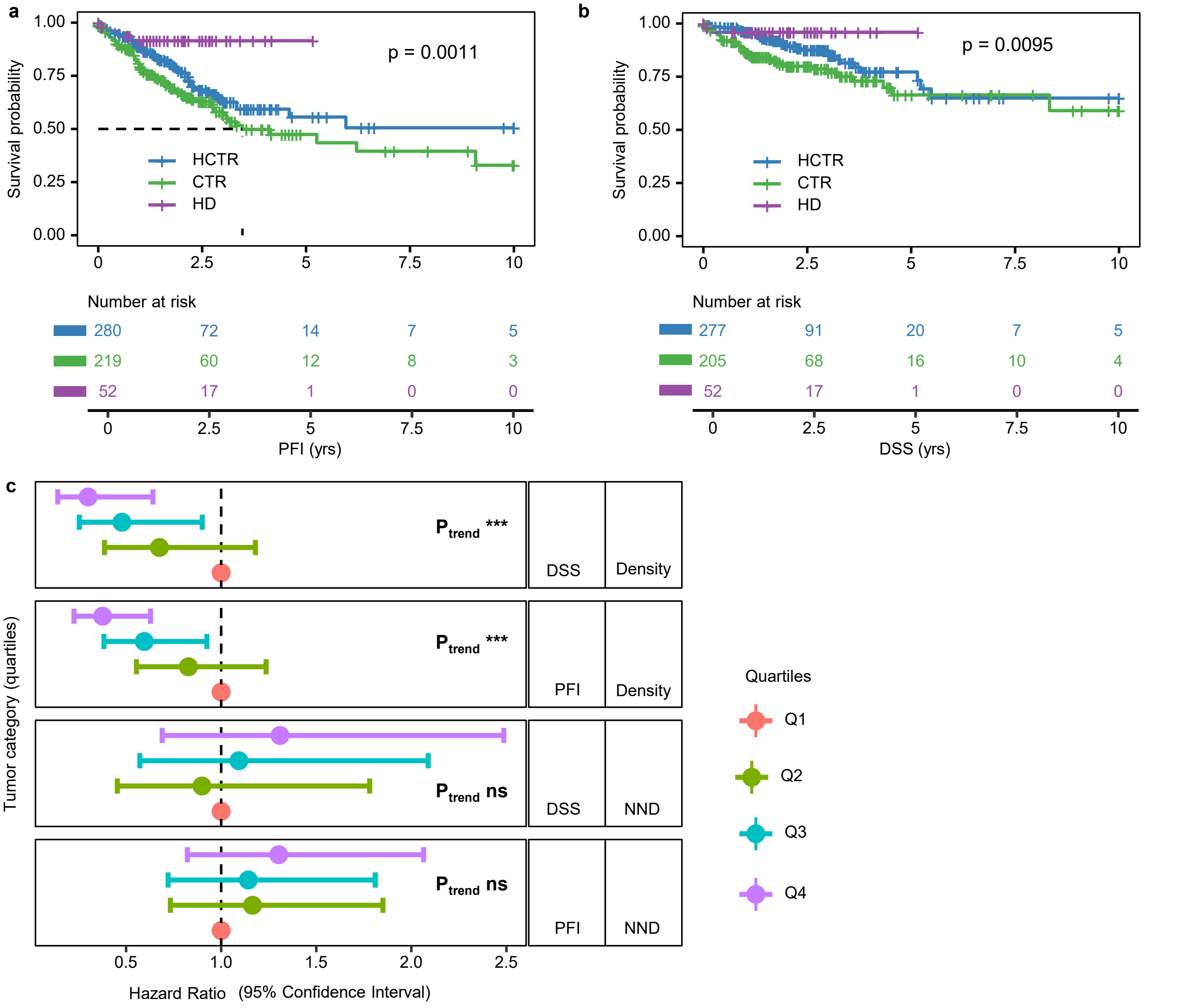

Figure S20. Validation of the prognostic significance of TIPC subtypes which were first determined in Nurses' Health Study/Health Professionals Follow-up Study (NHS/HPFS) CRC cohort(24, 25) and later recapitulated in TCGA cohort, using eosinophils identified morphologically in H&E images. Among the five major TIPC subtypes determined in NHS/ HPFS cohort, three comprised of >30 tumors (i.e., CTR, HD, HCTR; see Fig. 8). (a-b) Kaplan-Meier estimates associated with these TIPC subtypes of eosinophil subtypes harbored significant association with (a) disease-specific survival (DSS) and (b) progression-free intervals (PFI); (**c**) forest plot summarizes Cox regression analysis of tumor subtypes determined using cell density or nearest neighbor distance (NND) (see Fig. 8 for the associations with overall survival). Symbols *** p < 0.001, ** p < 0.01, * p < 0.05, not significant (ns) p > 0.05. Abbreviations: CTR = Cold, tumor-rich; HD = Host and disperse; HCTR = Host and clustered, tumor-rich.
