# Supplementary material for: Tumor-immune partitioning and clustering algorithm for identifying tumor-immune cell spatial interaction signatures within the tumor microenvironment
Source: PLoS Comput Biol. 2025 Feb 18;21(2):e1012707. doi: 10.1371/journal.pcbi.1012707 (PMC11849983; doi:10.1371/journal.pcbi.1012707)
Supplement: S21 Fig — Morisita-Horn (M-H) analysis using eosinophils identified in Nurses’ Health Study/Health Professionals Follow-up Study CRC cohorts [24,25]. M-H index was first computed using rectangular grid sizes of 4.5-by-4.5, 5-by-5, 5.5-by-5.5, and 6-by-6 μm, measuring the co-localization between eosinophils with stromal (left panel) or tumor cells (right panel). The tumors were then assigned to M-H low or high groups using the percentile cut-offs (represented by horizontal axis). Univariate Cox proportional hazards regression models were used to test for prognostic significance associated with tumors showing high (M-H high group) versus low (M-H low group) co-localization. Vertical axis indicates logarithmic transformed false discovery rate (FDR) values adjusted for the 13 cut-offs. The red dotted lines mark FDR = 0.05. Combinations harbored significant associations (FDR ≤ 0.05) with colorectal cancer-specific survival for both discovery and validation subsets and were highlighted in red boxes. (PDF) [file pcbi.1012707.s021.pdf]

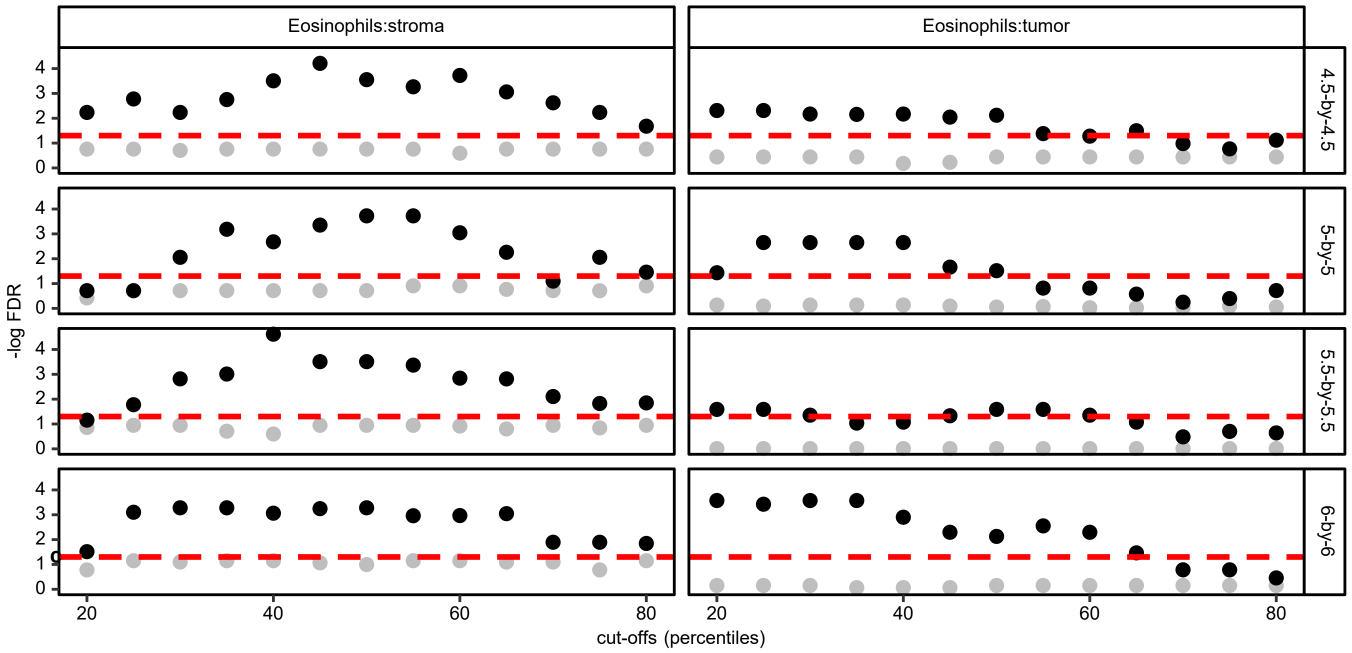

Figure S21. Morisita-Horn (M-H) analysis using eosinophils identified in Nurses' Health Study/Health Professionals Follow-up Study CRC cohorts(24, 25). M-H index was first computed using rectangular grid sizes of 4.5-by-4.5, 5-by-5, 5.5-by-5.5, and 6-by-6 μm, measuring the co-localization between eosinophils with stromal (left panel) or tumor cells (right panel). The tumors were then assigned to M-H low or high groups using the percentile cut-offs (represented by horizontal axis). Univariate Cox proportional hazards regression models were used to test for prognostic significance associated with tumors showing high (M-H high group) versus low (M-H low group) co-localization. Vertical axis indicates logarithmic transformed false discovery rate (FDR) values adjusted for the 13 cut-offs. The red dotted lines mark FDR = 0.05. Combinations harbored significant associations (FDR $\leq$ 0.05) with colorectal cancer-specific survival for both discovery and validation subsets and were highlighted in red boxes.
