# Supplementary material for: Tumor-immune partitioning and clustering algorithm for identifying tumor-immune cell spatial interaction signatures within the tumor microenvironment
Source: PLoS Comput Biol. 2025 Feb 18;21(2):e1012707. doi: 10.1371/journal.pcbi.1012707 (PMC11849983; doi:10.1371/journal.pcbi.1012707)
Supplement: S22 Fig — Comparison of the number of tumors in Morisita-Horn (M-H) low and high groups in Nurses’ Health Study/Health Professionals Follow-up Study (NHS/ HPFS) [24,25] and The Cancer Genome Atlas (TCGA) cohorts. M-H index was computed using rectangular grid sizes of 4.5-by-4.5, 5-by-5, 5.5-by-5.5, and 6-by-6 μm, measuring the co-localization between eosinophils with stromal (left panel) or tumor cells (right panel), in (a) NHS/HPFS and (b) TCGA cohorts, individually. Based on the percentiles (represented by horizontal axis) determined in (a) NHS/ HPFS cohorts, (b) TCGA cohort were divided into M-H low and high groups, where TCGA cohort showed an extremely skewed distribution with very few tumors assigned to the M-H low group across all the combinations of grid sizes and cut-offs. (PDF) [file pcbi.1012707.s022.pdf]

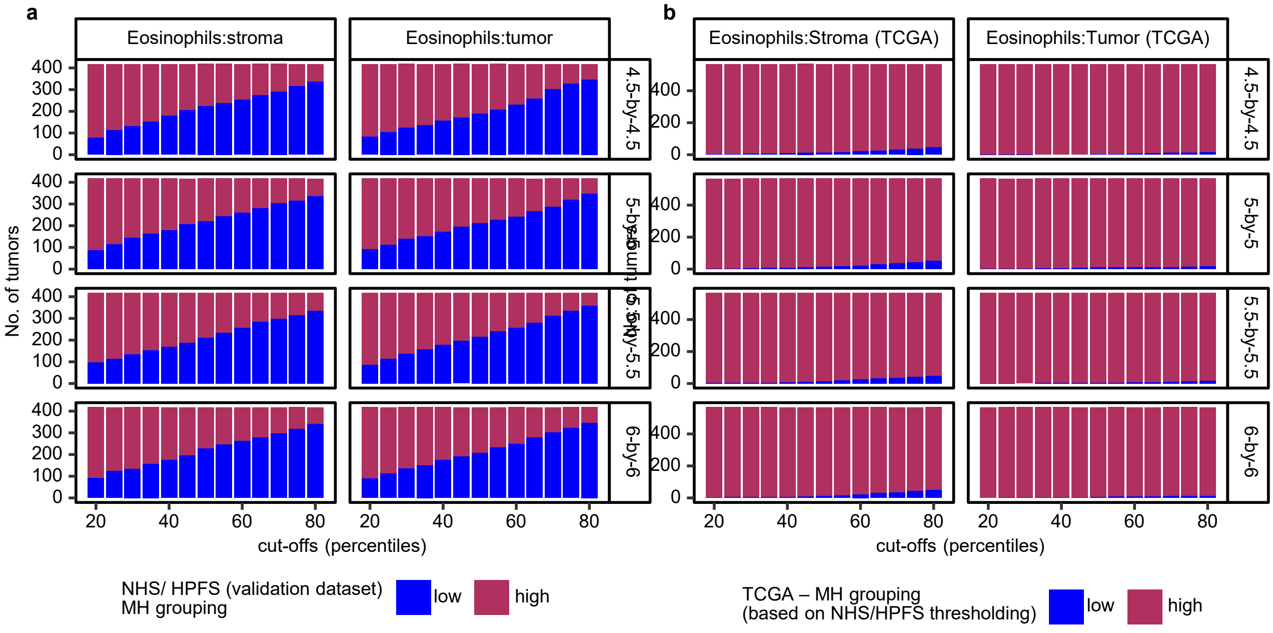

Figure S22. Comparison of the number of tumors in Morisita-Horn (M-H) low and high groups in Nurses' Health Study/Health Professionals Follow-up Study (NHS/ HPFS)(24, 25) and The Cancer Genome Atlas (TCGA) cohorts. M-H index was computed using rectangular grid sizes of 4.5-by-4.5, 5-by-5, 5.5-by-5.5, and 6-by-6 μm, measuring the co-localization between eosinophils with stromal (left panel) or tumor cells (right panel), in (**a**) NHS/HPFS and (**b**) TCGA cohorts, individually. Based on the percentiles (represented by horizontal axis) determined in (**a**) NHS/ HPFS cohorts, (**b**) TCGA cohort were divided into M-H low and high groups, where TCGA cohort showed an extremely skewed distribution with very few tumors assigned to the M-H low group across all the combinations of grid sizes and cut-offs.
