# Supplementary material for: Tumor-immune partitioning and clustering algorithm for identifying tumor-immune cell spatial interaction signatures within the tumor microenvironment
Source: PLoS Comput Biol. 2025 Feb 18;21(2):e1012707. doi: 10.1371/journal.pcbi.1012707 (PMC11849983; doi:10.1371/journal.pcbi.1012707)
Supplement: S1 Table — Definition of ‘spatial’ terminology. (PDF) [file pcbi.1012707.s023.pdf]

S1 Table. Definition of 'spatial' terminology.

Glossary of terms	Definition
Immune cell spatial distribution, organization or configuration	Cell locations in a Cartesian space for a collection of immune cells of interest in the tumor microenvironment
Tumor-immune spatial relationship	The spatial distribution of immune cells relative to the tumor and non-tumor (stromal) cells at a microscopic level.
Tumor-immune spatial pattern (abbreviated as spatial pattern)	Combinations of tumor-immune spatial relationships observed across an entire tissue region
TIPC spatial measures/ parameters	Six metrics characterizing the tumor-immune spatial relationship for a tumor microenvironment, capturing the degree of immune cell infiltration, predominance of tumor or stromal regions and degree of immune cell clustering or dispersion. Represented as normalized subregion counts in a six-element numeric vector. See Supplementary Table 2 for detailed metric definitions.
Spatial Point Pattern (SPP)	Dataset providing the observed spatial locations of objects.
