# Supplementary material for: Tumor-immune partitioning and clustering algorithm for identifying tumor-immune cell spatial interaction signatures within the tumor microenvironment
Source: PLoS Comput Biol. 2025 Feb 18;21(2):e1012707. doi: 10.1371/journal.pcbi.1012707 (PMC11849983; doi:10.1371/journal.pcbi.1012707)
Supplement: S2 Table — Definition of the six TIPC spatial parameters. (PDF) [file pcbi.1012707.s024.pdf]

S2 Table. Definition of the six TIPC spatial parameters.

**TIPC subregion classification**	**Definition**
Tumor only	Subregion containing only tumor cells
Immune-to-tumor low (I:T low)	Subregion containing both immune and tumor cells where the local immune to tumor ratio is smaller than the global ratio
Immune-to-tumor high (I:T high)	Subregion containing both immune and tumor cells where the local immune to tumor ratio is larger than the global ratio
Stroma-only	Subregion containing only stromal cells
Immune-to-stroma low (I:S low)	Subregion containing both immune and stromal cells where the local immune to stroma ratio is smaller than the global ratio
Immune-to-stroma high (I:S high)	Subregion containing both immune and stromal cells where the local immune to stroma ratio is larger than the global ratio
