# Supplementary material for: Tumor-immune partitioning and clustering algorithm for identifying tumor-immune cell spatial interaction signatures within the tumor microenvironment
Source: PLoS Comput Biol. 2025 Feb 18;21(2):e1012707. doi: 10.1371/journal.pcbi.1012707 (PMC11849983; doi:10.1371/journal.pcbi.1012707)
Supplement: S3 Table — Evaluation of the confounding effect of CD3+ T-cell density on tumor subtypes derived by existing spatial analysis methods. Multivariable Cox proportional hazards model included both the tumor subtypes identified by (a-b) the Morisita-Horn (M-H) index, (c) G-cross, and (d) L-cross functions and overall CD3+ T cell density (quartiles). M-H subtypes were generated using (a) 5-by-5 and (b) 6-by-6 μm grid sizes based on the validation dataset (see S5 Fig for details). HR, hazard ratio; CI, confidence interval. (PDF) [file pcbi.1012707.s025.pdf]

S3 Table. Evaluation of the confounding effect of $CD3^+$ T-cell density on tumor subtypes derived by existing spatial analysis methods. Multivariable Cox proportional hazards model included both the tumor subtypes identified by (a-b) the Morisita-Horn (M-H) index, (c) G-cross, and (d) L-cross functions and overall $CD3^+$ T cell density (quartiles). M-H subtypes were generated using (a) 5-by-5 and (b) 6-by-6 μm grid sizes based on the validation dataset (see Fig. S5 for details). HR, hazard ratio; CI, confidence interval.

(a)

		HR (95% CI)	P values
overall $CD3^+$ T-cell density	1st quartile	Reference	
	2nd quartile	1.03 (0.63-1.70)	0.909
	3rd quartile	0.98 (0.58-1.65)	0.941
	4th quartile	0.95 (0.54-1.66)	0.859
$Morisita\text{-}Horn_{tumor:CD3+T\ cell}$ (5-by-5 μm grid)	low	Reference	
	high	0.42 (0.24-0.75)	0.003

(b)

		HR	P values
overall $CD3^+$ T-cell density	1st quartile	Reference	
	2nd quartile	1.02 (0.62-1.67)	0.949
	3rd quartile	0.99 (0.59-1.67)	0.977
	4th quartile	0.94 (0.54-1.66)	0.840
$Morisita\text{-}Horn_{tumor:CD3+T\ cell}$ (6-by-6 μm grid)	low	Reference	
	high	0.45 (0.26-0.79)	0.005

(c)

		HR	P values
$CD3^+$ T-cell density (in stroma)	1st quartile	Reference	
	2nd quartile	0.80 (0.52-1.22)	0.293
	3rd quartile	0.50 (0.30-0.84)	0.008
	4th quartile	0.45 (0.25-0.80)	0.007
$G\text{-}cross_{tumor:CD3+T\ cell\ (in\ stroma)}$	1st quartile	Reference	
	2nd quartile	1.48 (0.97-2.26)	0.07
	3rd quartile	1.61 (0.98-2.67)	0.061
	4th quartile	1.43 (0.78-2.60)	0.244

(d)

		HR	P values
$CD3^+$ T-cell density (in stroma)	1st quartile	Reference	
	2nd quartile	0.93 (0.62-1.38)	0.707
	3rd quartile	0.58 (0.37-0.92)	0.021
	4th quartile	0.50 (0.32-0.81)	0.004
$L\text{-}cross_{tumor:CD3+T\ cell\ (in\ stroma)}$	1st quartile	Reference	
	2nd quartile	1.06 (0.69-1.64)	0.78
	3rd quartile	1.33 (0.84-2.10)	0.226
	4th quartile	1.35 (0.87-2.09)	0.184
