# Supplementary material for: Tumor-immune partitioning and clustering algorithm for identifying tumor-immune cell spatial interaction signatures within the tumor microenvironment
Source: PLoS Comput Biol. 2025 Feb 18;21(2):e1012707. doi: 10.1371/journal.pcbi.1012707 (PMC11849983; doi:10.1371/journal.pcbi.1012707)
Supplement: S4 Table — Comparison of prognostic significance between tumor subtypes derived by TIPC and other existing methods. Multivariable Cox proportional hazards model included both the tumor subtypes identified by TIPC and (a) CD3+ T cell density quartiles, (b) Morisita-Horntumor:CD3+T cell index quartiles (using a 5-by-5 μm grid and 80th percentile dichotomization cut-off), (c) G-crosstumor:CD3+T cell (in stroma) AUC quartiles (r < 20 μm), and (d) L-crosstumor:CD3+T cell (in stroma) AUC quartiles (r < 20 μm). Abbreviations: CSR = Cold, stroma-rich; CTR = Cold, tumor-rich; HTCC = Hot, tumor-centric clustering; HD = Host and disperse; HSCC = Hot, stroma-centric clustering; HC = Hot and clustered; HCTR = Host and clustered, tumor-rich; HCSR = Hot and clustered, stroma-rich.; HR = hazard ratio; CI = confidence interval. (PDF) [file pcbi.1012707.s026.pdf]

S4 Table. Comparison of prognostic significance between tumor subtypes derived by TIPC and other existing methods. Multivariable Cox proportional hazards model included both the tumor subtypes identified by TIPC and (a) $CD3^+$ T cell density quartiles, (b) Morisita-Horn$_{tumor:CD3+T\ cell}$ index quartiles (using a 5-by-5 μm grid and 80th percentile dichotomization cut-off), (c) G-cross$_{tumor:CD3+T\ cell\ (in\ stroma)}$ AUC quartiles (r < 20 μm), and (d) L-cross$_{tumor:CD3+T\ cell\ (in\ stroma)}$ AUC quartiles (r < 20 μm). Abbreviations: CSR = Cold, stroma-rich; CTR = Cold, tumor-rich; HTCC = Hot, tumor-centric clustering; HD = Host and disperse; HSCC = Hot, stroma-centric clustering; HC = Hot and clustered; HCTR = Host and clustered, tumor-rich; HCSR = Hot and clustered, stroma-rich. ; HR = hazard ratio; CI = confidence interval.

(a)

		HR (95% CI)	P values
$CD3^+$ T-cell density	1st quartile	Reference	
	2nd quartile	1.10 (0.81-1.51)	0.539
	3rd quartile	1.11 (0.68-1.80)	0.673
	4th quartile	1.41 (0.69-2.86)	0.348
TIPC cluster	CSR	0.96 (0.70-1.31)	0.778
	CTR	Reference	
	HTCC	0.57 (0.34-0.96)	0.035
	HD	0.35 (0.16-0.74)	0.007
	HSCC	0.74 (0.39-1.42)	0.367
	HC	0.42 (0.19-0.93)	0.033

(b)

		HR	P values
Morisita-Horn index	1st quartile	Reference	
	2nd quartile	1.11 (0.79-1.56)	0.547
	3rd quartile	1.18 (0.81-1.72)	0.399
	4th quartile	0.79 (0.48-1.31)	0.362
TIPC cluster	CSR	1.02 (0.73-1.42)	0.90
	CTR	Reference	
	HTCC	0.68 (0.43-1.06)	0.085
	HD	0.54 (0.34-0.87)	0.011
	HSCC	0.87 (0.54-1.41)	0.577
	HC	0.47 (0.23-0.95)	0.034

(c)

		HR	P values
G-cross	1st quartile	Reference	
	2nd quartile	1.26 (0.91-1.75)	0.161
	3rd quartile	1.44 (0.98-2.12)	0.064
	4th quartile	1.57 (0.93-2.65)	0.093
TIPC cluster	CSR	0.94 (0.69-1.28)	0.67
	CTR	Reference	
	HTCC	0.52 (0.34-0.80)	0.003
	HD	0.35 (0.20-0.61)	<0.001
	HSCC	0.68 (0.40-1.17)	0.161
	HC	0.36 (0.17-0.76)	0.007

(d)

		HR	P values
L-cross	1st quartile	Reference	
	2nd quartile	1.04 (0.73-1.48)	0.849
	3rd quartile	1.28 (0.88-1.85)	0.195
	4th quartile	1.39 (0.98-1.99)	0.066
TIPC cluster	CSR	0.98 (0.72-1.33)	0.88
	CTR	Reference	
	HTCC	0.56 (0.37-0.83)	0.005
	HD	0.41 (0.27-0.62)	<0.001
	HSCC	0.79 (0.49-1.28)	0.347
	HC	0.41 (0.20-0.82)	0.012
