# Supplementary material for: Tumor-immune partitioning and clustering algorithm for identifying tumor-immune cell spatial interaction signatures within the tumor microenvironment
Source: PLoS Comput Biol. 2025 Feb 18;21(2):e1012707. doi: 10.1371/journal.pcbi.1012707 (PMC11849983; doi:10.1371/journal.pcbi.1012707)
Supplement: S5 Table — Evaluation of the confounding effect of immune cell density on TIPC subtypes. Multivariable Cox proportional hazards model included TIPC subtypes of (a) cytotoxic memory T cells, (b) eosinophils, and (c) neutrophils, and the corresponding overall cell density (quartiles). Abbreviations: CSR = Cold, stroma-rich; CTR = Cold, tumor-rich; HTCC = Hot, tumor-centric clustering; HD = Host and disperse; HSCC = Hot, stroma-centric clustering; HC = Hot and clustered; HCTR = Host and clustered, tumor-rich; HCSR = Hot and clustered, stroma-rich; HR = hazard ratio; CI = confidence interval. (PDF) [file pcbi.1012707.s027.pdf]

S5 Table. Evaluation of the confounding effect of immune cell density on TIPC subtypes. Multivariable Cox proportional hazards model included TIPC subtypes of (a) cytotoxic memory T cells, (b) eosinophils, and (c) neutrophils, and the corresponding overall cell density (quartiles). Abbreviations: CSR = Cold, stroma-rich; CTR = Cold, tumor-rich; HTCC = Hot, tumor-centric clustering; HD = Host and disperse; HSCC = Hot, stroma-centric clustering; HC = Hot and clustered; HCTR = Host and clustered, tumor-rich; HCSR = Hot and clustered, stroma-rich; HR = hazard ratio; CI = confidence interval.

(a)

		HR (95% CI)	p values
$CD3^+CD8^+CD45RO^+$ T-cell density	1st quartile	Reference	
	2nd quartile	1.33 (0.99-1.80)	0.061
	3rd quartile	0.94 (0.67-1.34)	0.749
	4th quartile	1.01 (0.47-2.19)	0.975
TIPC cluster	CSR	1.01 (0.76-1.33)	0.970
	CTR	Reference	
	HSCC	1.07 (0.47-2.44)	0.879
	HTCC	0.65 (0.31-1.34)	0.239
	HD	0.36 (0.11-1.14)	0.083
	HC	0.31 (0.11-0.86)	0.025

(b)

		HR	p values
Eosinophil density	1st quartile	Reference	
	2nd quartile	0.98 (0.72-1.32)	0.874
	3rd quartile	0.75 (0.52-1.09)	0.129
	4th quartile	0.89 (0.54-1.45)	0.628
TIPC cluster	CSR	Reference	
	HCTR	0.35 (0.22-0.56)	<0.001
	CTR	0.61 (0.46-0.80)	<0.001
	HD	0.43 (0.23-0.82)	0.010
	HC	0.49 (0.25-0.95)	0.035

(c)

		HR	p values
Neutrophil density	1st quartile	Reference	
	2nd quartile	0.74 (0.53-1.02)	0.066
	3rd quartile	0.70 (0.49-1.01)	0.059
	4th quartile	0.82 (0.52-1.28)	0.373
TIPC cluster	CSR	Reference	
	HD	0.41 (0.25-0.69)	0.001
	HCSR	0.71 (0.36-1.38)	0.312
	CTR	0.58 (0.43-0.78)	<0.001
	HCTR	0.50 (0.32-0.76)	0.001
