# Supplementary material for: Tumor-immune partitioning and clustering algorithm for identifying tumor-immune cell spatial interaction signatures within the tumor microenvironment
Source: PLoS Comput Biol. 2025 Feb 18;21(2):e1012707. doi: 10.1371/journal.pcbi.1012707 (PMC11849983; doi:10.1371/journal.pcbi.1012707)
Supplement: S6 Table — Qualitative descriptions for distinct tumor-immune spatial patterns identified using TIPC. (PDF) [file pcbi.1012707.s028.pdf]

S6 Table. Qualitative descriptions for distinct tumor-immune spatial patterns identified using TIPC.

**Unique spatial subtype names (abbreviations)**	**Descriptions**
Hot and disperse (HD)	Abundant immune cells dispersed across both tumor epithelial and stromal regions; enriched in “I:T high”, “I:T low”, “I:S high”, “I:S low” subregion categories.
Hot and clustered (HC)	Abundant immune cells clustered in both tumor epithelial and stromal regions; enriched in “I:T high” and “I:S high” subregion categories.
Cold, tumor-rich (CTR)	Predominance of tumor epithelial regions with uniformly few immune cells; enriched in “tumor-only” subregion categories.
Cold, stroma-rich (CSR)	Predominance of stromal regions with uniformly few immune cells; enriched in “stroma-only” subregion categories.
Hot, tumor-centric clustering (HTCC)	Predominance of tumor epithelial regions with immune cells clustered within the tumor regions; enriched in “tumor-only” and “I:T high” subregion categories.
Hot, stroma-centric clustering (HSCC)	Predominance of stromal regions with immune cells clustered within the stromal regions; enriched in “stroma-only” and “I:S high” subregion categories.
Hot and clustered, tumor-rich (HCTR)	Abundant immune cells clustered in both tumor epithelial and stromal regions, and predominance of stromal regions depleted of immune cells; enriched in “I:T high”, “I:S high”, and “tumor-only” subregion categories.
Hot and clustered, stroma-rich (HCSR)	Abundant immune cells clustered in both tumor epithelial and stromal regions, and predominance of tumor epithelial regions depleted of immune cells; enriched in “I:T high”, “I:S high”, and “stroma-only” subregion categories.
